# Synthesis of the Isodityrosine Moiety of Seongsanamide A–D and Its Derivatives

**DOI:** 10.3390/md21070373

**Published:** 2023-06-25

**Authors:** Yang Xie, Zhou Xu, Pei Hu, Xiao-Ting Tian, Yi-Hong Lu, Hao-Dong Jiang, Cheng-Gang Huang, Zhi-Cai Shang

**Affiliations:** 1 Department of Chemistry, Zhejiang University, Hangzhou 310027, China; sunshine_xie@163.com; 2 Shanghai Institute of Materia Medica, Chinese Academy of Sciences, Shanghai 201203, China; xz_619@163.com (Z.X.); hupeishtcm@126.com (P.H.); zhghydxtxt@163.com (X.-T.T.); xiong1743771101@163.com (Y.-H.L.); 18838916947@163.com (H.-D.J.)

**Keywords:** C(sp^3^)–H functionalization, palladium, arylation, isodityrosine, diaryl ether

## Abstract

The concise and highly convergent synthesis of the isodityrosine unit of seongsanamide A–D and its derivatives bearing a diaryl ether moiety is described. In this work, the synthetic strategy features palladium-catalyzed C(sp^3^)–H functionalization and a Cu/ligand-catalyzed coupling reaction. We report a practical protocol for the palladium-catalyzed mono-arylation of β-methyl C(sp^3^)–H of an alanine derivative bearing a 2-thiomethylaniline auxiliary. The reaction is compatible with a variety of functional groups, providing practical access to numerous β-aryl-α-amino acids; these acids can be converted into various tyrosine and dihydroxyphenylalanine (DOPA) derivatives. Then, a CuI/*N,N*-dimethylglycine-catalyzed arylation of the already synthesized DOPA derivatives with aryl iodides is described for the synthesis of isodityrosine derivatives.

## 1. Introduction

**Figure 1:**
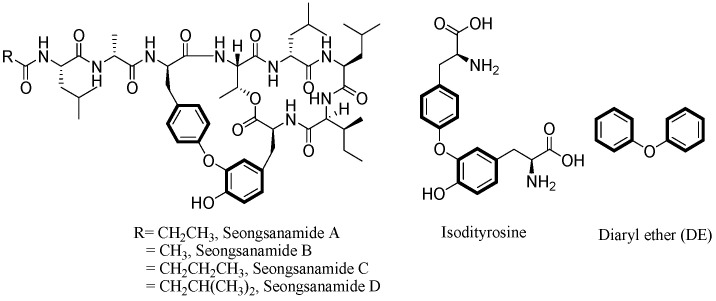
Structures of the seongsanamides A–D and isodityrosine.

In 2018, Choi and coworkers [1] reported the isolation of seongsanamide A–D from a bacterial culture broth of *Bacillus safensis* KCTC 12796BP, obtained from a marine sponge collected in water samples of Seongsan on Jeju Island. Seongsanamides are bicyclic depsipeptides with an isodityrosine residue and exhibit antiallergenic properties (Figure 1). Furthermore, isodityrosine is a tyrosine dimer containing oxidatively coupled aromatic nuclei, and the tyrosine units are linked through a diaryl ether moiety [2]. A large class of biological cyclopeptides containing this structural unit exists in nature, with a wide range of pharmaceutical activities, such as seongsanamidesA–D, K-13 [3], OF4949-Ⅲ [3], rubiyunnanin D [4], and bouvardin [5].

**Figure 2:**
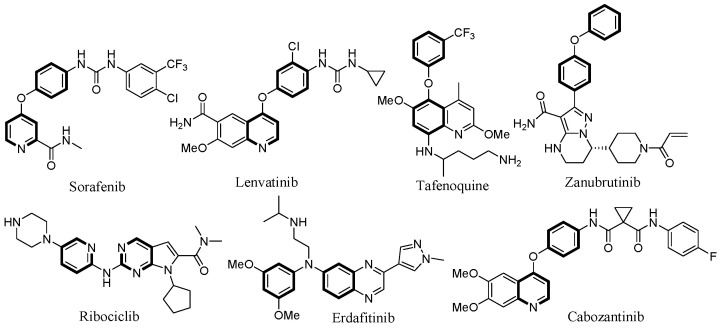
Representative drugs bearing diaryl ether and theirbioisosteres.

In addition, diaryl ether (DE) is a functional scaffold that exists widely in both natural products and new drugs approved for the market [6]. Moreover, DE is always considered the fundamental fragment of a wide variety of medicinal and agrochemical agents as well as their bioisosteres (Figure 2). Over the years, medicinal chemists have exploited the use of privileged structures inspired by natural products in drug discovery. The introduction of functional groups, such as α-keto [7] amide and *gem*-dimethyl [8] moieties, into biologically active small molecules has emerged as an efficient way to obtain clinically useful drugs.

Due to their potential structural diversity, pharmaceutical value, and good druggability, isodityrosines and cyclopeptide derivatives have attracted increasing attention in the synthetic community. Numerous research laboratories have engaged in the total synthesis of these natural products or analogs, including Dötz benzannulation [9], S_N_Ar reactions [10], and the Ullman [11,12,13,14,15,16] and Evans–Chan–Lam [3,17,18] coupling reactions. However, the synthesis of isodityrosines and their derivatives usually begins with available natural amino acids, limiting the diversity of the synthesis of their derivatives and their applications in drug design.

**Scheme 1:**
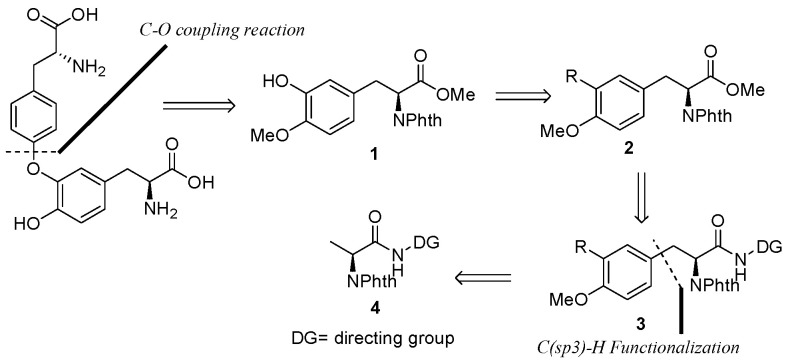
Retrosynthetic analysis of the isodityrosine moiety.

To address these challenges, we aimed to establish a convenient and convergent synthetic route. In recent years, transition-metal-catalyzed C–H functionalization has provided general and practical access to various natural and unnatural aromatic amino acids [19,20,21,22,23]. Our retrosynthetic analysis of the isodityrosine moiety of seongsanamides is outlined in Scheme 1. We selected a strategy based on palladium-catalyzed C(sp^3^)–H functionalization and copper-catalyzed Ullmann coupling reactions. The iodityrosine moiety was prepared from key precursor **1** and 4-iodophenylalaninederivatives via a C–O coupling reaction. The DOPA derivative **1** was derived from intermediate **2** by removing hydroxyl protecting groups, Dakin oxidation of aldehyde, or Baeyer–Villiger oxidation of acetyl group. Compound **2** was prepared by removing the directing group of **3**, which resulted from the palladium-catalyzed monoarylation of β-methyl C(sp^3^)–H of an alanine derivative with aryl iodides using a directing group. The structural diversity of aryl iodides could facilitate the synthesis of more isodityrosine derivatives.

## 2. Results

Our study began with the synthesis of *N*-phthaloylalanine derivatives **4a**–**c** to examine potential amide directing groups (phthaloyl = Phth). Several commercially available aniline-based auxiliary groups, including 8-aminoquinoline [24,25,26], 2-thiomethylaniline (ArS) [27], and *O*-methylhydroxylamine [28], were selected for the palladium-catalyzed arylation. Subsequently, the treatment of 2-methoxybenzaldehyde with *N*-iodosuccinimide(NIS) and FeCl_3_ produced the corresponding aryl iodide**5a**,with a yield of 89%.

**Scheme 2:**
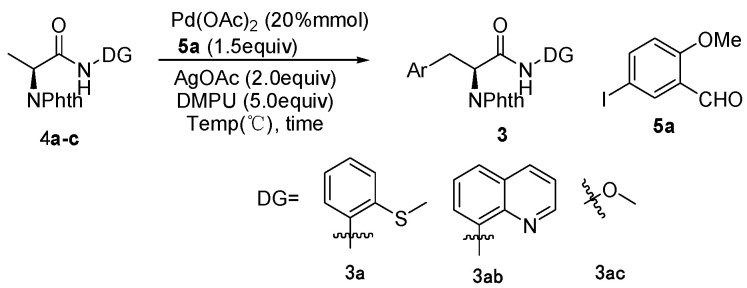
Palladium-catalyzed arylation of **4a**–**c** with **5a**.

Initially, the selectivity for mono- versus diarylation needed to be controlled. We initiated our investigation with the selective methyl C(sp^3^)–H monoarylation of alanine derivative with aryl iodide (**5a**) as the model system (Scheme 2). In 2012, Daugulis reported that the selective β-monoarylation of alanine derivatives occurred under solvent-free conditions in high yield using a 2-thiomethylaniline auxiliary [27]. In 2014, Bull reported that the 3-monoarylation of proline derivatives under solvent-free conditions with 8-aminoquinoline directing groups was also successful [26]. Previously, Shi reported that the use of coordinating solvents, such as dimethylamine (DMA) and dimethylpropyleneurea(DMPU), improved the selectivity in the arylation of alanine derivatives with aryl iodides [29].

Due to these studies, we selected solvent-free conditions using palladium (II) acetate (Pd(OAc)_2_, 20 mol%), silver acetate (AgOAc, 1.5 equiv), and DMPU (5.0 equiv) as additives. In accordance with reported results, the arylation of **4b** bearing an 8-aminoquinoline auxiliary mainly produced diarylated product **3ab** when the reaction was conducted at 150 °C, and **4a** bearing a 2-thiomethylaniline auxiliary mainly produced monoarylated product **3a** (Table 1, entries 1 and 2). The optimization of this transformation continued with **4a**. We aimed to maximize the yield of **3a** and the selectivity of monoarylation. We found that lowering the reaction temperature to 100 °C improved both the yield and selectivity of the reaction (48% yield, mono/di 12:1; Table 1, entry 3). Further screening showed that running the reaction at 80 °C increased both mono-selectivity and yield (62% yield, Table 1, entry 4). However, no satisfactory result was obtained when the reaction was run at 60 °C (42% yield, Table 1, entry 5).

Additional optimization experiments showed that the more strongly coordinating 8-aminoquinoline auxiliary still provided a predominantly diarylated product at 80 °C (mono/di 1:1.7; Table 1, entry 6). Interestingly, the palladium-catalyzed C(sp^3^)–H arylation of Weinreb amides **4c** bearing an *N*-methoxyamide auxiliary was also successful, with a 36% yield(Table 1, entry 7). Notably, the reaction was also successful on a larger scale, and the yields were similar to those obtained on a smaller scale (Table 1, entries 8 and 9).

**Scheme 3:**
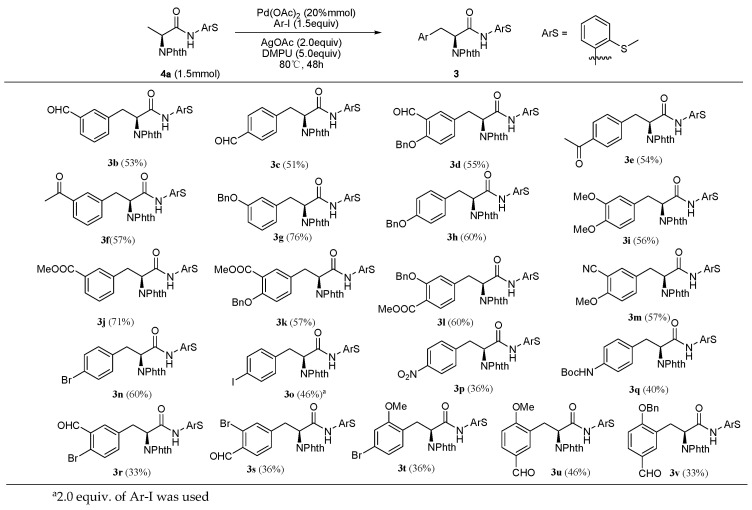
Palladium-catalyzed monoarylation of **4a** with aryl iodides.

With the optimized the reaction conditions for the β-monoarylation of alanine-derived amide **4a**, we next investigated the scope of alkyl iodides that were compatible in the reaction (Scheme 3). A wide range of aryl iodides with different electron-withdrawing functional groups, such as formyl (**3b**, 53%; **3c**, 51%), acetyl (**3e**, 54%; **3f**, 57%), bromo (**3n**, 60%), and methoxycarbonyl (**3j**, 71%) groups underwent efficient monoarylation to produce the corresponding substituted phenylalanines in moderate yields (see Supplementary Materials). Moreover, aryl iodide bearing a nitro (**3p**) substituent was less reactive and successful, affording a 36% yield. Additionally, aryl iodides bearing alkoxy (**3g**, 76%; **3h**, 60%; **3i**, 56%) substituents were compatible with this protocol to produce their desired products in moderate to good yields.

Importantly, 3,4-disubstituted aryl iodides bearing one alkoxy group and an electron-withdrawing functional group also reacted smoothly under the standard conditions to produce the β-monoarylation products in moderate yields (**3d**, 55%; **3k**, 57%; **3l**, 60%; **3m**, 57%). In general, aryl iodides carrying electron-donating groups are more reactive than aryl iodides bearing electron-withdrawing groups. Also proving to be compatible were 3,4-Disubstituted aryl iodides, albeit with reduced yields (**3r**, 33%; **3s**, 36%).

However, an unfavorable steric effect was also apparent in 2-substituted aryl iodides, and decreased reactivity was also evident; sterically hindered immediately adjacent to the iodide decreased reactivity. Notably, steric aryl iodides were also compatible with the reaction conditions, although they were less reactive (**3t**, 36%; **3u**, 46%; **3v**, 33%). Interestingly, the 4-tert-butoxycarbonylamino- and 4-iodo-aryl derivatives were also successful, providing handles for potential further functionalization (**3o**, 46%; **3q**, 40%).

**Scheme 4:**
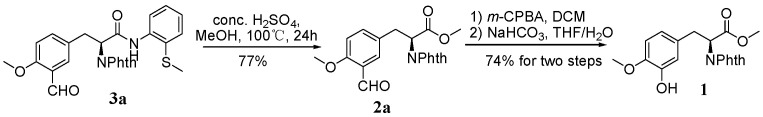
Synthesis of DOPA derivative **1**.

Based on our access to a wide range of phenylalanine derivatives, we subsequently examined the removal of the directing group. The sequential transformation of formyl, acetyl, and alkoxy groups to hydroxyl groups provided various tyrosine and DOPA derivatives that could be used to produce more isodityrosine derivatives via the Ullmann coupling reaction. The directing group of **3a** was easily removed by treatment with H_2_SO_4_ in methanol (MeOH) to produce the corresponding methyl ester **2a** with a 77% yield (Scheme 4). The treatment of aldehyde **2a** with *m*-CPBA produced the formate ester, which was immediately converted into the corresponding phenol **1** (74% yield for two steps).

**Scheme 5:**
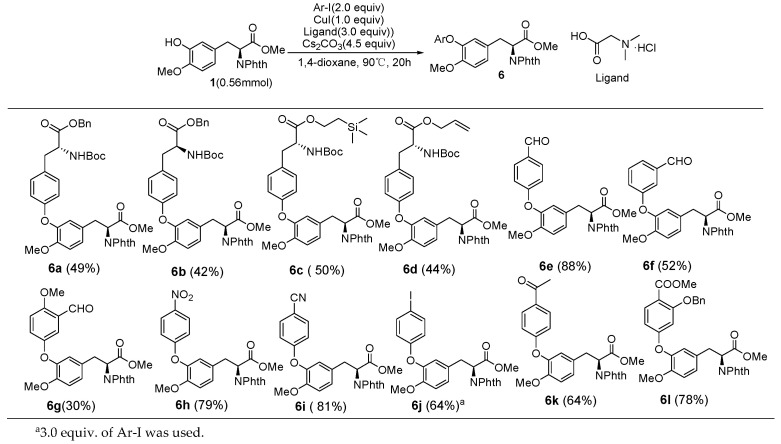
Coupling of the aryl iodides with DOPA derivative **1**.

Subsequently, we focused on the second key step, the copper-catalyzed Ullmann coupling reaction of DOPA derivative **1** (Scheme 5, see Supplementary Materials). The development of useful methods via the Cu/ligand-catalyzed arylation of phenols for assembling diaryl ethers was accomplished [30,31,32,33,34]. Initially, we selected the coupling of phenylalanine-derived aryl iodides (1.5 equiv), with **1** as a model reaction to optimize the reaction conditions. The coupling reaction was tested in the presence of CuI (1.0 equiv) and Cs_2_CO_3_ (4.5 equiv) when the reaction was carried out in 1,4-dioxane (0.1 M) under N_2_ at 90 °C for 20 h. We tested several ligands that are known to promote copper-catalyzed coupling reactions. Interestingly, we found that using commercially available *N,N*-dimethylglycine as a ligand produced the corresponding products. Moreover, several phenylalanine-derived aryl iodides with different protecting groups at the C-terminus were compatible with the reaction conditions (**6a**, 49%; **6b**, 50%; **6c**, 44%). Notably, in isodityrosine derivatives **6a**–**d**, all amino and carboxylate groups with different protections enabled the selective manipulation of the individual functionalities.

With the optimized reaction conditions, we next examined an extensive range of aryl iodides to produce a 3-aryloxyphenylalanine derivative bearing a diaryl ether moiety. A variety of functional groups of aryl iodides are known to tolerate this reaction condition, including alkoxyl, formyl, acetyl, nitro, carbonyl, iodo, and cyano groups. Both electron-rich and electron-deficient aryl iodides underwent this reaction to produce the corresponding diaryl ethers in good yields. A high yield was provided by 4-iodobenzaldehyde (**6e**, 88%), whereas 3-iodobenzaldehyde provided a 52% yield under standard conditions. Furthermore, aromatics with aldehyde and methoxyl groups were also compatible, with reduced yields (**6g**, 30%). Notably, the reaction of 1,4-diiodobenzene with phenol **1** produced the coupling product **6j** in 64% yield, which was a 3-(4-iodoaryloxy)-phenylalanine derivative and provided a handle for potential further functionalization.

In conclusion, we have developed a convenient and highly convergent synthesis of the isodityrosine unit of seongsanamide A–D and its derivatives bearing a diaryl ether moiety. The synthetic sequence is based on a palladium-catalyzed C(sp^3^)–H functionalization and a Cu/ligand-catalyzed coupling reaction. Initially, we developed a Pd-catalyzed mono-arylation of β-methyl C(sp^3^)–H of an alanine derivative using the 2-thiomethylaniline directing group. A wide range of aryl iodides could be applied in this protocol to provide various aromatic α-amino acid compounds; these compounds could be used for the synthesis of various tyrosine and DOPA derivatives. Subsequently, the CuI/*N,N*-dimethylglycine-catalyzed coupling of DOPA derivatives with aryl iodides was accomplished to prepare diaryl ethers and the synthesis of isodityrosine derivatives. Our convergent synthetic method facilitates a concise way for the efficient preparation of various DE-based analogs for drug development efforts. The specific introduction of DE-based units into biologically active small molecules and a study of the impact on the physicochemical properties and potential biological activities are currently being performed.

## 3. Materials and Methods

### 3.1. General Experimental Methods

Chemicals were acquired from commercial sources and used as received. Nuclear magnetic resonance (NMR) spectra were recorded on Bruker Ascend-400 and Bruker Ascend-500 spectrometers at the following spectrometer frequencies. Multiplicities are assigned as s (singlet), d (doublet), t (triplet), q (quartet), m (multiplet), and app (apparent).The exact mass was obtained using a time-of-flight (TOF) detector on Agilent 6530-Q-TOF. Thin layer chromatography (TLC) was carried out with 0.2 mm thick silica gel plates. Visualization was accomplished by using UV light. Column chromatography was performed on silica gel (200–300 mesh).

### 3.2. Synthesis of the Phenylalanine Derivatives3a–v

A Schlenk tube was charged with 1 (1.5 mmol), ), aryl iodide (1.5 equiv., or 2.0 equiv. used for synthesis of **3o**), Pd(OAc)_2_ (0.2 equiv.), AgOAc (2.0 equiv.), and DMPU (5.0 equiv.), evacuated, and backfilled with N_2_. The reaction mixture was stirred at 80 °C for 48 h. The suspension was filtered, and the filtrate was concentrated, followed by column chromatography on silica gel (eluting with 1:5 to 1:2 ethyl acetate/petroleum ether) to provide the desired product.

*(S)-N-(3-(4-Methoxy-3-formylphenyl)-2-phthalimidopropionyl)-2-methylthioaniline* (**3a**). **3a** was obtained as a solid. ^1^H NMR (400 MHz, CDCl_3_) δ 10.30 (s, 1H), 8.94 (s, 1H), 8.31 (d, *J* = 8.1 Hz, 1H), 7.82 (dt, *J* = 7.1, 3.5 Hz, 2H), 7.79–7.67 (m, 2H), 7.64 (d, *J* = 2.2 Hz, 1H), 7.52–7.38 (m, 2H), 7.29 (dd, *J* = 13.5, 6.0 Hz, 1H), 7.06 (td, *J* = 7.6, 1.0 Hz, 1H), 6.86 (d, *J* = 8.6 Hz, 1H), 5.24 (dd, *J* = 10.6, 5.9 Hz, 1H), 3.84 (s, 3H), 3.68 (qd, *J* = 14.3, 8.3 Hz, 2H), 2.20 (s, 3H); ^13^C NMR (101 MHz, CDCl_3_) δ 189.45, 167.81, 166.20, 160.89, 137.97, 136.49, 134.63, 133.53, 131.43, 129.33, 129.13, 129.08, 125.64, 124.97, 124.82, 123.82, 120.70, 112.27, 56.30, 55.79, 33.40, 19.22; HRMS(ESI): *m*/*z* [M + H]^+^calcd. for C_26_H_23_N_2_O_5_S^+^: 475.1322, found: 475.1448.

*(S)-N-(3-(3-Formylphenyl)-2-phthalimidopropionyl)-2-methylthioaniline* (**3b**). **3b** was obtained as an oil. ^1^H NMR (500 MHz, CDCl_3_) δ 9.88 (s, 1H), 8.95 (s, 1H), 8.33 (d, *J* = 8.2 Hz, 1H), 7.81 (dt, *J* = 7.4, 3.7 Hz, 2H), 7.76–7.71 (m, 3H), 7.68 (d, *J* = 7.6 Hz, 1H), 7.52 (d, *J* = 7.6 Hz, 1H), 7.45 (t, *J* = 8.5 Hz, 1H), 7.39 (t, *J* = 7.6 Hz, 1H), 7.31 (t, *J* = 7.8 Hz, 1H), 7.07 (t, *J* = 7.6 Hz, 1H), 5.33 (dd, *J* = 10.9, 5.7 Hz, 1H), 3.80 (ddd, *J* = 25.2, 14.3, 8.4 Hz, 2H), 2.18 (s, 3H); ^13^C NMR (126 MHz, CDCl_3_) δ 192.11, 167.76, 166.03, 138.13, 137.90, 136.85, 135.14, 134.73, 133.56, 131.33, 130.59, 129.57, 129.39, 128.34, 125.59, 125.04, 123.83, 120.66, 56.01, 34.14, 19.22; HRMS(ESI): *m*/*z* [M + H]^+^calcd. for C_25_H_21_N_2_O_4_S^+^: 445.1217, found: 445.1223.

*(S)-N-(3-(4-Formylphenyl)-2-phthalimidopropionyl)-2-methylthioaniline* (**3c**). **3c** was obtained as an oil. ^1^H NMR (500 MHz, CDCl_3_) δ 9.91 (d, *J* = 11.3 Hz, 1H), 8.94 (s, 1H), 8.32 (d, *J* = 8.2 Hz, 1H), 7.81 (dt, *J* = 7.5, 3.8 Hz, 2H), 7.78–7.70 (m, 4H), 7.44 (d, *J* = 7.7 Hz, 1H), 7.39 (d, *J* = 7.9 Hz, 2H), 7.31 (t, *J* = 7.8 Hz, 1H), 7.07 (t, *J* = 7.6 Hz, 1H), 5.34 (dd, *J* = 10.7, 6.0 Hz, 1H), 3.87–3.72 (m, 2H), 2.17 (s, 3H); ^13^C NMR (126 MHz, CDCl_3_) δ 191.94, 167.71, 165.95, 144.18, 137.87, 135.37, 134.77, 133.57, 131.28, 130.25, 129.78, 129.41, 125.57, 125.07, 123.85, 120.63, 55.76, 34.56, 19.21; HRMS(ESI): *m*/*z* [M + H]^+^calcd. for C_25_H_21_N_2_O_4_S^+^: 445.1217, found: 445.1221.

*(S)-N-(3-(4-(Benzyloxy)-3-formylphenyl)-2-phthalimidopropionyl)-2-methylthioaniline*(**3d**). **3d** was obtained as a solid. ^1^H NMR (500 MHz, CDCl_3_) δ 10.40 (s, 1H), 8.94 (s, 1H), 8.32 (d, *J* = 8.2 Hz, 1H), 7.82 (dt, *J* = 7.3, 3.7 Hz, 2H), 7.76–7.70 (m, 2H), 7.67 (d, *J* = 2.1 Hz, 1H), 7.47–7.41 (m, 2H), 7.38 (d, *J* = 4.3 Hz, 4H), 7.36–7.32 (m, 1H), 7.30 (t, *J* = 7.6 Hz, 1H), 7.06 (t, *J* = 7.5 Hz, 1H), 6.93 (d, *J* = 8.6 Hz, 1H), 5.24 (dd, *J* = 10.7, 5.7 Hz, 1H), 5.10 (s, 2H), 3.68 (ddd, *J* = 25.1, 14.4, 8.3 Hz, 2H), 2.20 (s, 3H); ^13^C NMR (126 MHz, CDCl_3_) δ 189.36, 167.80, 166.18, 160.11, 137.96, 136.45, 136.03, 134.63, 133.55, 131.43, 129.52, 129.35, 128.91, 128.83, 128.41, 127.42, 125.61, 125.16, 124.97, 123.83, 120.69, 70.62, 56.27, 33.43, 19.23; HRMS(ESI): *m*/*z* [M + H]^+^calcd. for C_32_H_27_N_2_O_5_S^+^: 551.1635, found: 551.1674.

*(S)-N-(3-((4-Acetylphenyl)-2-phthalimidopropionyl)-2-methylthioaniline* (**3e**). **3e** was obtained as an oil. ^1^H NMR (500 MHz, CDCl_3_) δ 8.95 (s, 1H), 8.33 (d, *J* = 8.1 Hz, 1H), 7.81 (dt, *J* = 9.7, 4.8 Hz, 3H), 7.79 (s, 1H), 7.73 (dd, *J* = 5.3, 3.1 Hz, 2H), 7.44 (d, *J* = 7.6 Hz, 1H), 7.31 (t, *J* = 8.4 Hz, 3H), 7.07 (t, *J* = 7.5 Hz, 1H), 5.70–4.91 (m, 1H), 4.21–3.39 (m, 2H), 2.52 (s, 3H), 2.18 (s, 3H); ^13^C NMR (126 MHz, CDCl_3_) δ 197.86, 167.76, 166.08, 142.55, 137.93, 136.05, 134.73, 133.60, 131.34, 129.42, 129.31, 128.91, 125.56, 125.04, 123.85, 120.64, 55.88, 34.36, 26.70, 19.24; HRMS(ESI): *m*/*z* [M + H]^+^calcd. for C_26_H_23_N_2_O_4_S^+^: 459.1373, found: 459.1376.

*(S)-N-(3-(3-Acetylphenyl)-2-phthalimidopropionyl)-2-methylthioaniline* (**3f**). **3f** was obtained as an oil. ^1^H NMR (500 MHz, CDCl_3_) δ 8.96 (s, 1H), 8.33 (d, *J* = 8.2 Hz, 1H), 7.84–7.79 (m, 2H), 7.78 (s, 1H), 7.76–7.70 (m, 3H), 7.44 (d, *J* = 7.7 Hz, 2H), 7.35–7.28 (m, 2H), 7.07 (td, *J* = 7.6, 1.0 Hz, 1H), 5.32 (dd, *J* = 10.9, 5.7 Hz, 1H), 3.78 (ddd, *J* = 25.2, 14.3, 8.4 Hz, 2H), 2.47 (s, 3H), 2.19 (s, 3H); ^13^C NMR (126 MHz, CDCl_3_) δ 197.92, 167.78, 166.15, 137.92, 137.54, 137.43, 134.70, 133.79, 133.52, 131.37, 129.36, 129.16, 129.03, 127.08, 125.63, 125.02, 123.79, 120.70, 56.13, 34.25, 26.71, 19.21; HRMS(ESI): *m*/*z* [M + H]^+^calcd. for C_26_H_23_N_2_O_4_S^+^: 459.1373, found: 459.1378.

*(S)-N-(3-(3-(Benzyloxy)phenyl)-2-phthalimidopropionyl)-2-methylthioaniline* (**3g**). **3g** was obtained as an oil. ^1^H NMR (400 MHz, CDCl_3_) δ 8.96 (s, 1H), 8.34 (d, *J* = 8.1 Hz, 1H), 7.86–7.79 (m, 2H), 7.75–7.68 (m, 2H), 7.47–7.40 (m, 1H), 7.37–7.33 (m, 4H), 7.33–7.27 (m, 2H), 7.13 (dd, *J* = 14.3, 6.4 Hz, 1H), 7.07 (td, *J* = 7.6, 1.2 Hz, 1H), 6.90–6.80 (m, 2H), 6.76 (dd, *J* = 8.2, 2.1 Hz, 1H), 5.33 (dd, *J* = 10.7, 5.9 Hz, 1H), 4.91 (q, *J* = 11.6 Hz, 2H), 3.70 (qd, *J* = 14.2, 8.3 Hz, 2H), 2.20 (s, 3H); ^13^C NMR (126 MHz, CDCl_3_) δ 167.86, 166.46, 159.06, 138.31, 138.01, 136.92, 134.52, 133.51, 131.51, 129.87, 129.31, 128.63, 128.03, 127.54, 125.59, 124.91, 123.72, 121.59, 120.68, 115.25, 113.85, 69.93, 56.31, 34.49, 19.19; HRMS(ESI): *m*/*z* [M + H]^+^calcd. for C_31_H_27_N_2_O_4_S^+^: 523.1686, found: 523.1734.

*(S)-N-(3-(4-(Benzyloxy)phenyl)-2-phthalimidopropionyl)-2-methylthioaniline* (**3h**). **3h** was obtained as an oil. ^1^H NMR (500 MHz, CDCl_3_) δ 8.96 (s, 1H), 8.34 (d, *J* = 8.2 Hz, 1H), 7.86–7.79 (m, 2H), 7.75–7.69 (m, 2H), 7.45 (d, *J* = 7.6 Hz, 1H), 7.39–7.32 (m, 4H), 7.30 (dd, *J* = 10.4, 4.8 Hz, 2H), 7.14 (d, *J* = 8.5 Hz, 2H), 7.06 (t, *J* = 7.6 Hz, 1H), 6.81 (d, *J* = 8.6 Hz, 2H), 5.27 (dd, *J* = 10.6, 6.1 Hz, 1H), 4.96 (s, 2H), 3.74–3.60 (m, 2H), 2.20 (s, 3H); ^13^C NMR (126 MHz, CDCl_3_) δ 167.92, 166.58, 157.84, 138.08, 137.00, 134.50, 133.55, 131.53, 130.08, 129.33, 128.93, 128.66, 128.06, 127.58, 125.58, 124.89, 123.72, 120.71, 115.19, 70.04, 56.56, 33.67, 19.21; HRMS(ESI): *m*/*z* [M + H]^+^ calcd. for C_31_H_27_N_2_O_4_S^+^: 523.1686, found: 523.1700.

*(S)-N-(3-(3,4-Dimethoxyphenyl)-2-phthalimidopropionyl)-2-methylthioaniline* (**3i**). **3i** was obtained as a solid. ^1^H NMR (500 MHz, CDCl_3_) δ 8.98 (s, 1H), 8.33 (d, *J* = 8.2 Hz, 1H), 7.81 (dt, *J* = 7.5, 3.8 Hz, 2H), 7.76–7.69 (m, 2H), 7.44 (d, *J* = 7.7 Hz, 1H), 7.30 (t, *J* = 7.8 Hz, 1H), 7.06 (t, *J* = 7.6 Hz, 1H), 6.76 (d, *J* = 8.1 Hz, 1H), 6.69 (d, *J* = 7.9 Hz, 2H), 5.29 (dd, *J* = 10.9, 6.0 Hz, 1H), 3.77 (s, 3H), 3.70 (s, 3H), 3.69–3.60 (m, 2H), 2.21 (s, 3H); ^13^C NMR (126 MHz, CDCl_3_) δ 167.93, 166.59, 148.94, 147.94, 138.03, 134.57, 133.48, 131.51, 129.31, 129.03, 125.64, 124.93, 123.69, 121.20, 120.73, 111.84, 111.38, 56.53, 55.87, 55.80, 34.03, 19.16; HRMS(ESI): *m*/*z* [M + H]^+^calcd. for C_26_H_25_N_2_O_5_S^+^: 477.1479, found: 477.1492.

*(S)-N-(3-(3-(Methoxycarbonyl)phenyl)-2-phthalimidopropionyl)-2-methylthioaniline* (**3j**). **3j** was obtained as an oil. ^1^H NMR (500 MHz, CDCl_3_) δ 8.95 (s, 1H), 8.33 (d, *J* = 8.2 Hz, 1H), 7.89 (s, 1H), 7.82 (dt, *J* = 9.7, 4.9 Hz, 3H), 7.77–7.70 (m, 2H), 7.43 (t, *J* = 8.0 Hz, 2H), 7.30 (dt, *J* = 15.8, 7.9 Hz, 2H), 7.06 (t, *J* = 7.6 Hz, 1H), 5.31 (dd, *J* = 10.8, 5.6 Hz, 1H), 3.83 (s, 3H), 3.76 (ddd, *J* = 25.1, 14.3, 8.4 Hz, 2H), 2.19 (s, 3H); ^13^C NMR (126 MHz, CDCl_3_) δ 167.78, 166.83, 166.17, 137.97, 137.25, 134.60, 133.58, 131.42, 130.68, 130.18, 129.37, 128.93, 128.47, 125.58, 124.98, 123.79, 120.68, 56.19, 52.22, 34.19, 19.22; HRMS(ESI): *m*/*z* [M + H]^+^calcd. for C_26_H_21_N_2_O_5_S^+^: 475.1322, found: 475.1326.

*(S)-N-(3-(4-(Benzyloxy)-3-(methoxycarbonyl)phenyl)-2-phthalimidopropionyl)-2-methylthioaniline* (**3k**). **3k** was obtained as an oil. ^1^H NMR (500 MHz, CDCl_3_) δ 8.94 (s, 1H), 8.33 (d, *J* = 8.2 Hz, 1H), 7.85–7.79 (m, 2H), 7.76–7.70 (m, 2H), 7.65 (d, *J* = 2.3 Hz, 1H), 7.47–7.40 (m, 3H), 7.35 (dd, *J* = 10.3, 4.8 Hz, 2H), 7.33–7.27 (m, 3H), 7.06 (td, *J* = 7.6, 1.3 Hz, 1H), 6.88 (d, *J* = 8.6 Hz, 1H), 5.26 (dd, *J* = 10.6, 6.0 Hz, 1H), 5.09 (s, 2H), 3.79 (s, 3H), 3.67 (qd, *J* = 14.4, 8.3 Hz, 2H), 2.20 (s, 3H); ^13^C NMR (126 MHz, CDCl_3_) δ 167.85, 166.30, 166.24, 157.32, 138.01, 136.73, 134.58, 133.97, 133.56, 132.37, 131.49, 129.35, 128.84, 128.63, 127.88, 126.89, 125.61, 124.95, 123.80, 120.78, 120.71, 114.41, 70.71, 56.28, 52.06, 33.41, 19.21; HRMS(ESI): *m*/*z* [M + H]^+^calcd. for C_33_H_29_N_2_O_6_S^+^: 581.1741, found: 581.1753.

*(S)-N-(3-(3-(Benzyloxy)-4-(methoxycarbonyl)phenyl)-2-phthalimidopropionyl)-2-methylthioaniline* (**3l**). **3l** was obtained as an oil. ^1^H NMR (500 MHz, CDCl_3_) δ 8.95 (s, 1H), 8.33 (d, *J* = 8.1 Hz, 1H), 7.87–7.80 (m, 2H), 7.73 (td, *J* = 5.2, 2.1 Hz, 2H), 7.70 (t, *J* = 5.5 Hz, 1H), 7.45 (d, *J* = 7.5 Hz, 3H), 7.37 (dd, *J* = 10.3, 4.7 Hz, 2H), 7.34–7.27 (m, 2H), 7.07 (td, *J* = 7.6, 1.3 Hz, 1H), 6.90 (s, 1H), 6.88 (d, *J* = 7.9 Hz, 1H), 5.34 (dd, *J* = 9.5, 7.3 Hz, 1H), 5.02 (dd, *J* = 41.7, 11.9 Hz, 2H), 3.83 (s, 3H), 3.74 (dd, *J* = 12.0, 4.7 Hz, 2H), 2.19 (s, 3H); ^13^C NMR (126 MHz, CDCl_3_) δ 167.80, 166.51, 166.13, 158.58, 143.11, 137.92, 136.65, 134.75, 133.55, 132.50, 131.39, 129.39, 128.64, 127.90, 126.92, 125.62, 125.05, 123.86, 121.26, 120.67, 119.34, 114.41, 70.69, 55.75, 52.05, 34.52, 19.20; HRMS(ESI): *m*/*z* [M + H]^+^calcd. for C_33_H_29_N_2_O_6_S^+^: 581.1741, found: 581.1752.

*(S)-N-(3-(3-Cyano-4-methoxyphenyl)-2-phthalimidopropionyl)-2-methylthioaniline* (**3m**). **3m** was obtained as a solid. ^1^H NMR (500 MHz, CDCl_3_) δ 8.91 (s, 1H), 8.30 (d, *J* = 8.2 Hz, 1H), 7.84 (dt, *J* = 7.4, 3.7 Hz, 2H), 7.79–7.73 (m, 2H), 7.43 (d, *J* = 8.4 Hz, 2H), 7.37 (d, *J* = 1.8 Hz, 1H), 7.30 (t, *J* = 7.8 Hz, 1H), 7.06 (t, *J* = 7.6 Hz, 1H), 6.84 (d, *J* = 8.7 Hz, 1H), 5.22 (dd, *J* = 10.8, 5.7 Hz, 1H), 3.84 (s, 3H), 3.66 (ddd, *J* = 25.3, 14.4, 8.3 Hz, 2H), 2.18 (s, 3H); ^13^C NMR (126 MHz, CDCl_3_) δ 167.76, 165.92, 160.36, 137.79, 135.05, 134.83, 134.06, 133.47, 131.26, 129.48, 129.33, 125.63, 125.07, 123.91, 120.65, 116.21, 111.81, 101.95, 56.13, 55.96, 33.15, 19.17; HRMS(ESI): *m*/*z* [M + H]^+^calcd. for C_26_H_22_N_3_O_4_S^+^: 472.1326, found: 472.1341.

*(S)-N-(3-(4-Bromophenyl)-2-phthalimidopropionyl)-2-methylthioaniline* (**3n**). **3n** was obtained as an oil. ^1^H NMR (500 MHz, CDCl_3_) δ 8.93 (s, 1H), 8.32 (d, *J* = 8.2 Hz, 1H), 7.83 (dt, *J* = 7.2, 3.6 Hz, 2H), 7.78–7.70 (m, 2H), 7.44 (d, *J* = 7.6 Hz, 1H), 7.32 (d, *J* = 8.3 Hz, 2H), 7.30 (d, *J* = 7.6 Hz, 1H), 7.10 (d, *J* = 8.3 Hz, 2H), 7.07 (t, *J* = 7.6 Hz, 1H), 5.27 (dd, *J* = 10.3, 6.4 Hz, 1H), 3.73–3.64 (m, 2H), 2.19 (s, 3H); ^13^C NMR (126 MHz, CDCl_3_) δ 167.82, 166.19, 137.95, 135.82, 134.70, 133.59, 131.95, 131.39, 130.77, 129.40, 125.55, 125.01, 123.85, 121.09, 120.65, 56.03, 33.86, 19.24; HRMS(ESI): *m*/*z* [M + H]^+^calcd. for C_24_H_20_BrN_2_O_3_S^+^: 495.0373, found: 495.0375.

*(S)-N-(3-(4-Iodophenyl)-2-phthalimidopropionyl)-2-methylthioaniline* (**3o**). **3o** was obtained as an oil. ^1^H NMR (400 MHz, CDCl_3_) δ 8.92 (s, 1H), 8.31 (d, *J* = 8.2 Hz, 1H), 7.82 (dt, *J* = 7.1, 3.6 Hz, 2H), 7.77–7.71 (m, 2H), 7.52 (d, *J* = 8.2 Hz, 2H), 7.44 (d, *J* = 7.7 Hz, 1H), 7.30 (t, *J* = 7.8 Hz, 1H), 7.06 (t, *J* = 7.6 Hz, 1H), 6.98 (d, *J* = 8.2 Hz, 2H), 5.32–5.23 (m, 1H), 3.75–3.59 (m, 2H), 2.18 (s, 3H); ^13^C NMR (126 MHz, CDCl_3_) δ 167.82, 166.18, 137.88, 136.47, 134.68, 133.54, 131.37, 131.03, 129.37, 125.53, 124.99, 123.84, 120.64, 92.62, 55.97, 33.95, 19.22; HRMS(ESI): *m*/*z* [M + H]^+^calcd. for C_24_H_20_IN_2_O_3_S^+^: 543.0234, found: 543.0233.

*(S)-N-(3-(3-Nitrophenyl)-2-phthalimidopropionyl)-2-methylthioaniline* (**3p**). **3p** was obtained as a solid. ^1^H NMR (400 MHz, CDCl_3_) δ 8.93 (s, 1H), 8.32 (d, *J* = 8.2 Hz, 1H), 8.08 (s, 1H), 8.03 (d, *J* = 8.2 Hz, 1H), 7.83 (dt, *J* = 7.0, 3.5 Hz, 2H), 7.79–7.71 (m, 2H), 7.60 (d, *J* = 7.7 Hz, 1H), 7.42 (dd, *J* = 16.3, 8.1 Hz, 2H), 7.31 (t, *J* = 7.8 Hz, 1H), 7.08 (td, *J* = 7.6, 1.1 Hz, 1H), 5.32 (dd, *J* = 10.8, 5.6 Hz, 1H), 3.83 (ddd, *J* = 25.2, 14.4, 8.2 Hz, 2H), 2.18 (s, 3H); ^13^C NMR (126 MHz, CDCl_3_) δ 167.70, 165.71, 148.45, 139.13, 137.79, 135.30, 134.88, 133.57, 131.24, 129.82, 129.43, 125.59, 125.13, 124.07, 123.95, 122.36, 120.64, 55.74, 34.07, 19.23; HRMS(ESI): *m*/*z* [M + H]^+^calcd. for C_24_H_20_N_3_O_5_S^+^: 462.1118, found: 462.1161.

*(S)-N-(3-(4-tert-Butoxycarbonylaminophenyl)-2-phthalimidopropionyl)-2-methylthioaniline* (**3q**). **3q** was obtained as a solid. ^1^H NMR (400 MHz, CDCl_3_) δ 8.96 (s, 1H), 8.33 (d, *J* = 8.2 Hz, 1H), 7.81 (dt, *J* = 7.1, 3.6 Hz, 2H), 7.75–7.68 (m, 2H), 7.44 (d, *J* = 7.7 Hz, 1H), 7.30 (t, *J* = 7.8 Hz, 1H), 7.16 (dd, *J* = 25.0, 8.4 Hz, 4H), 7.06 (td, *J* = 7.7, 1.2 Hz, 1H), 6.37 (s, 1H), 5.27 (t, *J* = 8.4 Hz, 1H), 3.65 (t, *J* = 11.5 Hz, 2H), 2.20 (s, 3H), 1.47 (s, 9H); ^13^C NMR (126 MHz, CDCl_3_) δ 167.90, 166.53, 152.72, 138.07, 137.33, 134.53, 133.57, 131.50, 131.11, 129.58, 129.35, 125.58, 124.90, 123.76, 120.70, 118.69, 80.55, 56.45, 33.76, 28.43, 19.22; HRMS(ESI): *m*/*z* [M + H]^+^calcd. for C_29_H_30_N_3_O_5_S^+^: 532.1901, found: 532.1905.

*(S)-N-(3-(4-Bromo-3-formylphenyl)-2-phthalimidopropionyl)-2-methylthioaniline* (**3r**). **3r** was obtained as an oil. ^1^H NMR (400 MHz, CDCl_3_) δ 10.22 (s, 1H), 8.91 (s, 1H), 8.31 (t, *J* = 8.1 Hz, 1H), 7.87–7.81 (m, 2H), 7.78–7.72 (m, 3H), 7.50 (d, *J* = 8.2 Hz, 1H), 7.43 (d, *J* = 7.8 Hz, 1H), 7.35 (dd, *J* = 8.2, 2.3 Hz, 1H), 7.30 (t, *J* = 7.8 Hz, 1H), 7.07 (td, *J* = 7.6, 1.2 Hz, 1H), 5.27 (dd, *J* = 10.6, 5.8 Hz, 1H), 3.73 (qd, *J* = 14.1, 8.3 Hz, 2H), 2.18 (s, 3H); ^13^C NMR (126 MHz, CDCl_3_) δ 191.51, 167.71, 165.80, 137.79, 137.40, 136.01, 134.80, 134.37, 133.58, 133.54, 131.28, 130.39, 129.38, 125.68, 125.59, 125.08, 123.94, 120.65, 55.69, 33.66, 19.23; HRMS(ESI): *m*/*z* [M + Na]^+^calcd. for C_25_H_19_BrN_2_O_4_SNa^+^: 545.0141, found: 545.0183.

*(S)-N-(3-(3-Bromo-4-formylphenyl)-2-phthalimidopropionyl)-2-methylthioaniline* (**3s**). **3s** was obtained as an oil. ^1^H NMR (400 MHz, CDCl_3_) δ 10.22 (s, 1H), 8.91 (s, 1H), 8.30 (d, *J* = 8.1 Hz, 1H), 7.84 (dt, *J* = 7.0, 3.5 Hz, 2H), 7.76 (dt, *J* = 7.8, 3.9 Hz, 3H), 7.53 (d, *J* = 5.9 Hz, 1H), 7.43 (d, *J* = 7.7 Hz, 1H), 7.35–7.27 (m, 2H), 7.07 (td, *J* = 7.6, 1.2 Hz, 1H), 5.31 (dd, *J* = 10.8, 5.6 Hz, 1H), 3.95–3.56 (m, 2H), 2.17 (s, 3H); ^13^C NMR (126 MHz, CDCl_3_) δ 191.50, 167.68, 165.64, 145.60, 137.74, 134.89, 134.47, 133.53, 132.42, 131.22, 130.21, 129.41, 128.65, 127.41, 125.61, 125.14, 123.97, 120.63, 55.39, 34.15, 19.21; HRMS(ESI): *m*/*z* [M + H]^+^calcd for C_25_H_22_BrN_2_O_4_S^+^: 525.0478, found: 525.0377.

*(S)-N-(3-(4-Bromo-2-methoxypheny)-2-phthalimidopropionyl)-2-methylthioaniline* (**3t**). **3t** was obtained as an oil. ^1^H NMR (400 MHz, CDCl_3_) δ 8.92 (s, 1H), 8.35 (d, *J* = 8.2 Hz, 1H), 7.85–7.79 (m, 2H), 7.78–7.70 (m, 2H), 7.45 (d, *J* = 7.7 Hz, 1H), 7.34–7.27 (m, 1H), 7.06 (td, *J* = 7.6, 1.2 Hz, 1H), 6.93 (d, *J* = 8.1 Hz, 2H), 6.85 (dd, *J* = 7.9, 1.7 Hz, 1H), 5.44 (dd, *J* = 10.8, 5.0 Hz, 1H), 3.80 (s, 3H), 3.64 (ddd, *J* = 24.6, 13.8, 7.9 Hz, 2H), 2.22 (s, 3H); ^13^C NMR (126 MHz, CDCl_3_) δ 167.88, 166.72, 158.38, 138.18, 134.47, 133.60, 132.17, 131.59, 129.42, 125.30, 124.78, 124.32, 123.66, 121.75, 120.55, 114.13, 55.76, 54.05, 29.94, 19.24; HRMS(ESI): *m*/*z* [M + H]^+^calcd. for C_25_H_22_BrN_2_O_4_S^+^: 525.0478, found: 525.0515.

*(S)-N-(3-(5-Formyl-2-methoxyphenyl)-2-phthalimidopropionyl)-2-methylthioaniline* (**3u**). **3u** was obtained as an oil. ^1^H NMR (400 MHz, CDCl_3_) δ 9.68 (s, 1H), 8.95 (s, 1H), 8.35 (d, *J* = 8.2 Hz, 1H), 7.82–7.77 (m, 2H), 7.74–7.68 (m, 3H), 7.58 (d, *J* = 2.0 Hz, 1H), 7.45 (d, *J* = 7.7 Hz, 1H), 7.30 (t, *J* = 7.8 Hz, 1H), 7.06 (td, *J* = 7.6, 1.1 Hz, 1H), 6.92 (d, *J* = 8.5 Hz, 1H), 5.48 (dd, *J* = 10.9, 4.8 Hz, 1H), 3.90 (s, 3H), 3.87–3.76 (m, 1H), 3.66 (dd, *J* = 13.8, 11.0 Hz, 1H), 2.21 (s, 3H); ^13^C NMR (126 MHz, CDCl_3_) δ 190.72, 167.80, 166.55, 162.79, 138.08, 134.55, 133.55, 132.86, 131.44, 129.70, 129.39, 129.29, 126.26, 125.36, 124.83, 123.65, 120.56, 110.60, 56.02, 53.90, 30.21, 19.21; HRMS(ESI): *m*/*z* [M + H]^+^calcd. for C_26_H_23_N_2_O_5_S^+^: 475.1322, found: 475.1361.

*(S)-N-(3-(2-(Benzyloxy)-5-formylphenyl)-2-phthalimidopropionyl)-2-methylthioaniline* (**3v**). **3v** was obtained as an oil. ^1^H NMR (400 MHz, CDCl_3_) δ 9.68 (s, 1H), 8.86 (s, 1H), 8.32 (d, *J* = 8.1 Hz, 1H), 7.76 (dt, *J* = 7.0, 3.6 Hz, 2H), 7.69 (tt, *J* = 5.6, 3.0 Hz, 3H), 7.60 (d, *J* = 2.0 Hz, 1H), 7.50 (d, *J* = 7.3 Hz, 2H), 7.46–7.36 (m, 3H), 7.31 (dd, *J* = 16.6, 7.7 Hz, 2H), 7.05 (td, *J* = 7.6, 1.3 Hz, 1H), 6.99 (d, *J* = 8.5 Hz, 1H), 5.55 (dd, *J* = 10.9, 4.8 Hz, 1H), 5.30–5.14 (m, 2H), 3.88 (dd, *J* = 13.8, 4.8 Hz, 1H), 3.71 (dd, *J* = 13.8, 11.0 Hz, 1H), 2.15 (s, 3H); ^13^C NMR (126 MHz, CDCl_3_) δ 190.65, 167.80, 166.40, 161.94, 138.02, 135.87, 134.49, 133.50, 133.02, 131.42, 131.26, 129.85, 129.32, 128.92, 128.42, 127.47, 126.47, 125.47, 124.82, 123.64, 120.62, 111.94, 70.85, 53.75, 30.38, 19.11;HRMS(ESI): *m*/*z* [M + H]^+^calcd. for C_32_H_27_N_2_O_5_S^+^: 551.1635, found: 551.1676.

### 3.3. Synthesis of the Compounds 3a and 1

*Methyl (S)-3-(4-methoxy-3-formylphenyl)-2-phthalimidopropionate* (**2a**). **3a** (3.0 g, 6.3 mmol), dry methanol (600 mL), conc. H_2_SO_4_ (5.0 mL) were added to a 500-mL round bottom flask. The mixture was stirred at 100 °C for 24 h under N_2_. After cooling to room temperature, the resulting solution was evaporated, and the residue was diluted with ethyl acetate (200 mL) and brine (100 mL).The organic layer was separated and washed successively with water, saturated aqueous NaHCO_3_ and brine, dried over anhydrous Na_2_SO_4_, and concentrated in vacuo. The organic layer was purified via column chromatography in a petroleum ether/ethyl acetate mixture of2:1 to produce corresponding product**2a** (1.8 g, 77%) as a solid.^1^H NMR (500 MHz, CDCl_3_) δ 10.31 (s, 1H), 7.84–7.75 (m, 2H), 7.74–7.65 (m, 2H), 7.60 (d, *J* = 2.4 Hz, 1H), 7.37 (dd, *J* = 8.6, 2.4 Hz, 1H), 6.83 (d, *J* = 8.6 Hz, 1H), 5.08 (dd, *J* = 11.1, 5.1 Hz, 1H), 3.84 (s, 3H), 3.77 (s, 3H), 3.60–3.42 (m, 2H); ^13^C NMR (126 MHz, CDCl_3_) δ 189.55, 169.24, 167.54, 160.85, 136.37, 134.35, 131.65, 129.23, 129.09, 124.79, 123.72, 112.12, 55.76, 53.32, 53.08, 33.81; HRMS(ESI): *m*/*z* [M + H]^+^calcd. for C_20_H_18_NO_6_^+^: 368.1129, found: 368.1221.

*Methyl (S)-3-(4-methoxy-3-hydroxy-phenyl)-2-phthalimidopropionate* (**1**). **2a** (750 mg, 2.0 mmol), dichloromethane (50 mL), and *meta*-chloroperoxybenzoic acid(*m*-CPBA,85% purity, 770 mg, 3.8 mmol) were added to a 250-mL round bottom flask. The mixture was stirred at room temperature overnight. The resulting solution was diluted with dichloromethane(DCM, 300 mL), washed successively with saturated aqueous Na_2_S_2_O_3_ and brine, dried over anhydrous Na_2_SO_4_, and concentrated in vacuo. The crude intermediate was used for the next step without further purification. A mixture of the concentrated residueandNaHCO_3_ (3.4 g, 40 mmol) in tetrahydrofuran (THF,40 mL) and water (20 mL) was stirred at room temperature overnight. Then, the resulting solution was evaporated, and the residue was adjusted to pH<1 with 2 M HCl. The mixture was extracted with ethyl acetate (200 mL). The organic phase was washed with brine, dried over anhydrous Na_2_SO_4_, and concentrated in vacuo. This mixture was purified via column chromatography in a petroleum ether/ethyl acetate mixture of3:1 to produce corresponding product**1** (540mg, 74% yield for two steps) as a solid.^1^H NMR (400 MHz, CDCl_3_) δ 7.85–7.73 (m, 2H), 7.74–7.60 (m, 2H), 6.72 (d, *J* = 1.5 Hz, 1H), 6.68–6.59 (m, 2H), 5.54 (s, 1H), 5.09 (dd, *J* = 11.2, 5.2 Hz, 1H), 3.76 (s, 6H), 3.46 (qd, *J* = 14.4, 8.2 Hz, 2H); ^13^C NMR (101 MHz, CDCl_3_) δ 169.51, 167.61, 145.51, 134.18, 131.73, 129.96, 123.61, 120.40, 115.19, 110.78, 55.90, 53.55, 52.97, 34.11; HRMS(ESI): *m*/*z* [M + H]^+^calcd. for C_19_H_18_NO_6_^+^: 356.1129, found: 356.1138.

### 3.4. Synthesis of the 4-Aryloxyphenylalanine Derivatives 6a–l

A Schlenk tube was charged with 1 (0.56 mmol), aryl iodide (2.0 equiv., or 3.0 equiv. used for synthesis of **6j**), CuI (1.0 equiv.), *N,N*-dimethylglycine hydrochloride salt (3.0 equiv.), Cs_2_CO_3_ (5.0 equiv.), and 5.6 mL of 1,4-dioxane, evacuated, and backfilled with N_2_. The reaction mixture was stirred at 90 °C for 20 h. The suspension was filtered, and the filtrate was concentrated, followed by column chromatography on silica gel (eluting with 1:10 to 1:5 ethyl acetate/petroleum ether) to provide the desired product.

*(S)-3-(3-{4-[(R)-2-Benzyloxycarbonyl-2-tert-butoxycarbonylamino]ethyl}phenoxy)-4-methoxyphenyl)-2-phthalimido-propionic acid methyl ester* (**6a**). **6a** was obtained as an oil. ^1^H NMR (400 MHz, CDCl_3_) δ 7.77 (dt, *J* = 5.3, 2.7 Hz, 2H), 7.73–7.65 (m, 2H), 7.40–7.28 (m, 5H), 6.92 (d, *J* = 8.3 Hz, 1H), 6.81 (d, *J* = 8.3 Hz, 3H), 6.73 (s, 1H), 6.60 (d, *J* = 7.6 Hz, 2H), 5.14 (q, *J* = 12.3 Hz, 2H), 5.07–5.01 (m, 1H), 4.99 (d, *J* = 3.9 Hz, 1H), 4.64–4.51 (m, 1H), 3.75 (s, 3H), 3.72 (s, 3H), 3.53–3.35 (m, 2H), 3.01 (d, *J* = 5.7 Hz, 2H), 1.42 (s, 9H); ^13^C NMR (126 MHz, CDCl_3_) δ 171.88, 169.30, 167.46, 156.96, 155.27, 150.35, 144.75, 135.34, 134.30, 131.66, 130.46, 129.74, 129.68, 128.75, 128.66, 125.28, 123.68, 121.83, 121.75, 117.06, 112.97, 80.11, 67.25, 56.01, 54.63, 53.49, 53.02, 37.56, 33.94, 28.45; HRMS(ESI): *m*/*z* [M + Na]^+^calcd. for C_40_H_40_N_2_O_10_Na^+^: 731.2575, found: 731.2584.

*(S)-3-(3-{4-[(S)-2-Benzyloxycarbonyl-2-tert-butoxycarbonylamino]ethyl}phenoxy)-4-methoxyphenyl)-2-phthalimido-propionic acid methyl ester* (**6b**). **6b** was obtained as an oil. ^1^H NMR (400 MHz, CDCl_3_) δ 7.77 (dt, *J* = 5.3, 2.7 Hz, 2H), 7.74–7.67 (m, 2H), 7.40–7.28 (m, 5H), 6.92 (d, *J* = 8.3 Hz, 1H), 6.88–6.78 (m, 3H), 6.72 (s, 1H), 6.59 (d, *J* = 7.5 Hz, 2H), 5.23–5.07 (m, 2H), 5.07–4.94 (m, 2H), 4.65–4.49 (m, 1H), 3.75 (s, 3H), 3.72 (s, 3H), 3.55–3.33 (m, 2H), 3.00 (d, *J* = 5.7 Hz, 2H), 1.42 (s, 9H); ^13^C NMR (126 MHz, CDCl_3_) δ 171.86, 169.30, 167.46, 156.95, 155.27, 150.35, 144.69, 135.34, 134.30, 131.66, 130.46, 129.74, 129.69, 128.74, 128.66, 128.63, 125.27, 123.68, 121.82, 121.74, 117.02, 112.97, 80.07, 67.24, 56.01, 54.60, 53.46, 53.01, 37.54, 33.94, 28.44; HRMS(ESI): *m*/*z* [M + Na]^+^calcd. for C_40_H_40_N_2_O_10_Na^+^: 731.2575, found: 731.2577.

*(S)-3-(3-{4-[(R)-2-(2-trimethylsilylethoxycarbonyl)-2-tert-butoxycarbonylamino]ethyl}phenoxy)-4-methoxyphenyl)-2-phthalimido-propionic Acid Methyl Ester* (**6c**). **6c** was obtained as an oil. ^1^H NMR (400 MHz, CDCl_3_) δ 7.78 (dt, *J* = 7.6, 2.9 Hz, 2H), 7.75–7.69 (m, 2H), 6.91 (dd, *J* = 8.3, 1.8 Hz, 3H), 6.80 (d, *J* = 8.4 Hz, 1H), 6.73 (s, 1H), 6.65 (d, *J* = 7.1 Hz, 2H), 5.04 (dd, *J* = 11.4, 5.1 Hz, 1H), 4.97 (d, *J* = 7.9 Hz, 1H), 4.49 (d, *J* = 5.4 Hz, 1H), 4.27–4.10 (m, 2H), 3.75 (s, 3H), 3.72 (d, *J* = 1.4 Hz, 3H), 3.45 (ddd, *J* = 25.8, 14.4, 8.3 Hz, 2H), 3.11–2.87 (m, 2H), 1.42 (s, 9H), 0.98 (dd, *J* = 9.7, 7.6 Hz, 2H), 0.04 (d, *J* = 0.7 Hz, 9H); ^13^C NMR (126 MHz, CDCl_3_) δ 172.15, 169.30, 167.46, 156.91, 155.28, 150.34, 144.78, 134.31, 131.69, 130.49, 130.08, 129.70, 125.23, 123.69, 121.76, 121.69, 117.04, 112.99, 79.97, 63.88, 56.03, 54.67, 53.49, 53.01, 37.64, 33.95, 28.46, 17.49, −1.38; HRMS(ESI): *m*/*z* [M + Na]^+^calcd. for C_38_H_46_N_2_O_10_SiNa^+^: 741.2814, found: 741.2805.

*(S)-3-(3-{4-[(R)-2-Allyloxycarbonyl-2-tert-butoxycarbonylamino]ethyl}phenoxy)-4-methoxyphenyl)-2-phthalimido-propionic acid methyl ester* (**6d**). **6d** was obtained as an oil. ^1^H NMR (400 MHz, CDCl_3_) δ 7.78 (dt, *J* = 7.5, 2.9 Hz, 2H), 7.75–7.70 (m, 2H), 6.91 (d, *J* = 8.3 Hz, 3H), 6.80 (d, *J* = 8.4 Hz, 1H), 6.73 (d, *J* = 1.3 Hz, 1H), 6.65 (dd, *J* = 8.6, 2.4 Hz, 2H), 5.87 (ddt, *J* = 16.9, 11.3, 5.8 Hz, 1H), 5.28 (dd, *J* = 22.7, 13.8 Hz, 2H), 5.04 (dd, *J* = 11.4, 5.1 Hz, 1H), 4.99 (d, *J* = 5.0 Hz, 1H), 4.60 (d, *J* = 5.8 Hz, 2H), 4.56 (s, 1H), 3.75 (s, 3H), 3.72 (s, 3H), 3.45 (dd, *J* = 16.9, 8.3 Hz, 2H), 3.02 (d, *J* = 5.3 Hz, 2H), 1.42 (s, 9H); ^13^C NMR (126 MHz, CDCl_3_) δ 171.75, 169.30, 167.46, 156.96, 155.27, 150.34, 144.76, 134.31, 131.68, 130.48, 129.89, 129.70, 125.26, 123.69, 121.76, 121.69, 119.05, 117.09, 112.99, 80.08, 66.06, 56.03, 54.64, 53.47, 53.02, 37.65, 33.95, 28.45; HRMS(ESI): *m*/*z* [M + Na]^+^calcd. for C_36_H_38_N_2_O_10_Na^+^: 681.2419, found: 681.2408.

*(S)-Methyl 2-phthalimido-3-(3-(4-formylphenoxy)-4-methoxyphenyl)-propanoate* (**6e**). **6e** was obtained as an oil. ^1^H NMR (400 MHz, CDCl_3_) δ 9.86 (s, 1H), 7.77 (dt, *J* = 6.9, 3.6 Hz, 2H), 7.74–7.68 (m, 2H), 7.68–7.62 (m, 2H), 7.04 (dd, *J* = 8.4, 2.1 Hz, 1H), 6.86 (dd, *J* = 5.3, 3.1 Hz, 2H), 6.76 (d, *J* = 8.7 Hz, 2H), 5.07 (dd, *J* = 11.4, 5.2 Hz, 1H), 3.77 (s, 3H), 3.69 (s, 3H), 3.50 (qd, *J* = 14.4, 8.3 Hz, 2H); ^13^C NMR (126 MHz, CDCl_3_) δ 190.90, 169.20, 167.51, 163.51, 150.60, 142.83, 134.41, 131.90, 131.59, 130.88, 130.05, 126.84, 123.69, 123.19, 116.20, 113.24, 55.98, 53.47, 53.09, 33.89; HRMS(ESI): *m*/*z* [M + H]^+^calcd. for C_26_H_22_NO_7_^+^: 460.1391, found: 460.1394.

*(S)-Methyl 2-phthalimido-3-(3-(3-formylphenoxy)-4-methoxyphenyl)-propanoate* (**6f**). **6f** was obtained as an oil. ^1^H NMR (400 MHz, CDCl_3_) δ 9.89 (s, 1H), 7.82–7.74 (m, 2H), 7.73–7.67 (m, 2H), 7.51 (d, *J* = 7.6 Hz, 1H), 7.34 (t, *J* = 7.8 Hz, 1H), 7.20 (dd, *J* = 2.3, 1.4 Hz, 1H), 7.08–7.02 (m, 1H), 7.00–6.94 (m, 1H), 6.83 (d, *J* = 8.6 Hz, 2H), 5.06 (dd, *J* = 11.3, 5.2 Hz, 1H), 3.76 (s, 3H), 3.71 (s, 3H), 3.56–3.40 (m, 2H); ^13^C NMR (126 MHz, CDCl_3_) δ 192.02, 169.25, 167.51, 158.89, 150.52, 143.76, 137.94, 134.34, 131.61, 130.21, 129.96, 126.24, 123.68, 122.87, 122.45, 117.03, 113.14, 55.99, 53.41, 53.06, 33.94; HRMS(ESI): *m*/*z* [M + H]^+^calcd. for C_26_H_22_NO_7_^+^: 460.1391, found: 460.1394.

*(S)-Methyl2-phthalimido-3-(3-(3-formyl-4-methoxyphenoxy)-4-methoxyphenyl)-propanoate*(**6g**). **6g** was obtained as an oil. ^1^H NMR (400 MHz, CDCl_3_) δ 10.38 (s, 1H), 7.83–7.74 (m, 2H), 7.74–7.67 (m, 2H), 7.23 (d, *J* = 3.1 Hz, 1H), 7.08 (dd, *J* = 9.0, 3.2 Hz, 1H), 6.91–6.85 (m, 2H), 6.79 (d, *J* = 8.4 Hz, 1H), 6.67 (d, *J* = 2.1 Hz, 1H), 5.04 (dd, *J* = 11.3, 5.3 Hz, 1H), 3.92 (s, 3H), 3.75 (s, 3H), 3.74 (s, 3H), 3.44 (qd, *J* = 14.4, 8.3 Hz, 2H); ^13^C NMR (126 MHz, CDCl_3_) δ 189.25, 169.32, 167.49, 157.75, 151.28, 149.93, 145.40, 134.27, 131.62, 129.69, 125.59, 125.41, 125.07, 123.67, 120.62, 116.84, 113.04, 112.90, 56.20, 56.00, 53.30, 53.00, 33.93; HRMS(ESI): *m*/*z* [M + H]^+^calcd. for C_27_H_24_NO_8_^+^: 490.1496, found: 490.1510.

*(S)-Methyl 2-phthalimido-3-(4-methoxy-3-(4-nitrophenoxy)phenyl)-propanoate* (**6h**). **6h** was obtained as an oil. ^1^H NMR (400 MHz, CDCl_3_) δ 8.04–7.95 (m, 2H), 7.80–7.75 (m, 2H), 7.75–7.70 (m, 2H), 7.07 (dd, *J* = 8.4, 2.1 Hz, 1H), 6.87 (d, *J* = 8.7 Hz, 2H), 6.73–6.65 (m, 2H), 5.07 (dd, *J* = 11.4, 5.2 Hz, 1H), 3.77 (s, 3H), 3.69 (s, 3H), 3.51 (qd, *J* = 14.4, 8.3 Hz, 2H); ^13^C NMR (126 MHz, CDCl_3_) δ 169.13, 167.49, 163.59, 150.51, 142.43, 142.26, 134.47, 131.54, 130.17, 127.29, 125.83, 123.70, 123.28, 115.76, 113.30, 55.94, 53.45, 53.12, 33.87; HRMS(ESI): *m*/*z* [M + H]^+^calcd. for C_25_H_21_N_2_O_8_^+^: 477.1292, found: 477.1304.

*(S)-Methyl 2-phthalimido-3-(3-(4-cyanophenoxy)-4-methoxyphenyl)-propanoate* (**6i**). **6i** was obtained as an oil. ^1^H NMR (400 MHz, CDCl_3_) δ 7.81–7.69 (m, 4H), 7.44–7.35 (m, 2H), 7.03 (dd, *J* = 8.4, 2.1 Hz, 1H), 6.85 (t, *J* = 4.9 Hz, 2H), 6.75–6.64 (m, 2H), 5.06 (dd, *J* = 11.4, 5.2 Hz, 1H), 3.77 (s, 3H), 3.69 (s, 3H), 3.49 (qd, *J* = 14.4, 8.3 Hz, 2H); ^13^C NMR (126 MHz, CDCl_3_) δ 169.14, 167.48, 161.89, 150.56, 142.51, 134.42, 133.97, 131.58, 130.12, 127.04, 123.68, 123.19, 119.12, 116.56, 113.25, 105.20, 55.95, 53.46, 53.10, 33.86; HRMS(ESI): *m*/*z* [M + H]^+^calcd. for C_26_H_21_N_2_O_6_^+^: 457.1394, found: 457.1401.

*(S)-Methyl 2-phthalimido-3-(3-(4-iodophenoxy)-4-methoxyphenyl)-propanoate* (**6j**). **6j** was obtained as an oil. ^1^H NMR (400 MHz, CDCl_3_) δ 7.83–7.68 (m, 4H), 7.40–7.32 (m, 2H), 6.96 (dd, *J* = 8.4, 2.1 Hz, 1H), 6.85–6.78 (m, 1H), 6.73 (d, *J* = 2.1 Hz, 1H), 6.49–6.41 (m, 2H), 5.04 (dd, *J* = 11.4, 5.2 Hz, 1H), 3.76 (s, 3H), 3.72 (s, 3H), 3.55–3.36 (m, 2H); ^13^C NMR (126 MHz, CDCl_3_) δ 169.23, 167.48, 158.04, 150.39, 144.11, 138.34, 134.37, 131.60, 129.87, 125.86, 123.68, 122.07, 119.04, 113.10, 84.92, 56.02, 53.55, 53.06, 33.89; HRMS(ESI): *m*/*z* [M + H]^+^calcd. for C_25_H_21_INO_6_^+^: 558.0408, found: 558.0411.

*(S)-Methyl 2-phthalimido-3-(3-(4-acetylphenoxy)-4-methoxyphenyl)-propanoate* (**6k**). **6k** was obtained as an oil. ^1^H NMR (400 MHz, CDCl_3_) δ 7.81–7.75 (m, 3H), 7.71 (tt, *J* = 8.8, 2.5 Hz, 3H), 7.01 (dd, *J* = 8.4, 2.1 Hz, 1H), 6.85 (d, *J* = 8.1 Hz, 2H), 6.73–6.67 (m, 2H), 5.10–5.03 (m, 1H), 3.77 (s, 3H), 3.70 (s, 3H), 3.57–3.39 (m, 2H), 2.54 (s, 3H); ^13^C NMR (126 MHz, CDCl_3_) δ 196.85, 169.22, 167.50, 162.31, 150.58, 143.11, 134.39, 131.57, 131.38, 130.54, 129.95, 126.55, 123.69, 123.00, 115.75, 113.18, 55.98, 53.47, 53.08, 33.90, 26.58; HRMS(ESI): *m*/*z* [M + H]^+^calcd. for C_27_H_24_NO_7_^+^: 474.1547, found: 474.1573.

*(S)-Methyl 2-phthalimido-3-(3-(4-methoxycarbonyl-3-benzyloxyphenoxy)-4-methoxyphenyl)-propanoate* (**6l**). **6l** was obtained as an oil. ^1^H NMR (400 MHz, CDCl_3_) δ 7.75 (dd, *J* = 5.5, 3.0 Hz, 2H), 7.67 (dd, *J* = 5.4, 3.1 Hz, 2H), 7.62 (d, *J* = 8.7 Hz, 1H), 7.45 (d, *J* = 7.3 Hz, 2H), 7.36 (t, *J* = 7.4 Hz, 2H), 7.30 (d, *J* = 7.3 Hz, 1H), 6.99 (dd, *J* = 8.4, 2.1 Hz, 1H), 6.82 (dd, *J* = 8.3, 5.3 Hz, 2H), 6.49 (d, *J* = 2.3 Hz, 1H), 6.15 (dd, *J* = 8.7, 2.3 Hz, 1H), 5.08 (s, 2H), 5.07–5.01 (m, 1H), 3.88 (s, 3H), 3.77 (s, 3H), 3.64 (s, 3H), 3.55–3.39 (m, 2H); ^13^C NMR (126 MHz, CDCl_3_) δ 169.21, 167.51, 166.29, 162.81, 160.25, 150.56, 143.14, 136.75, 134.38, 133.72, 131.56, 129.97, 128.64, 127.84, 126.95, 126.49, 123.67, 122.94, 113.97, 113.07, 107.60, 102.16, 70.47, 55.91, 53.54, 53.07, 51.89, 33.93; HRMS(ESI): *m*/*z* [M + H]^+^calcd. for C_34_H_30_NO_9_^+^: 596.1915, found: 596.1930.

## Figures and Tables

**Table 1:** Palladium-catalyzed arylation of **4a**–**c** with **5a**.

Entry	Substrate	Temp.	Time	Yield (%)	Ratio (mono/di) ^g^
1 ^a^	**4a**	150 °C	24 h	38 ^e^	2.2:1 ^h^
2 ^a^	**4b**	150 °C	24 h	12 ^e^	1:3.1 ^h^
3 ^b^	**4a**	100 °C	48 h	48 ^e^	12:1
4 ^b^	**4a**	80 °C	48 h	62 ^e^	mono
5 ^b^	**4a**	60 °C	60 h	42 ^f^	mono
6 ^c^	**4b**	80 °C	48 h	26 ^f^	1:1.7
7 ^d^	**4c**	80 °C	48 h	36 ^e^	mono
8 ^i^	**4a**	80 °C	48 h	65 ^e^	–
9 ^j^	**4a**	80 °C	48 h	51 ^e^	–

^a^ **4** (0.6 mmol) was used. ^b^ **4a** (1.5 mmol) was used. ^c^ **4b** (1.5 mmol) was used. ^d^ **4c** (4.0 mmol) was used. ^e^ Isolated yields. ^f^ Yields are based on ^1^H-NMR analysis. ^g^ The ratio of the mono- to the diarylated product was determined via ^1^HNMR spectroscopy of the crude reaction mixture. ^h^ The ratio of the mono- to the diarylated product was determined via isolated yield. ^i^ **4a** (4.4 mmol) was used. ^j^ **4a** (18.5 mmol) was used.

## Data Availability

The original data presented in the study are included in the article/Supplementary Materials; further inquiries can be directed to the corresponding author.

## Supplementary Materials

The following supporting information can be downloaded at: https://www.mdpi.com/article/10.3390/md21070373/s1, Copies of NMR spectra.
