# Supplementary material for: Synthesis of the Isodityrosine Moiety of Seongsanamide A–D and Its Derivatives
Source: Mar Drugs. 2023 Jun 25;21(7):373. doi: 10.3390/md21070373 (PMC10381827; doi:10.3390/md21070373)

Supplementary Material

Synthesis of isodityrosine moiety of seongsanamide A-D and its derivatives

Yang Xie[1], Zhou Xu[2], Pei Hu[2], Xiao-Ting Tian[2], Yi-Hong Lu[2], Hao-Dong Jiang[2], Cheng-Gang Huang[2,*] and Zhi-Cai Shang[1,*]

[1]Department of Chemistry, Zhejiang University, Hangzhou 310027, China

[2]Shanghai Institute of Materia Medica, Chinese Academy of Sciences, Shanghai 201203, China

Table of contents

1. Copies of ^{1}H and ^{13}C NMR spectra of compounds described

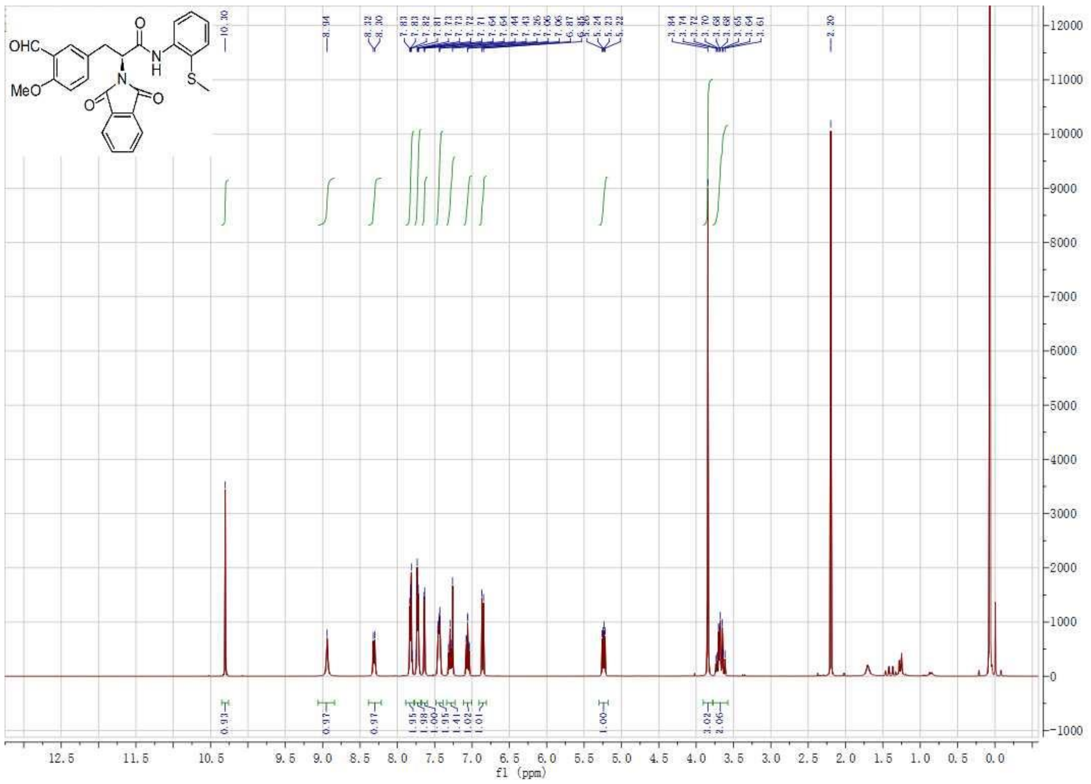
OHC
MeO
f1 (ppm)

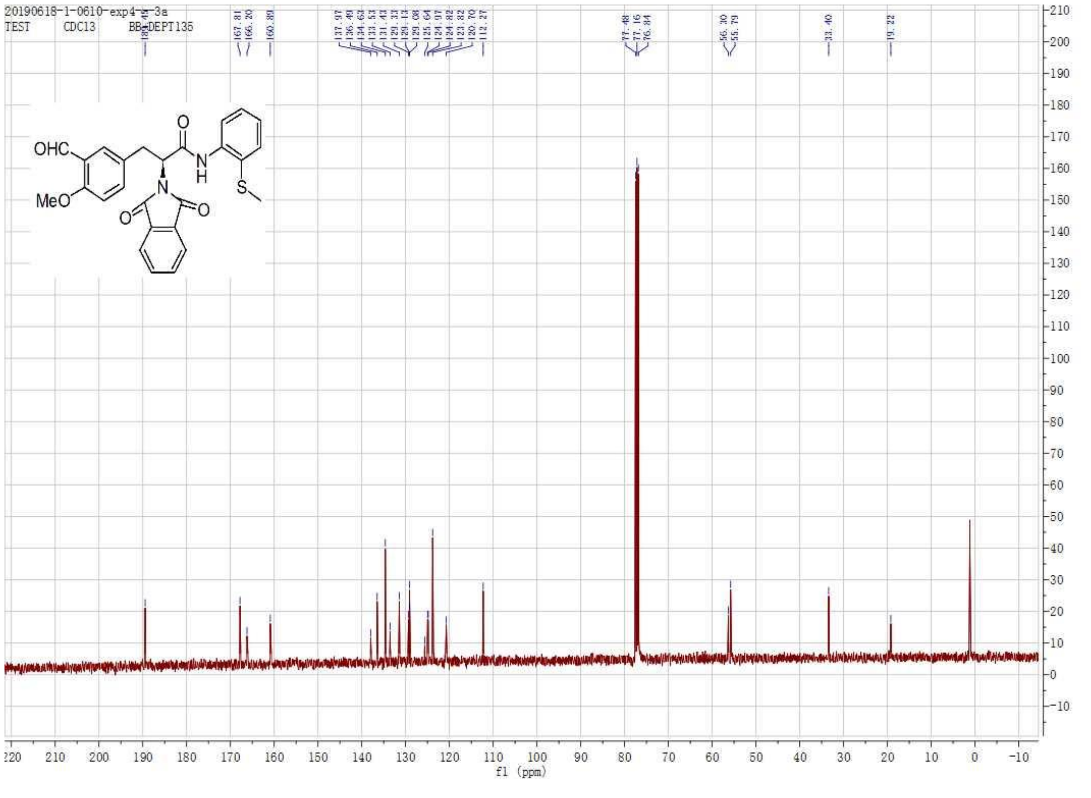
20190618-1-0610-exp4-a-3a
TEST CDCl3 BB-DEPT135
OHC
MeO
f1 (ppm)

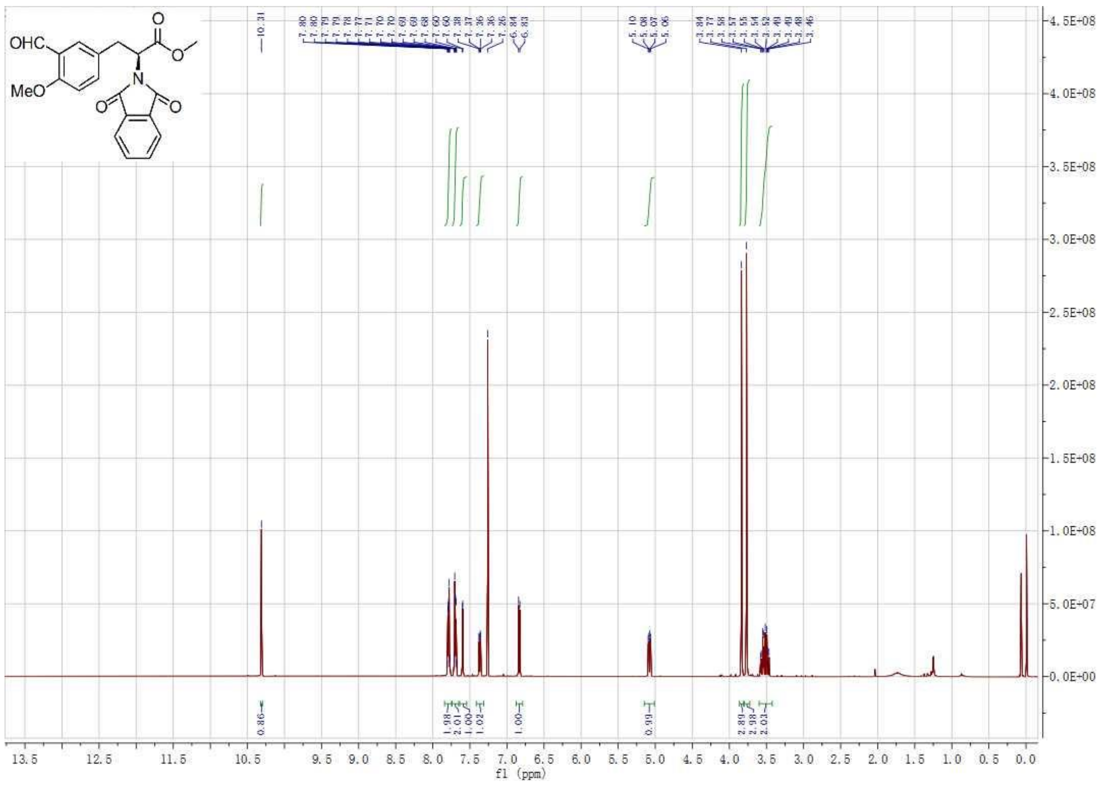

OHC
MeO
O
N
10.31
f1 (ppm)

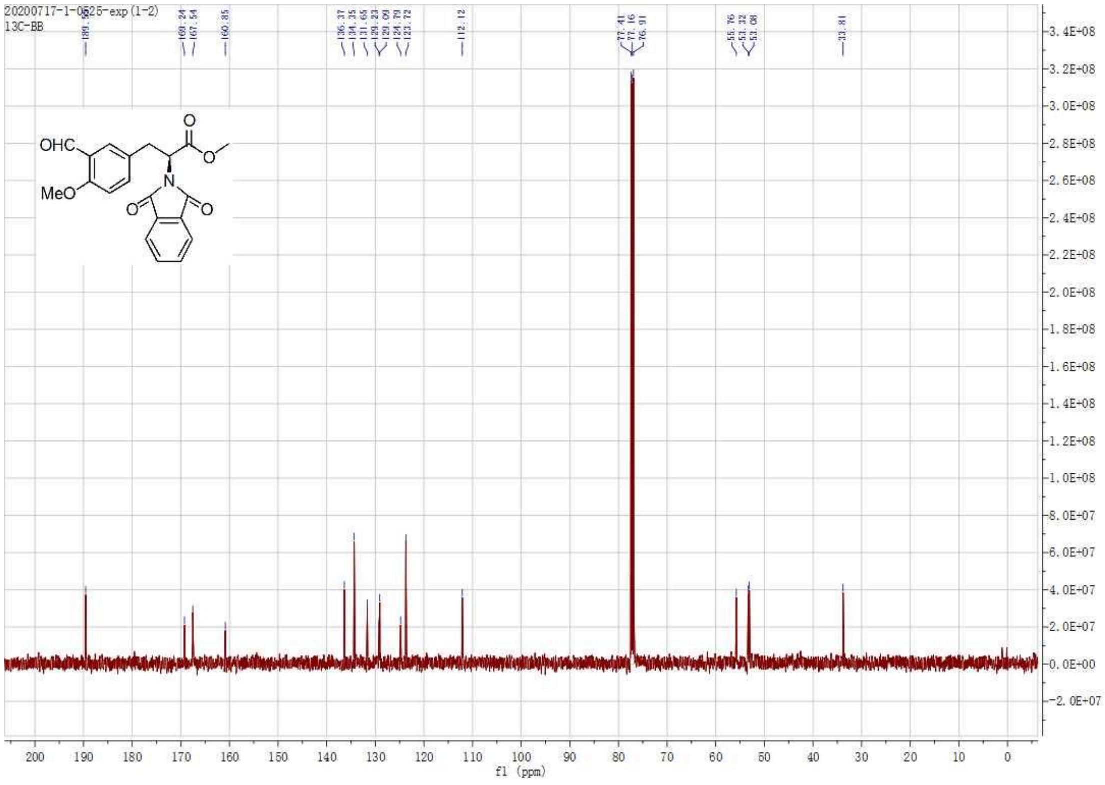

20200717-1-0525-exp(1-2)
13C-BB
OHC
MeO
O
N
f1 (ppm)

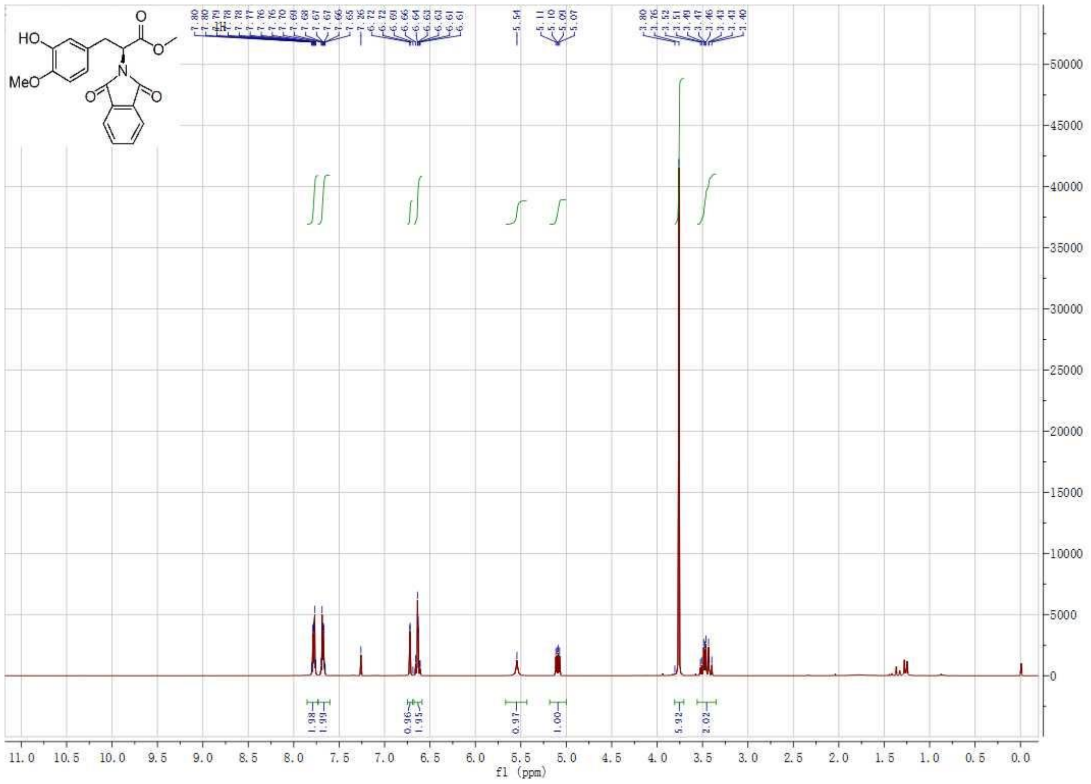

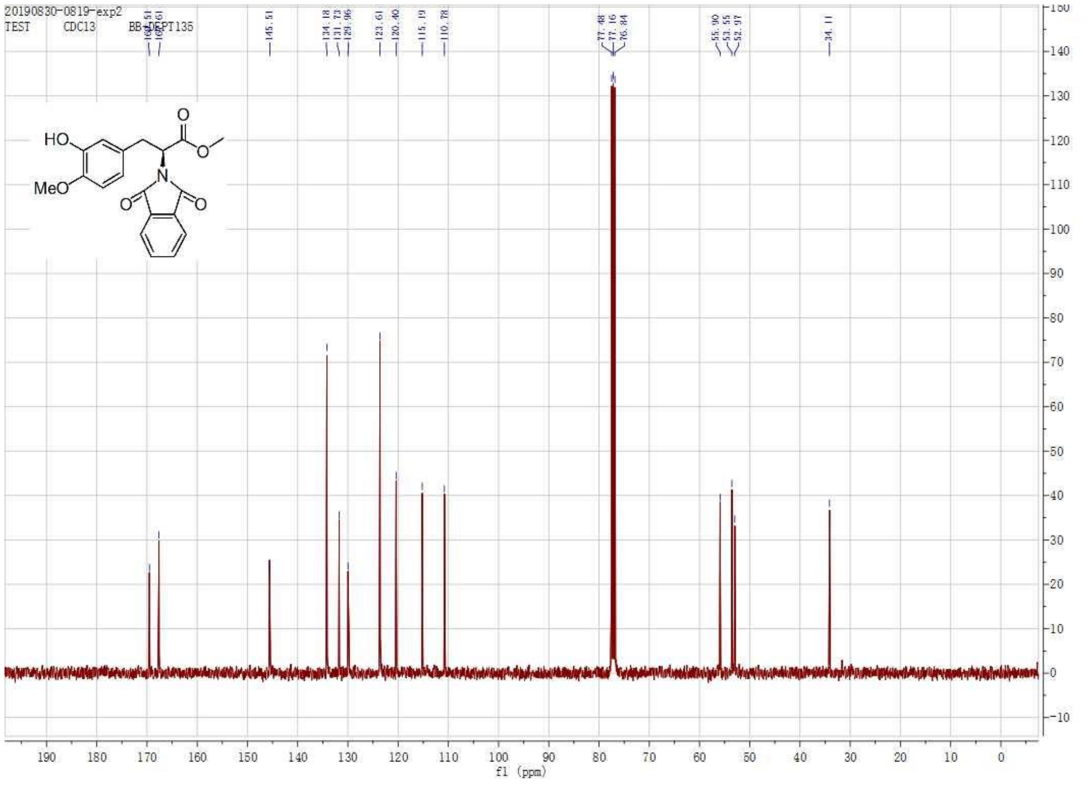

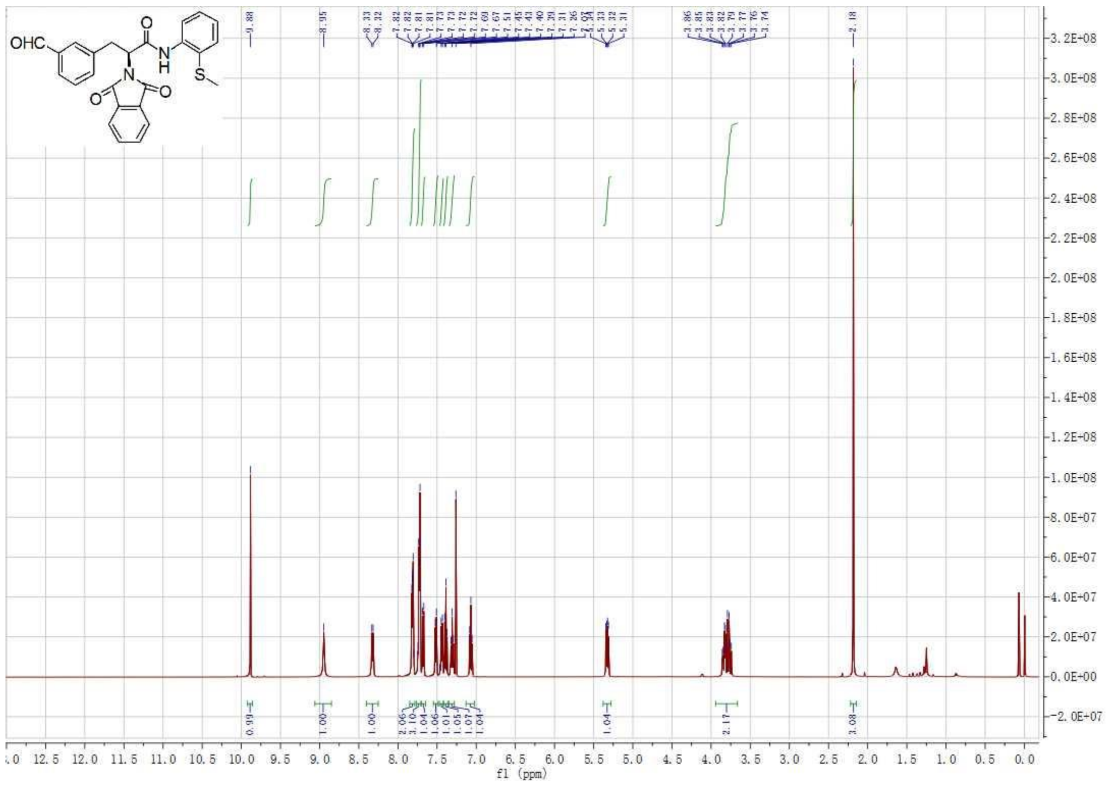

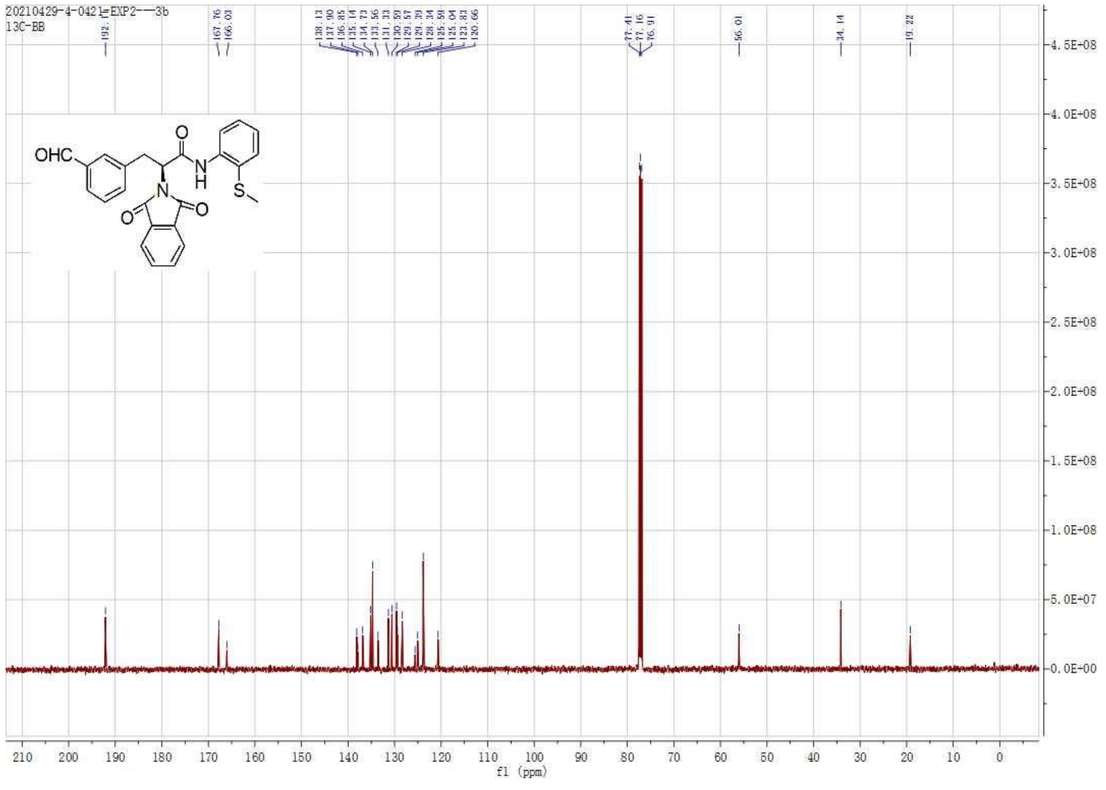

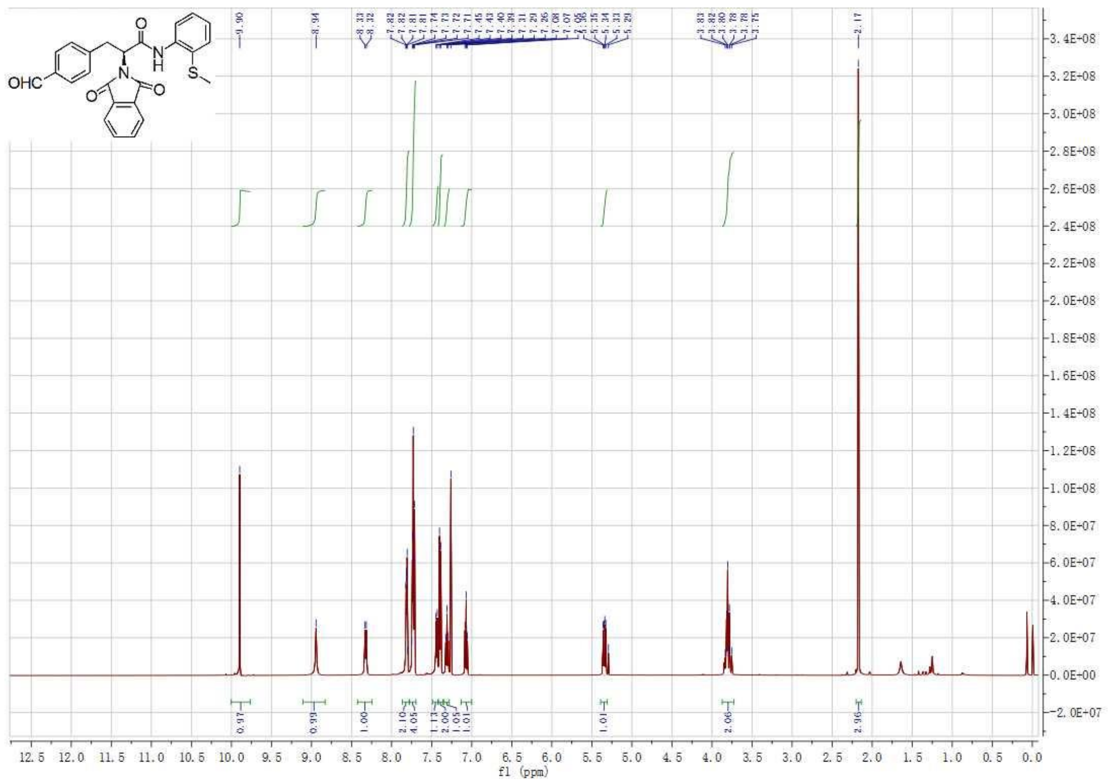
OHC
O
N
H
S
9.90
8.94
8.33
8.32
5.35
5.34
5.33
5.29
3.83
3.82
3.80
3.78
3.75
2.17
0.97
0.99
1.00
2.10
4.05
1.13
2.00
1.05
1.01
1.01
2.06
2.96
f1 (ppm)

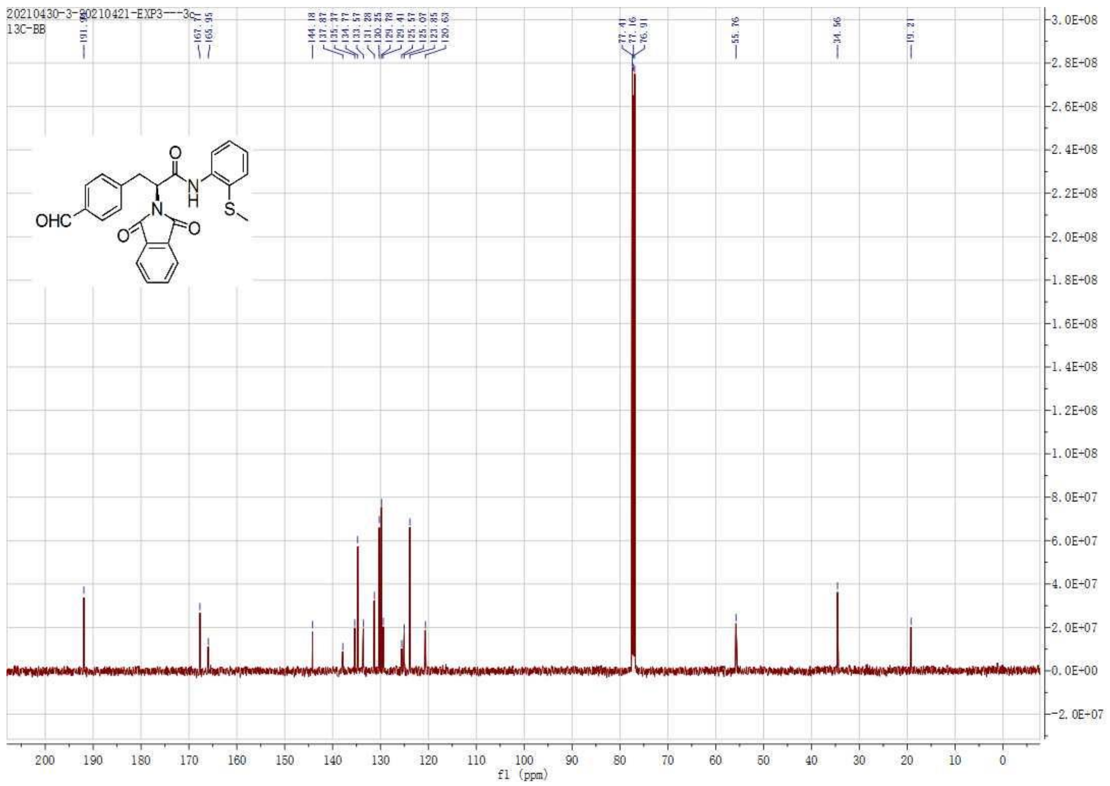
13C-BB
165.95
144.18
137.87
135.37
134.77
133.57
131.28
130.25
129.78
129.41
125.57
123.85
120.63
77.41
77.16
76.91
55.76
34.56
19.21
OHC
O
N
H
S
f1 (ppm)

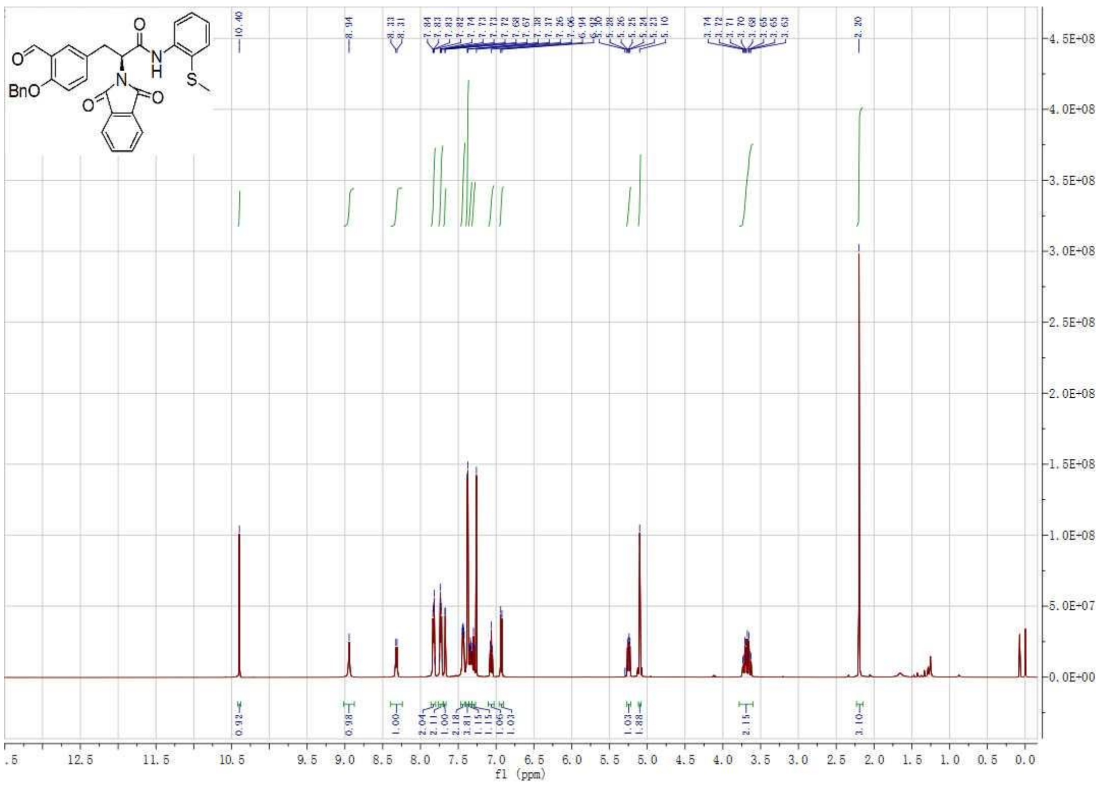
BnO
fl (ppm)

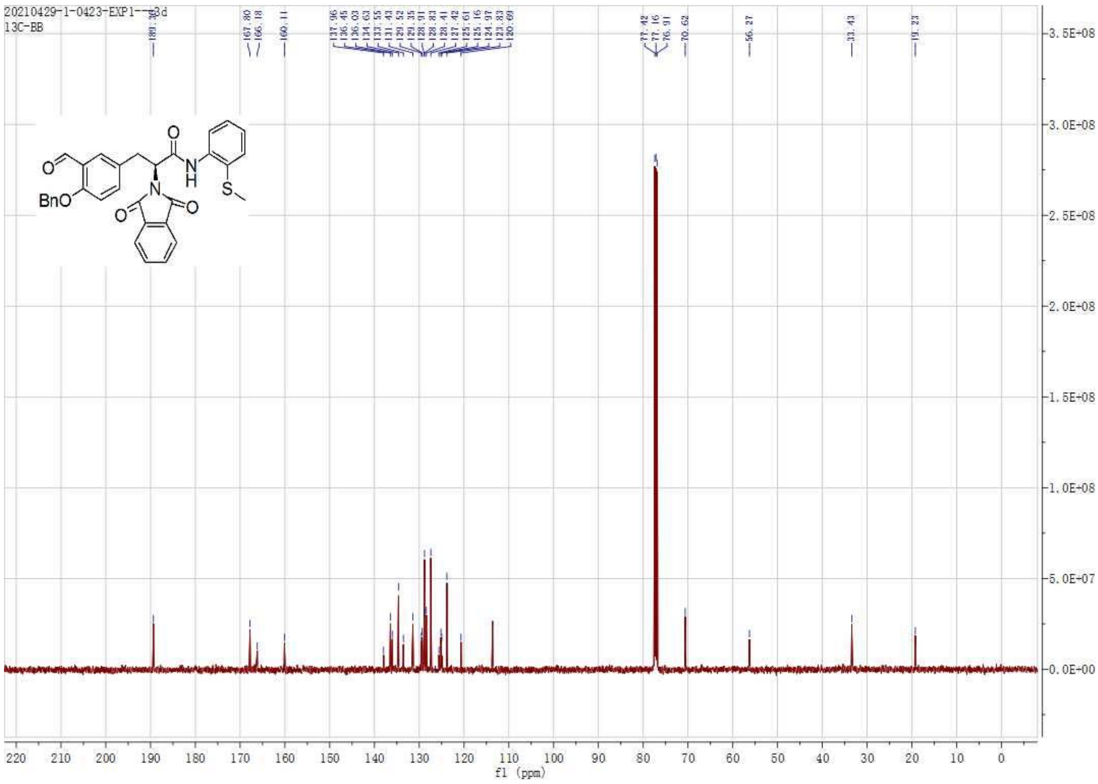
20210429-1-0423-EXP1--m3d
13C-BB
BnO
fl (ppm)

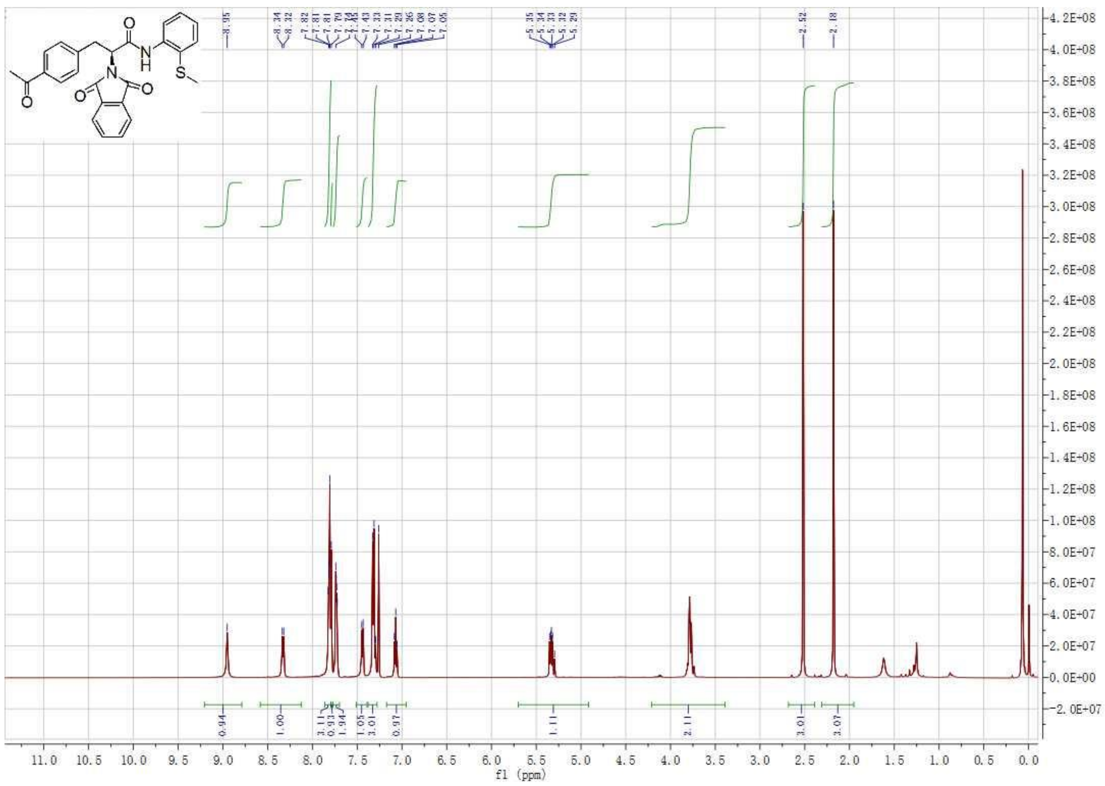

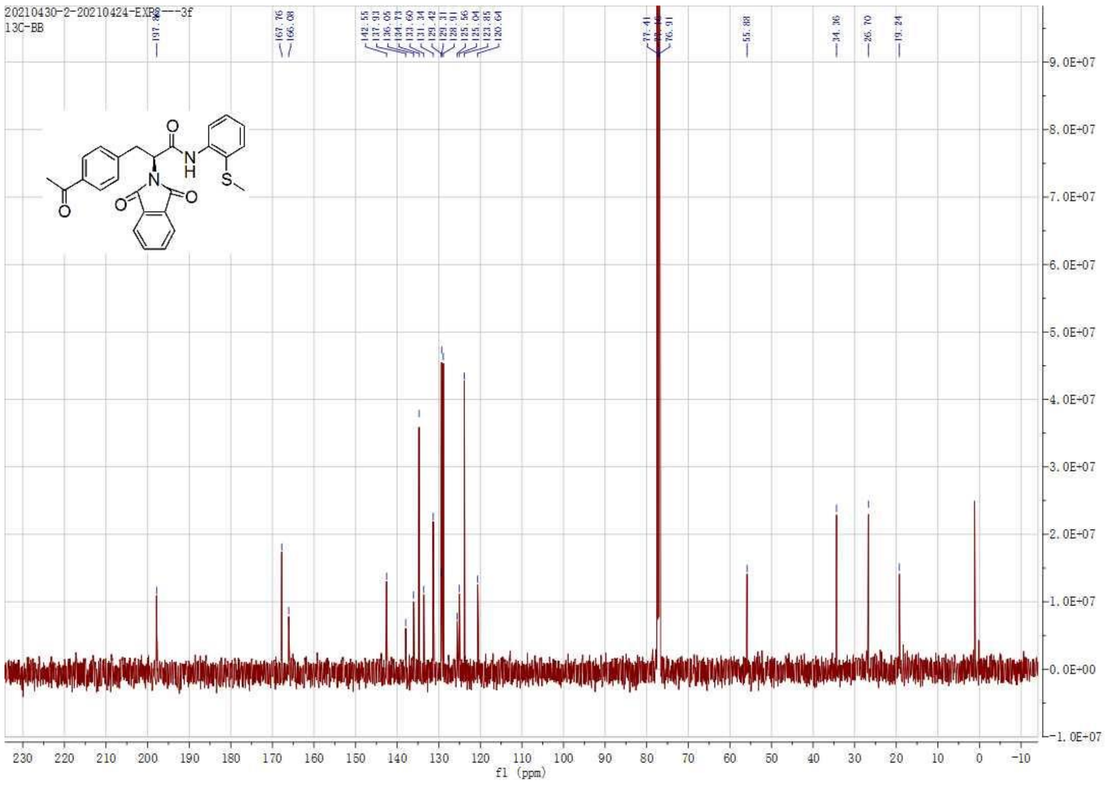

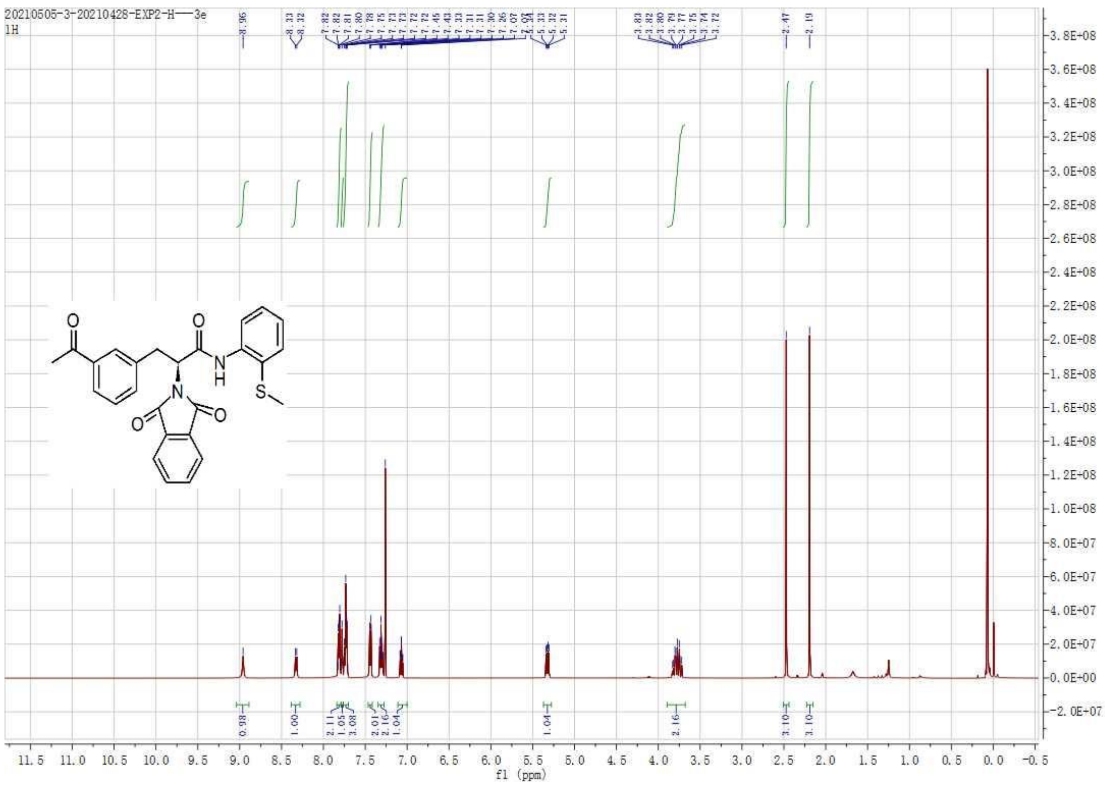
20210505-3-20210428-EXP2-H—3e
1H
f1 (ppm)

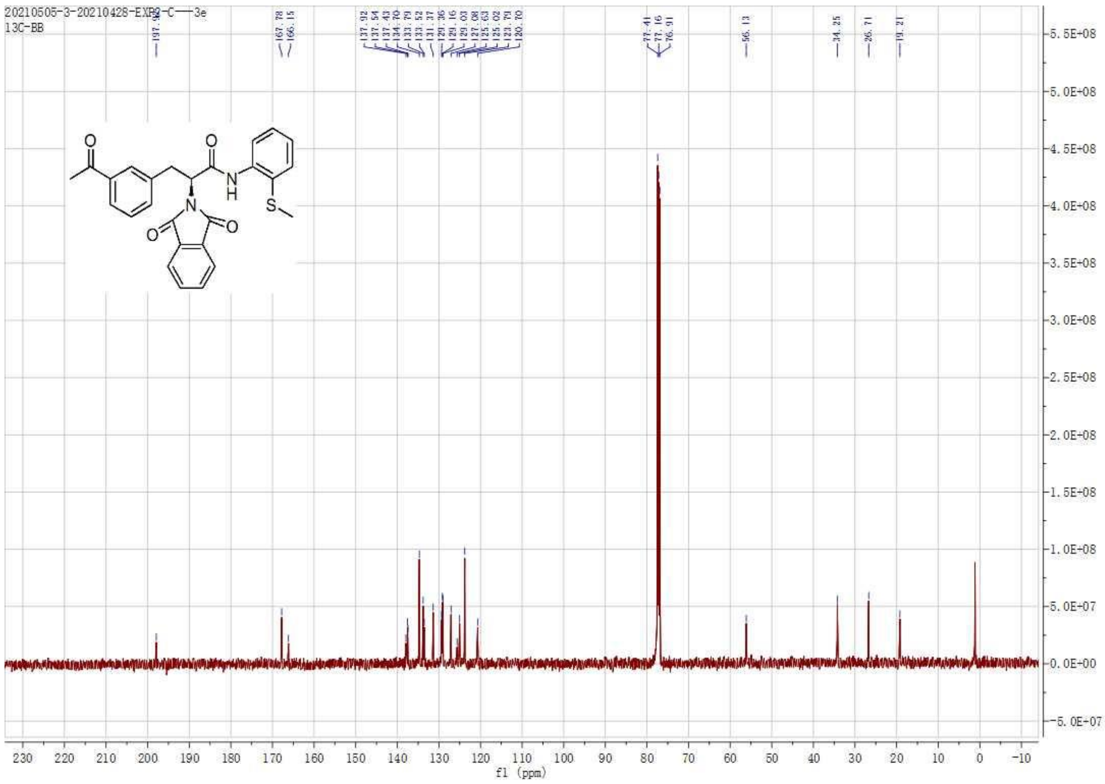
13C-BB
f1 (ppm)

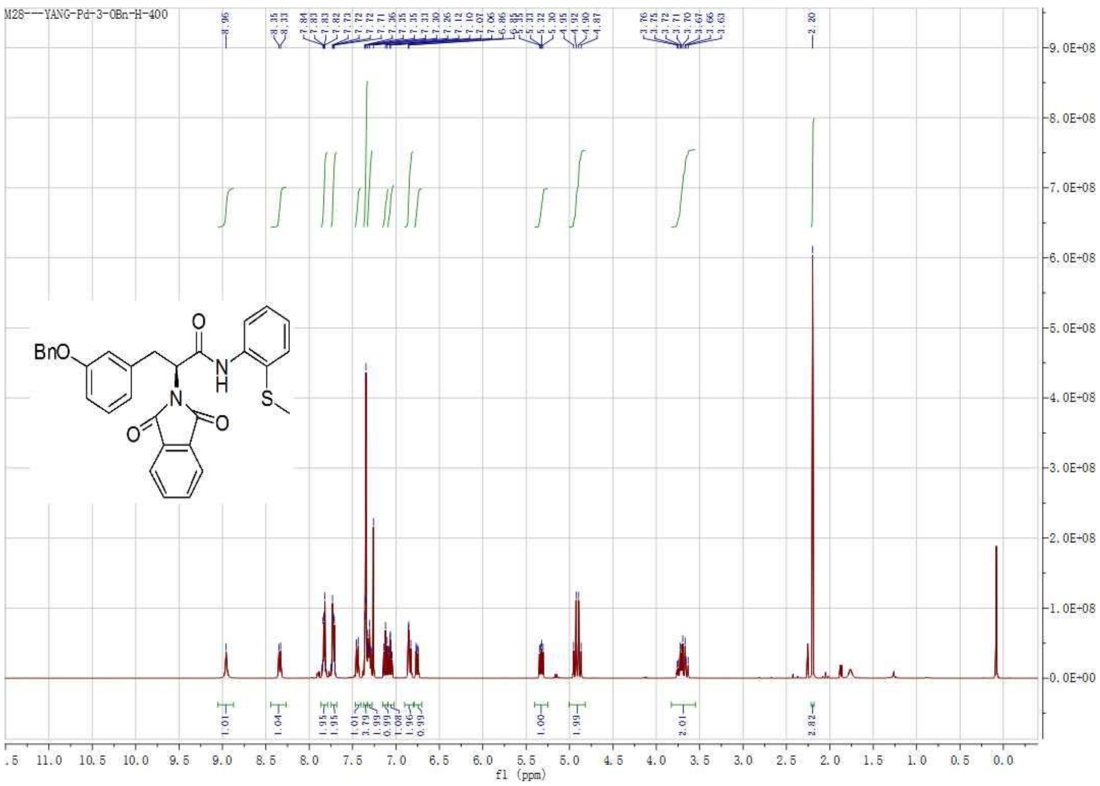

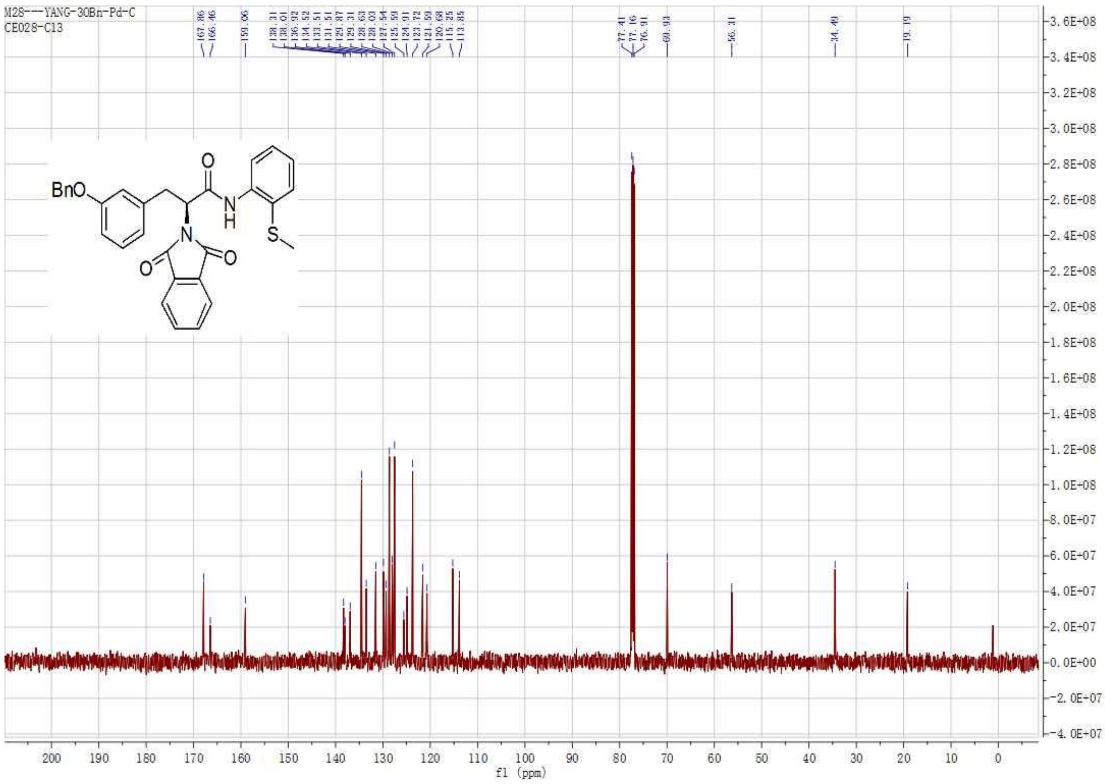

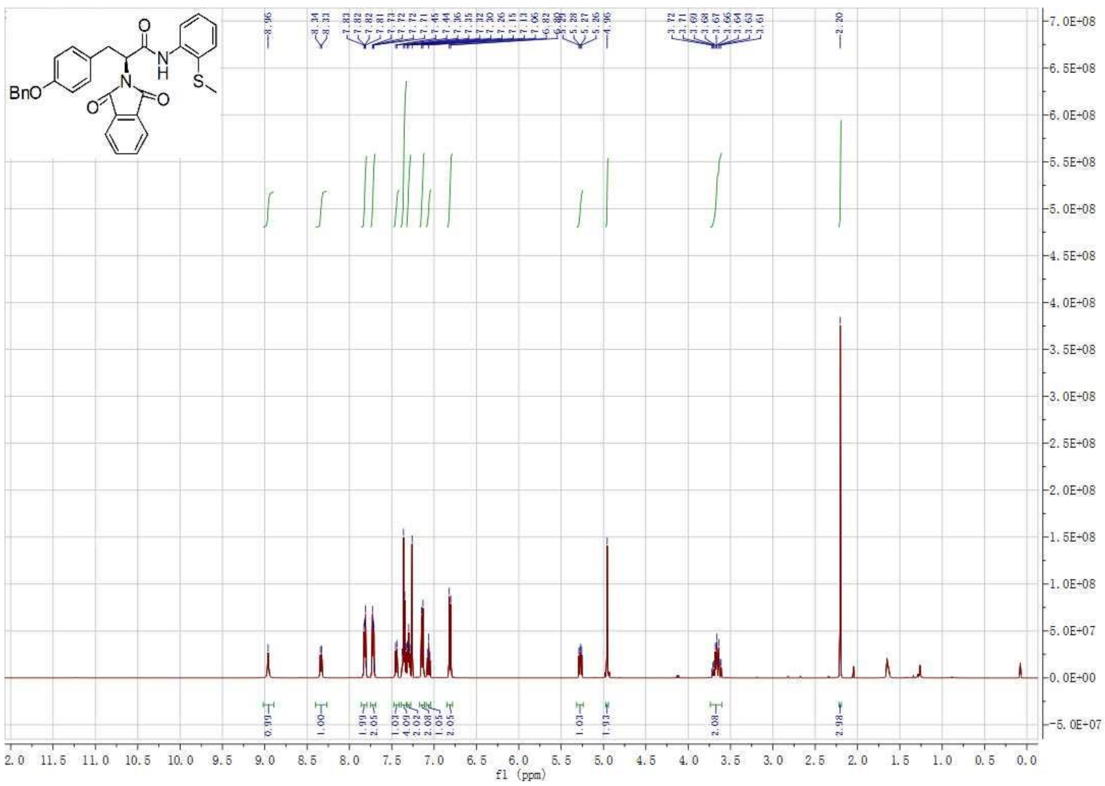

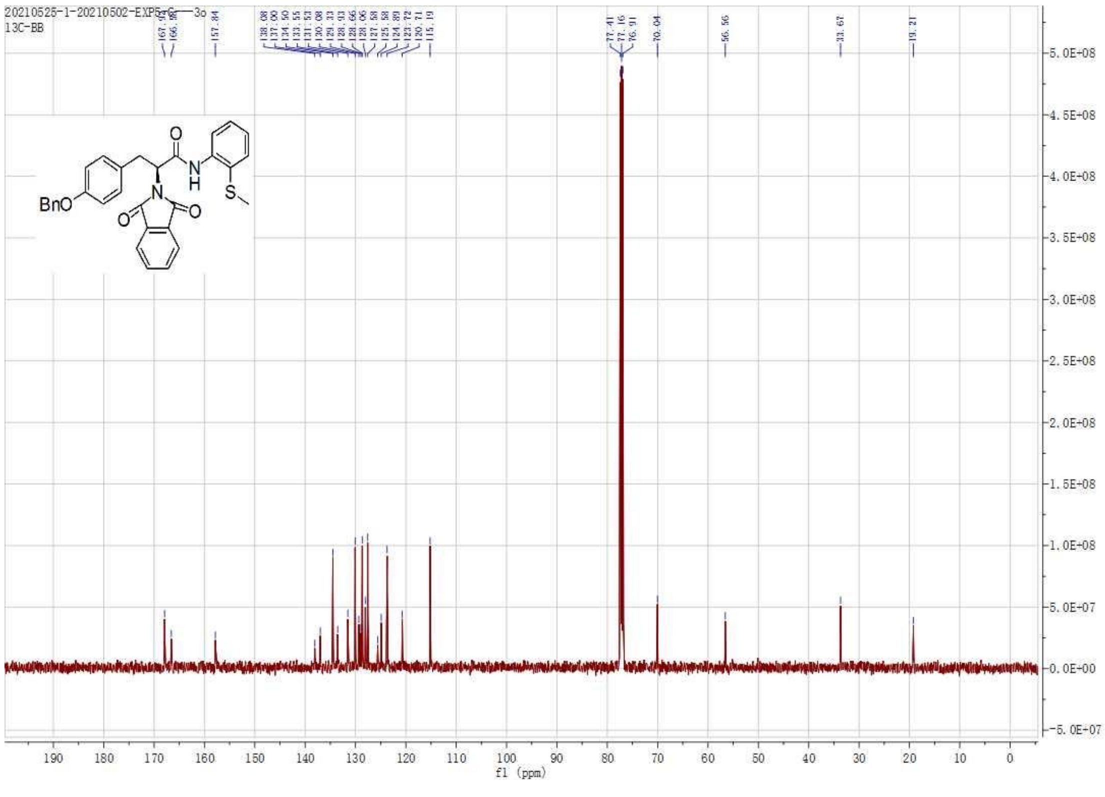
20210525-1-20210502-EXP5-C—3o
13C-BB

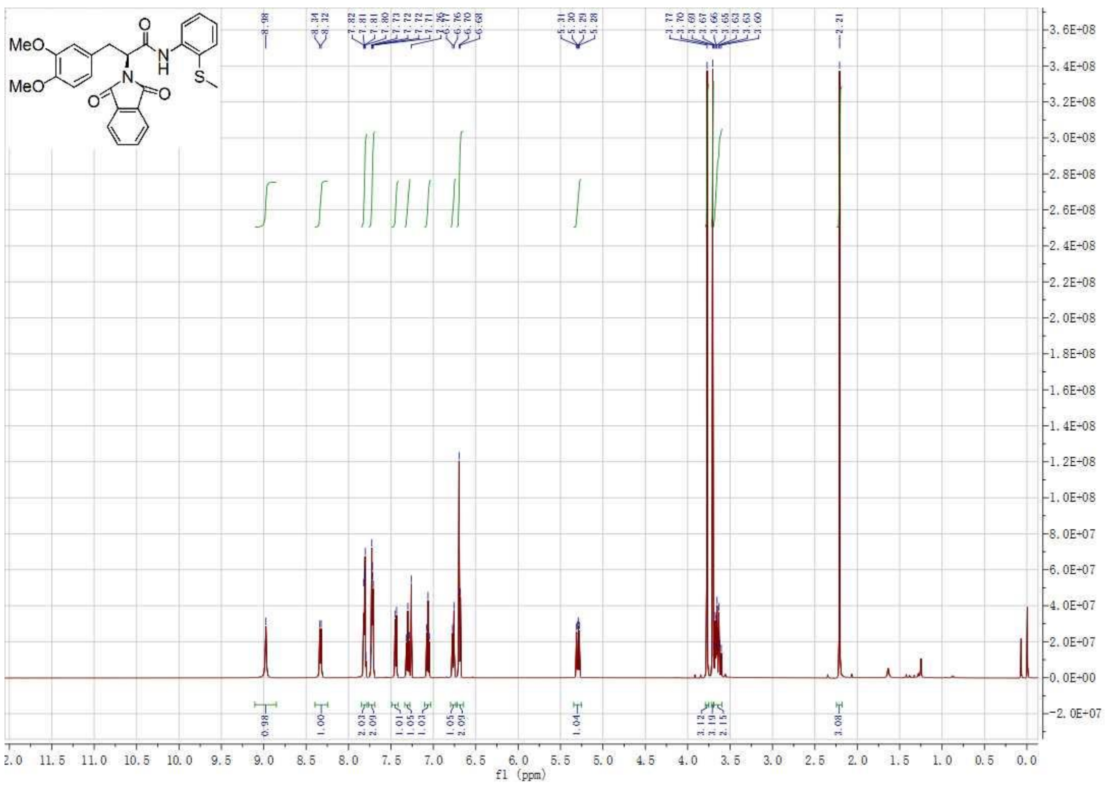

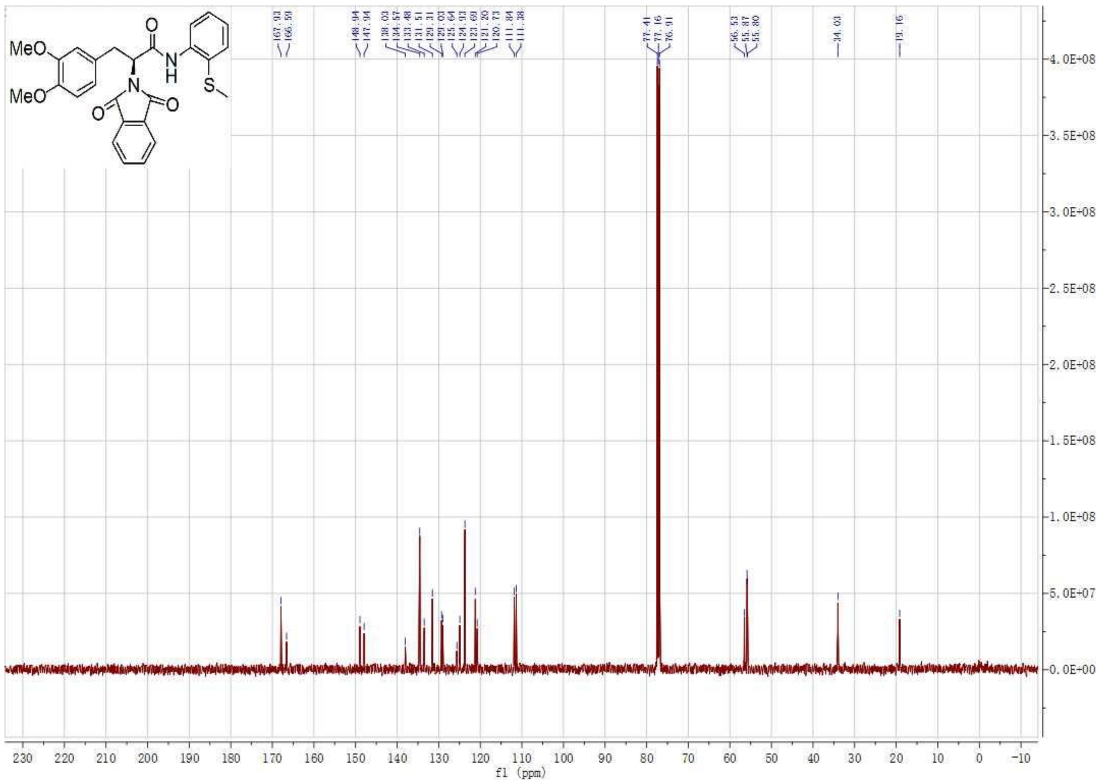

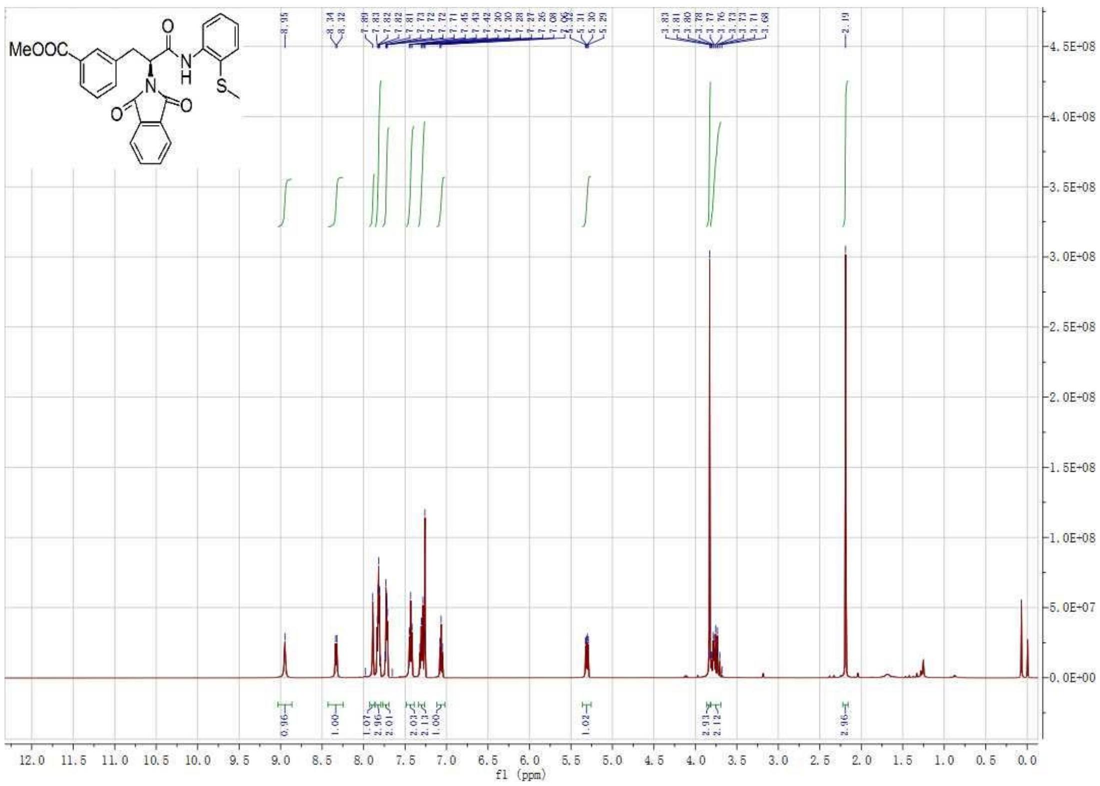

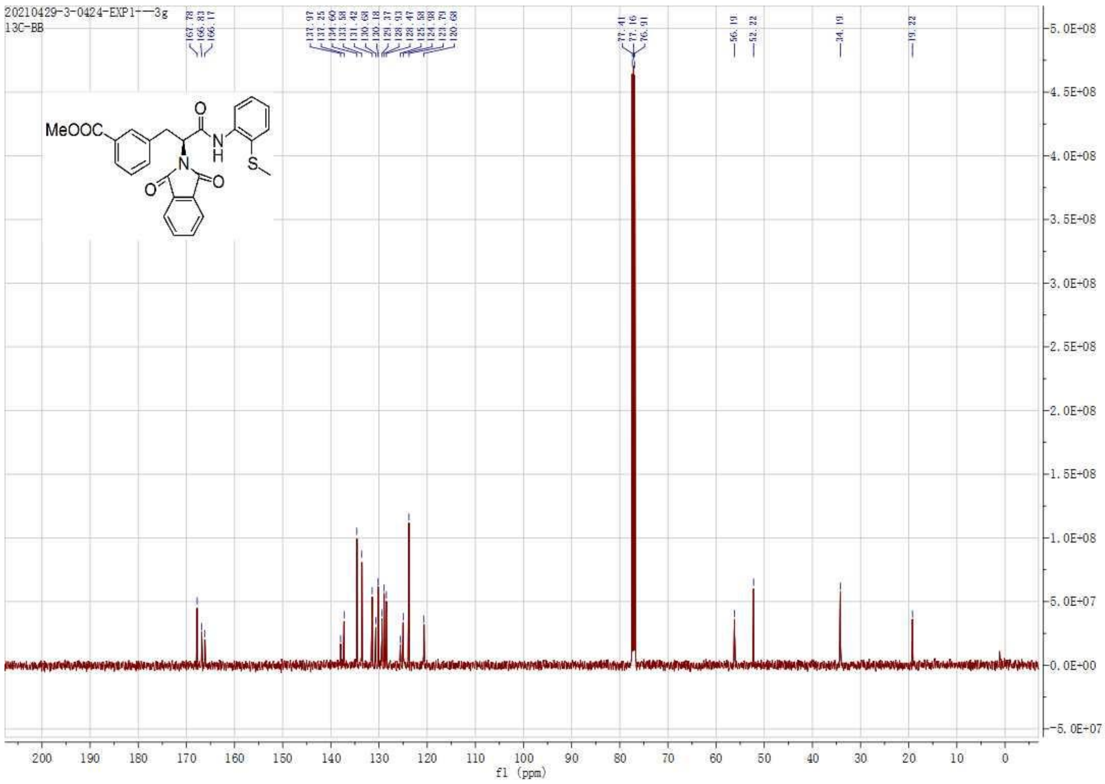
20210429-3-0424-EXP1---3g
13C-BB

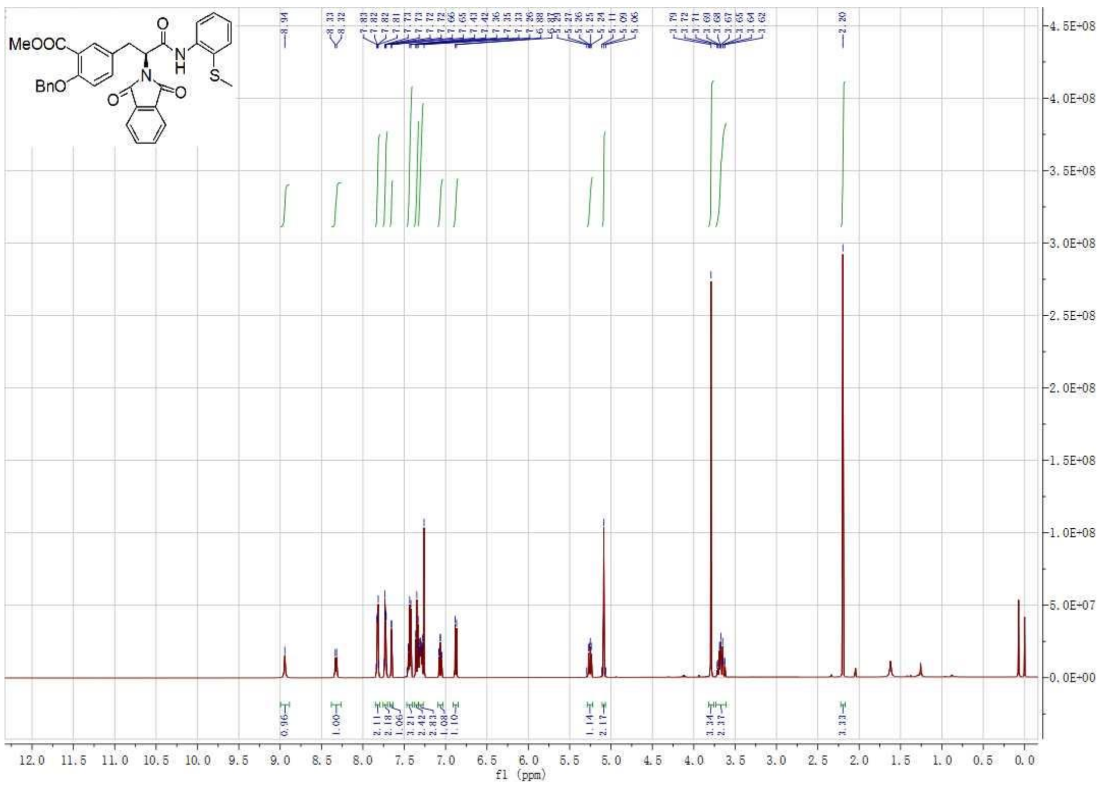
MeOOC
BnO
O
N
H
N
O
O
S
f1 (ppm)

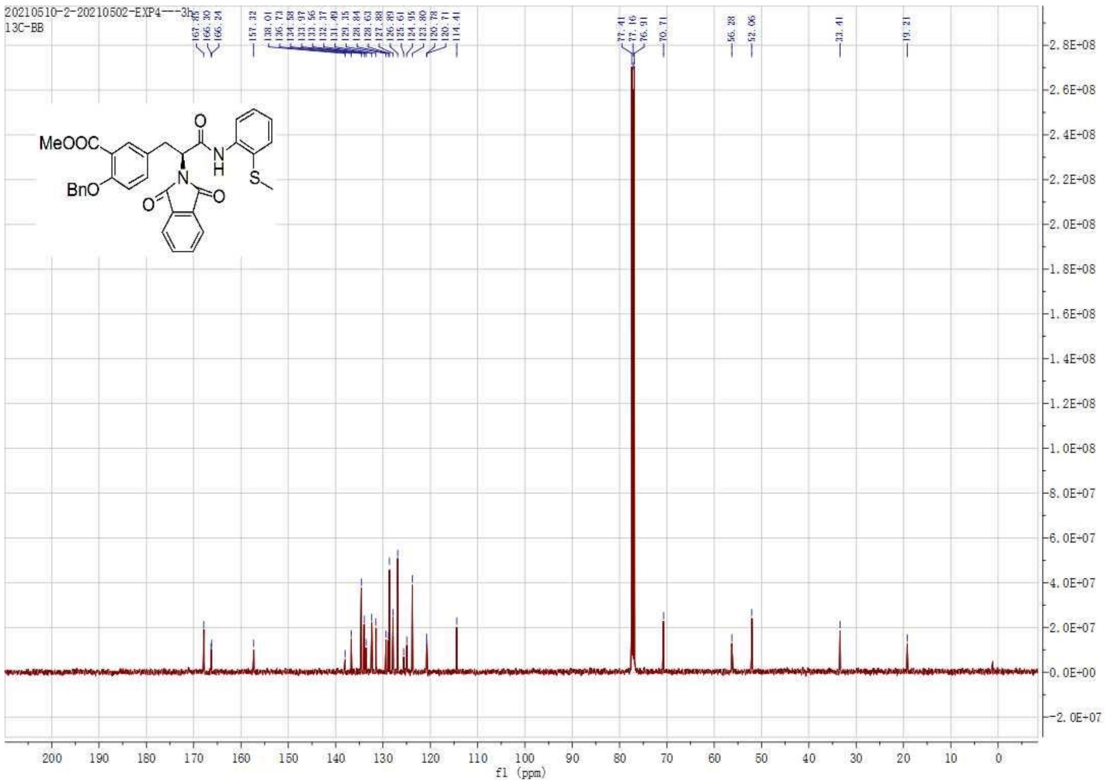
20210510-2-20210502-EXP4---3h
13C-BB
MeOOC
BnO
O
N
H
N
O
O
S
f1 (ppm)

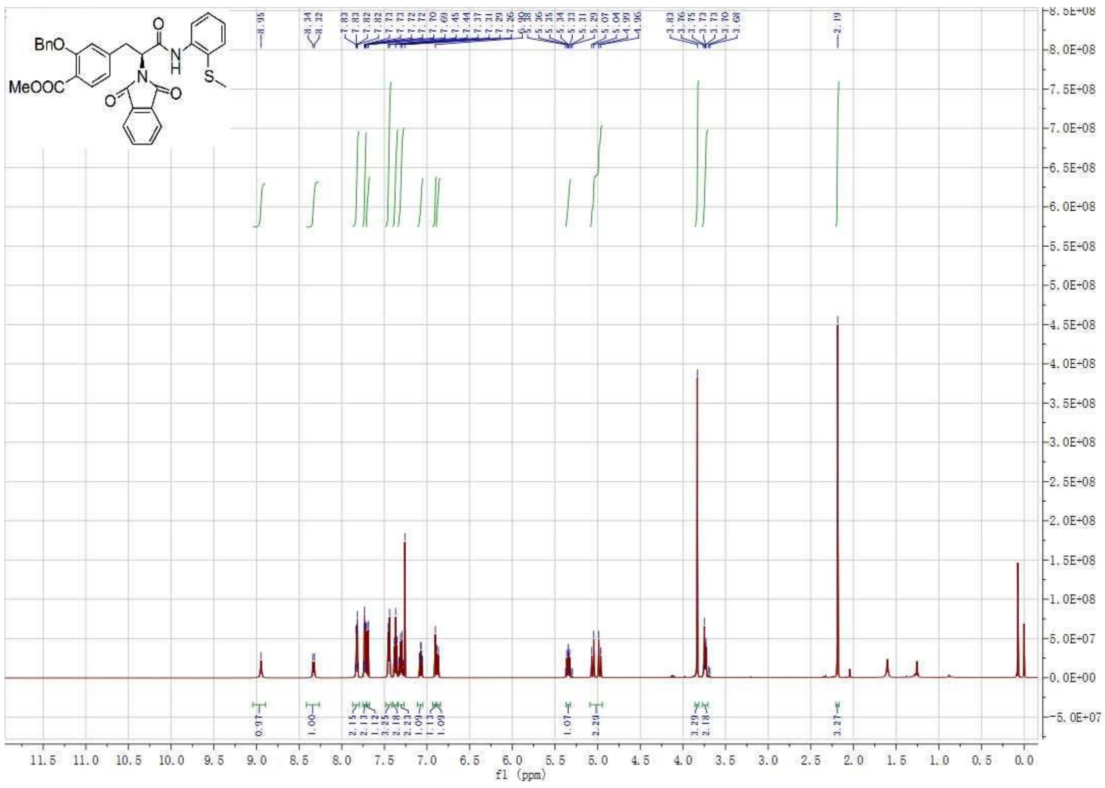
BnO
MeOOC
N
H
S
O
f1 (ppm)

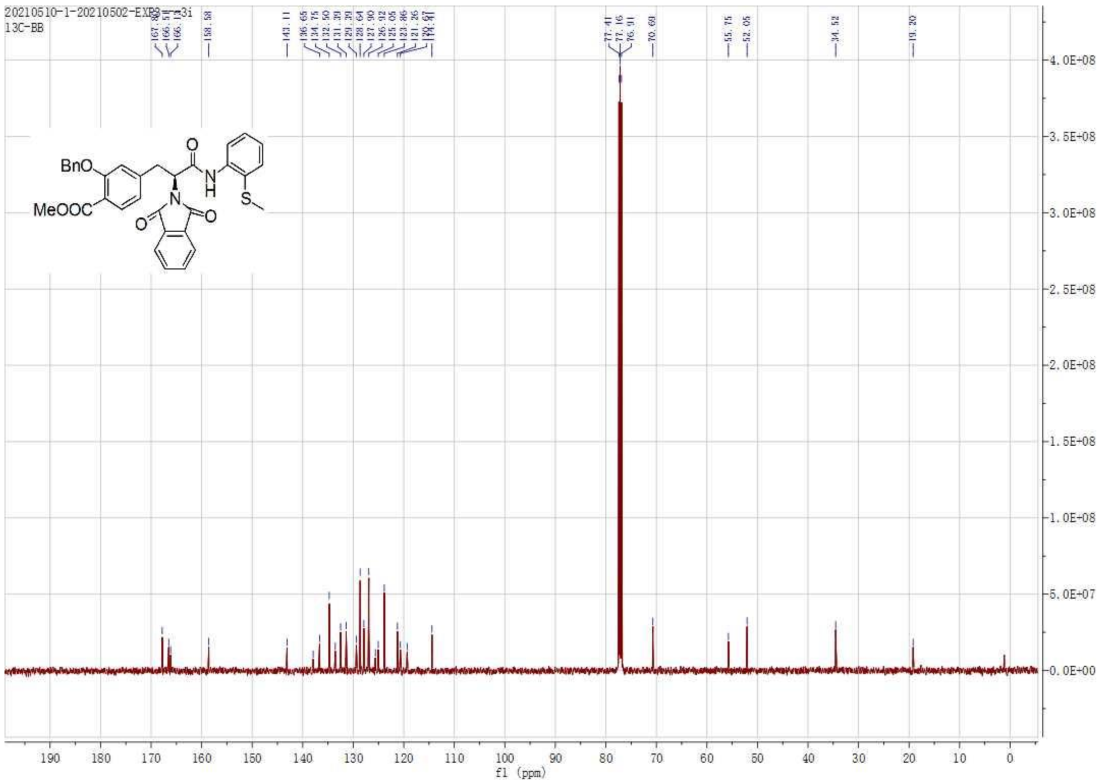
20210510-1-20210502-EXP8--3i
13C-BB
BnO
MeOOC
N
H
S
O
f1 (ppm)

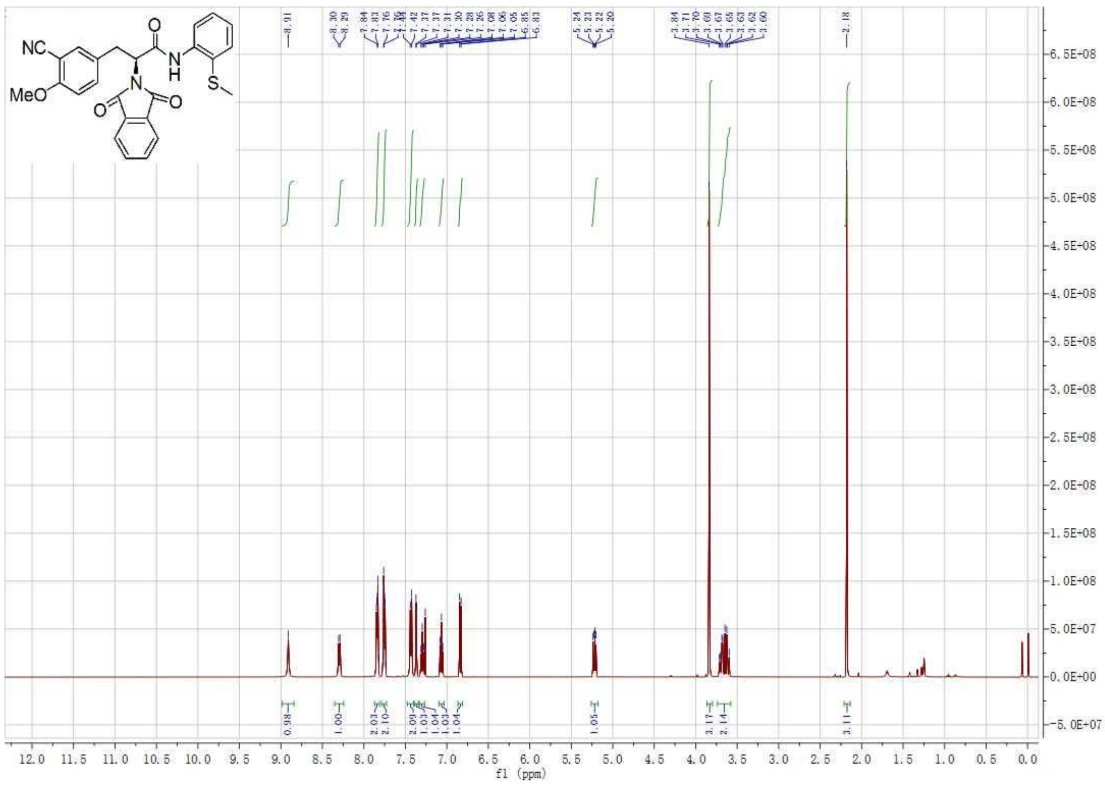
NC
MeO
f1 (ppm)

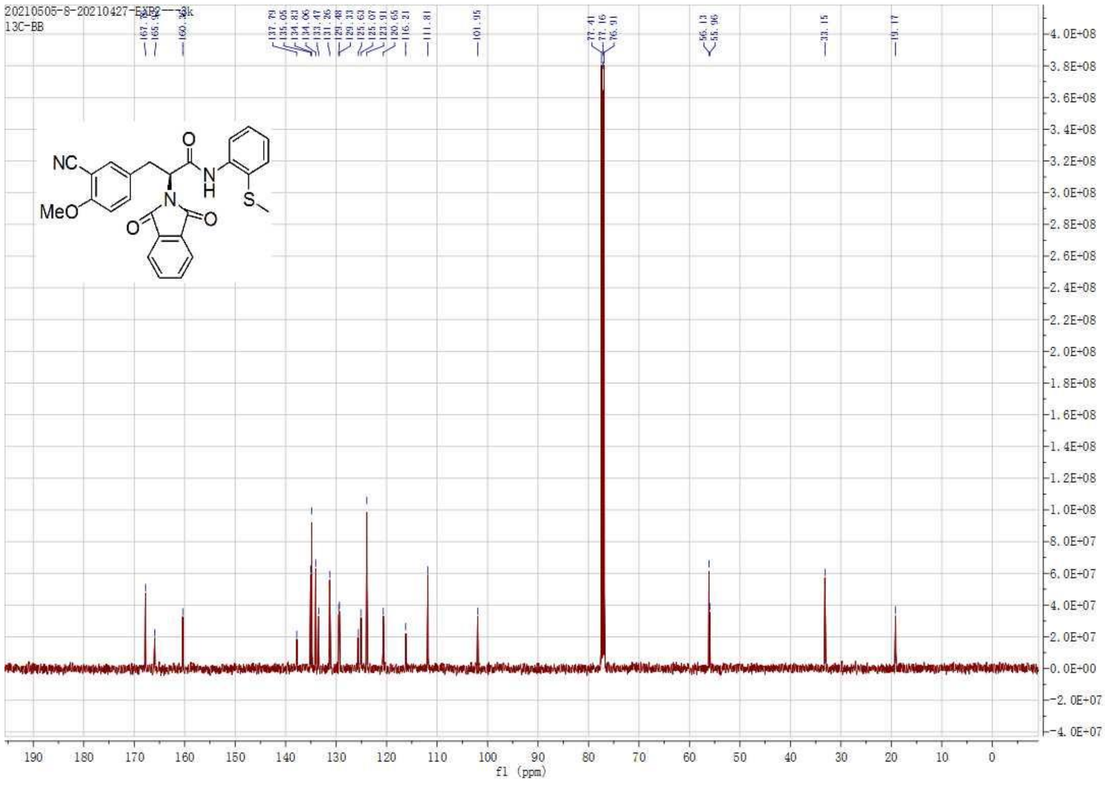
13C-BB
NC
MeO
f1 (ppm)

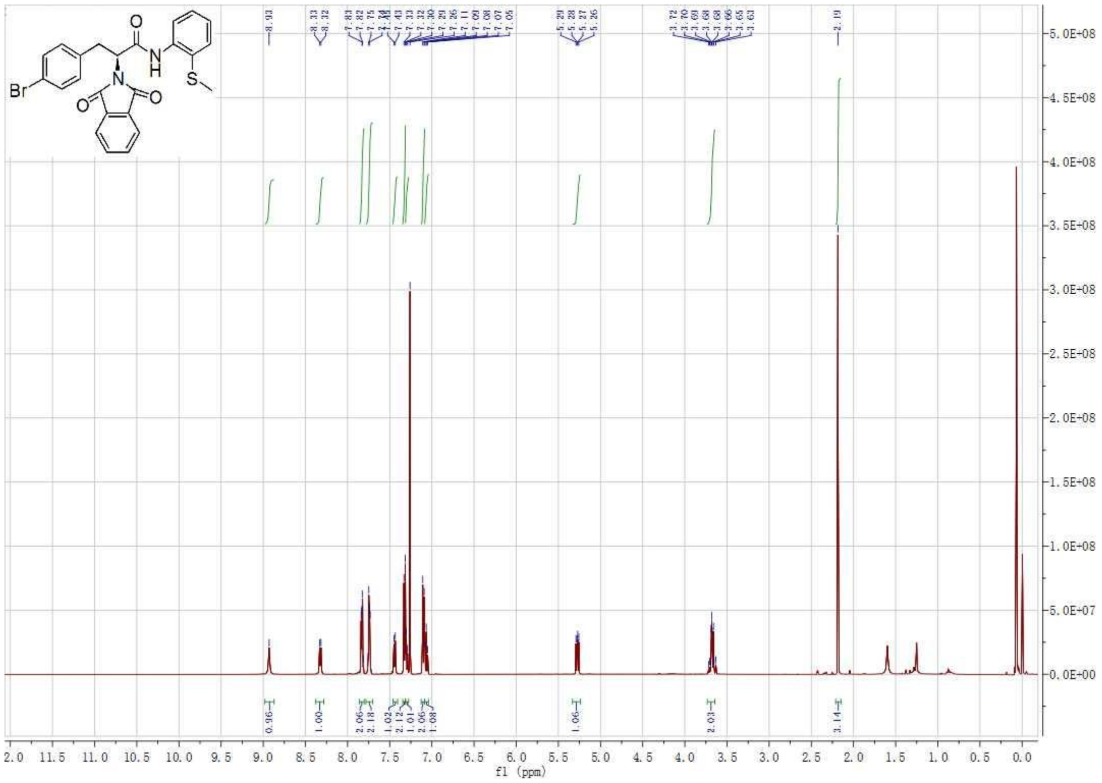
Br
O
N
H
S
f1 (ppm)

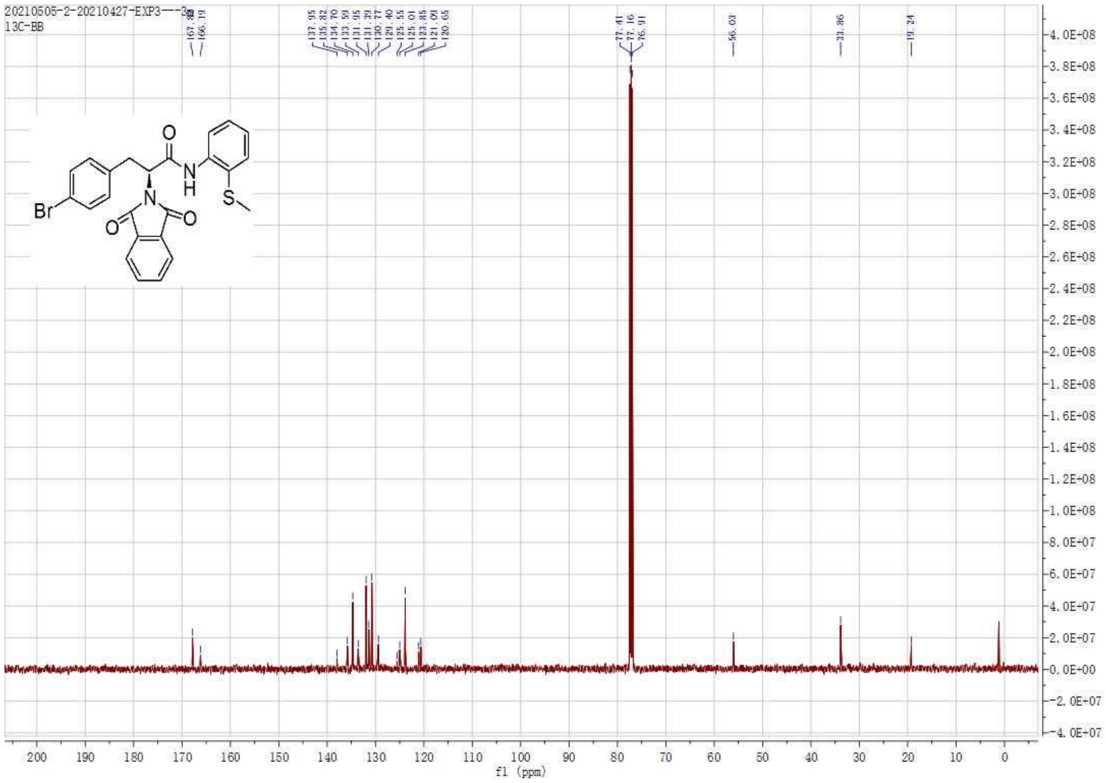
20210505-2-20210427-EXP3---3
13C-BB
Br
O
N
H
S
f1 (ppm)

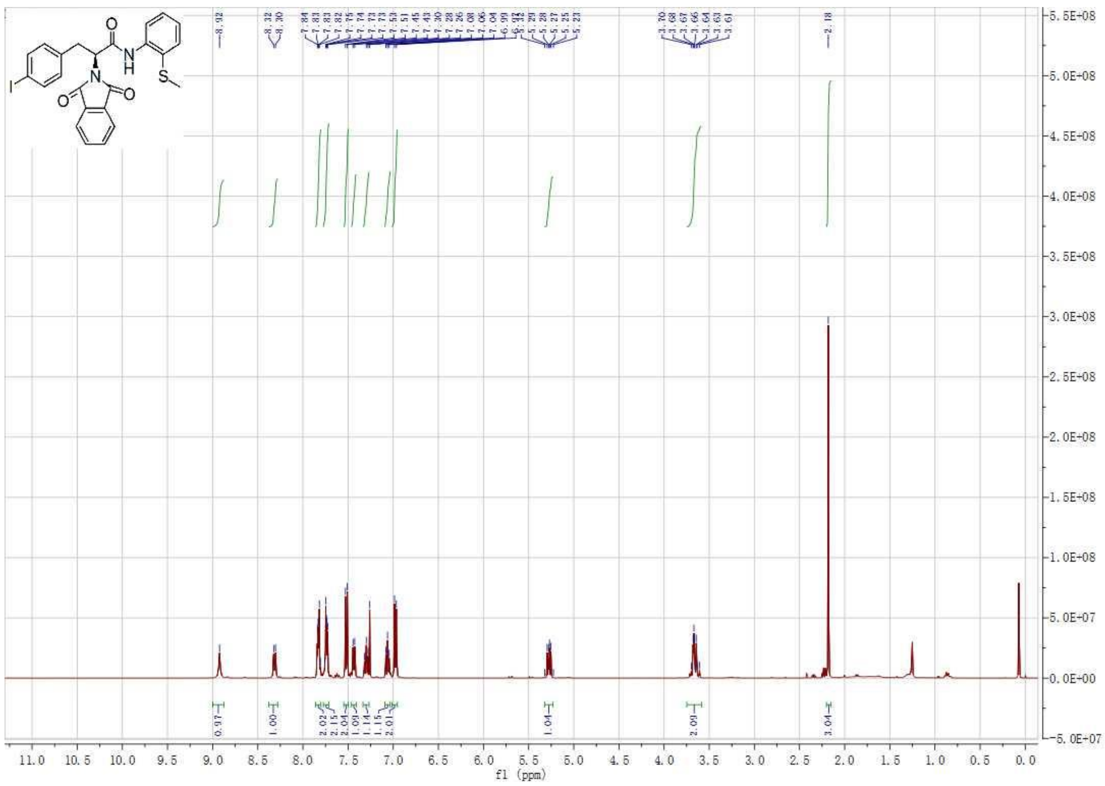
8.92
8.32
8.30
7.84
7.83
7.83
7.82
7.75
7.74
7.73
7.73
7.53
7.51
7.45
7.43
7.30
7.28
7.26
7.08
7.06
7.04
6.99
5.32
5.29
5.28
5.27
5.25
5.23
3.70
3.68
3.67
3.66
3.64
3.63
3.61
2.18
0.97
1.00
2.02
2.15
2.04
1.09
1.14
1.15
2.01
1.04
2.09
3.04
f1 (ppm)

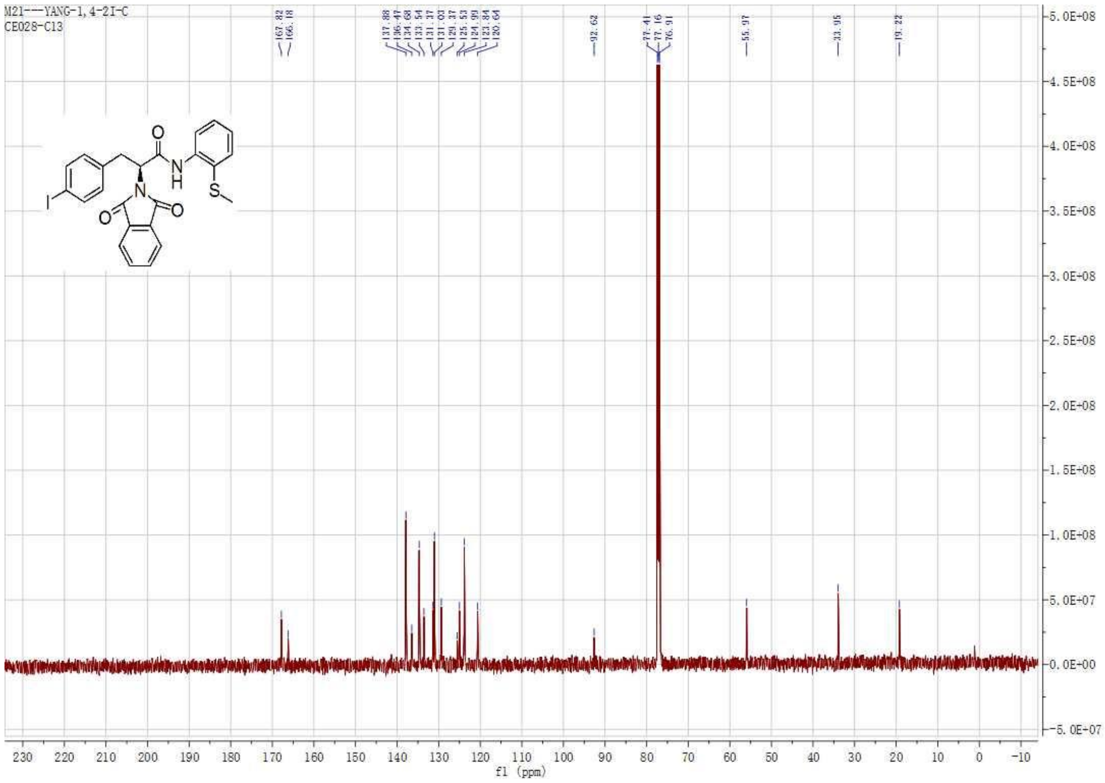
M21---YANG-1,4-2I-C
CE028-C13
167.82
166.18
137.88
136.47
134.68
133.54
131.37
131.03
129.37
125.53
124.99
123.84
120.64
92.62
77.41
77.16
76.91
55.97
33.95
19.22
f1 (ppm)

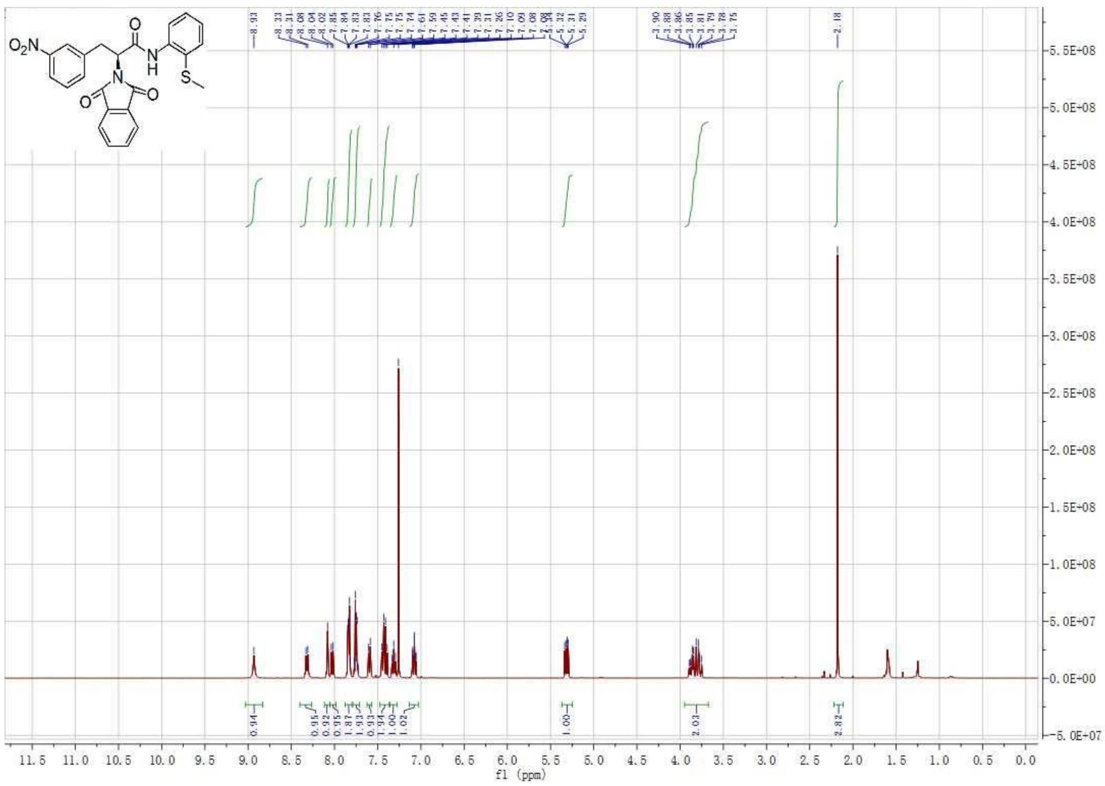

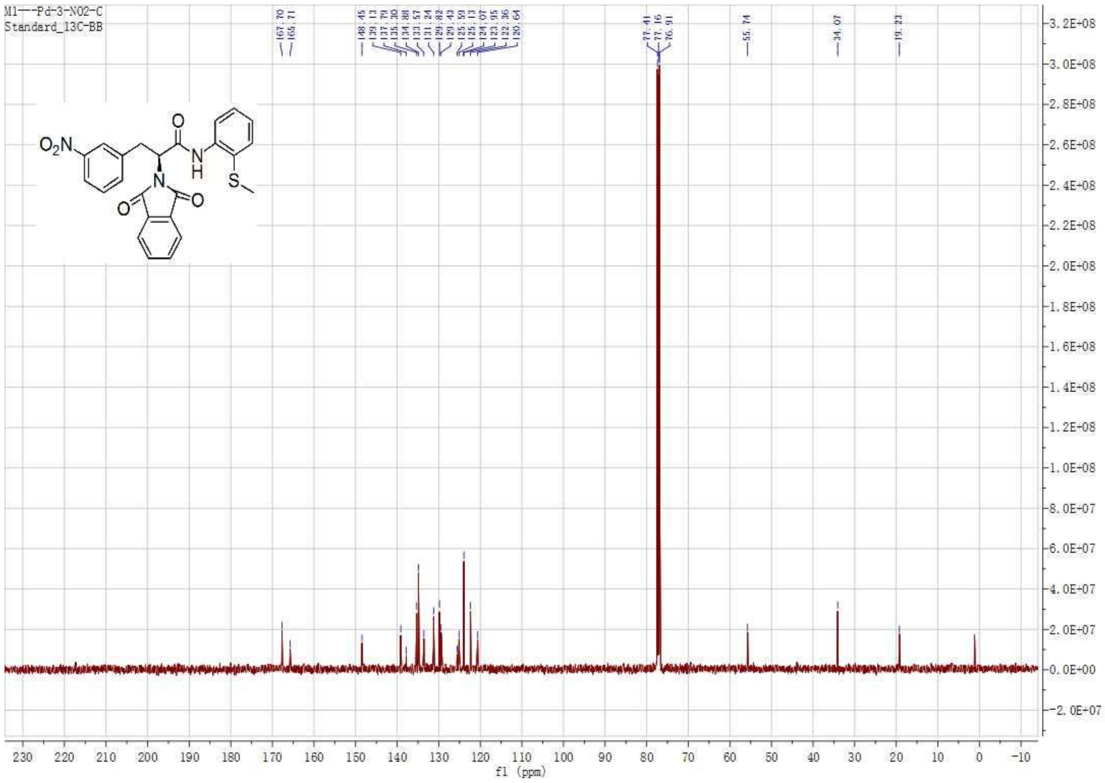

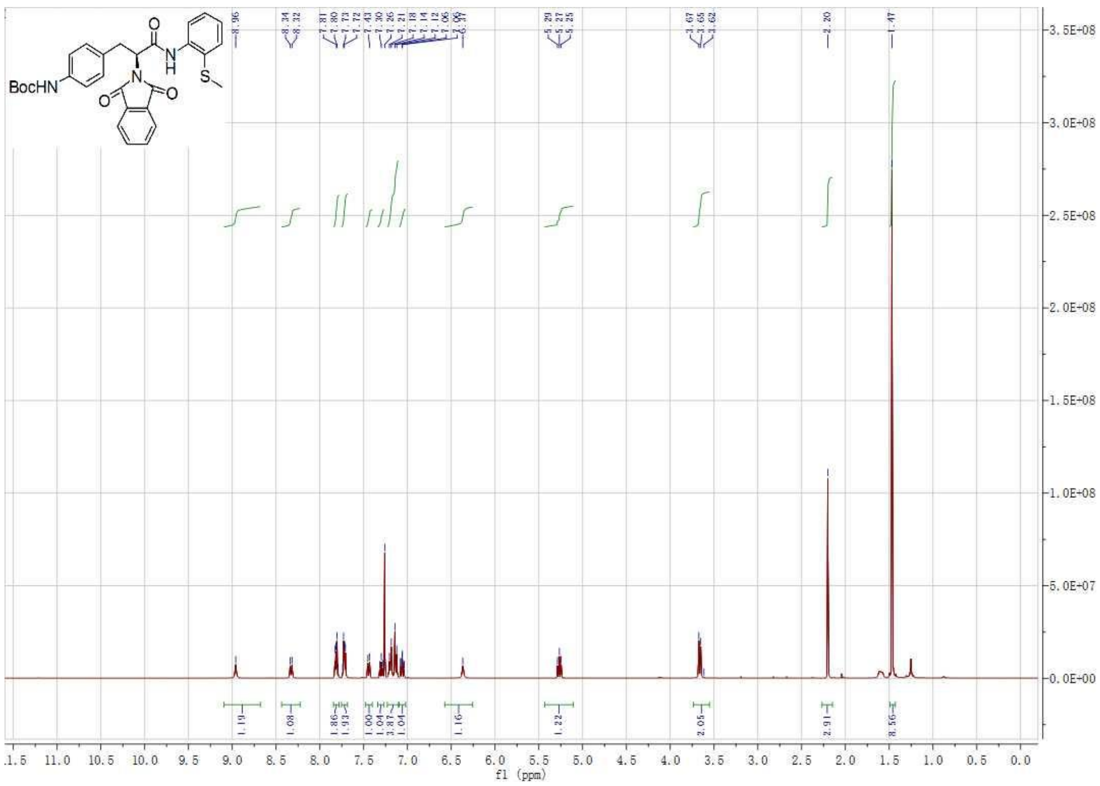

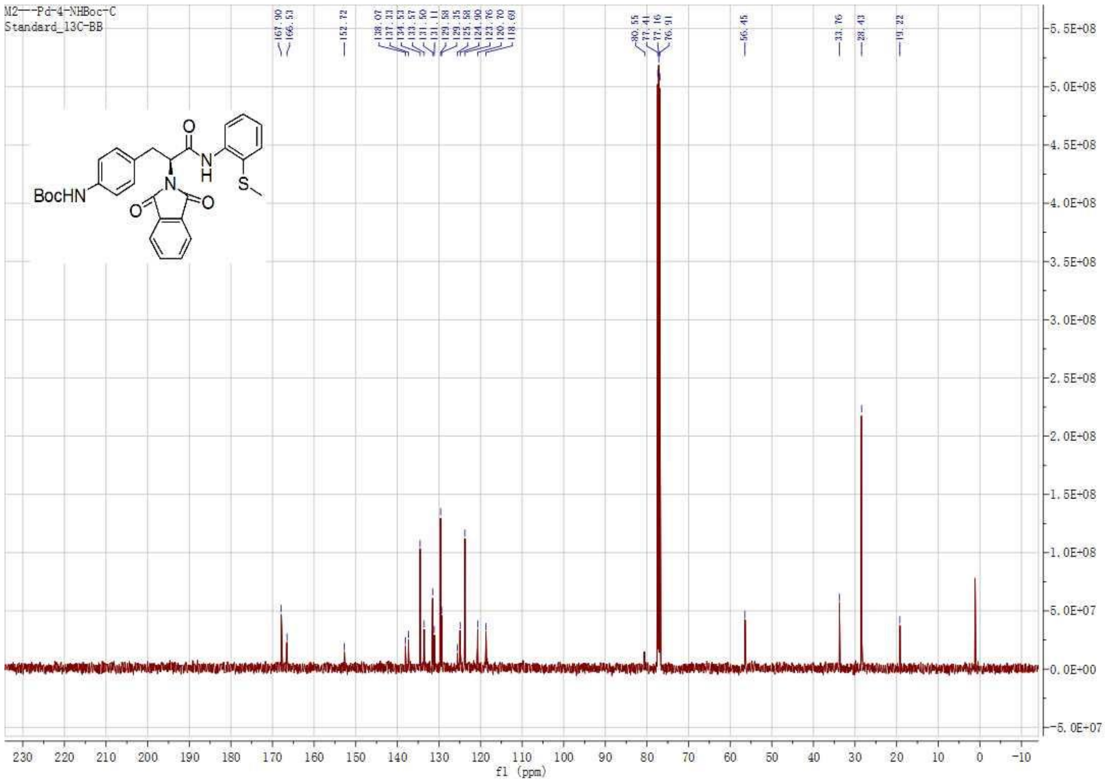

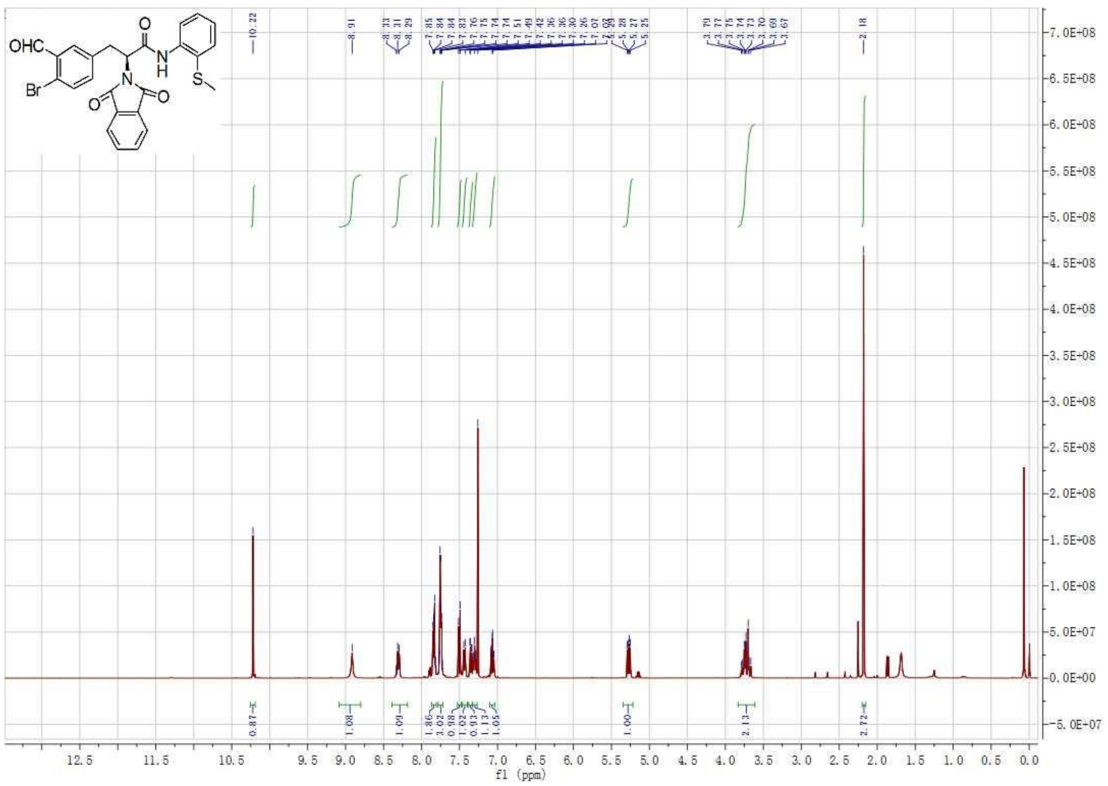
OHC
Br
O
N
H
S
f1 (ppm)

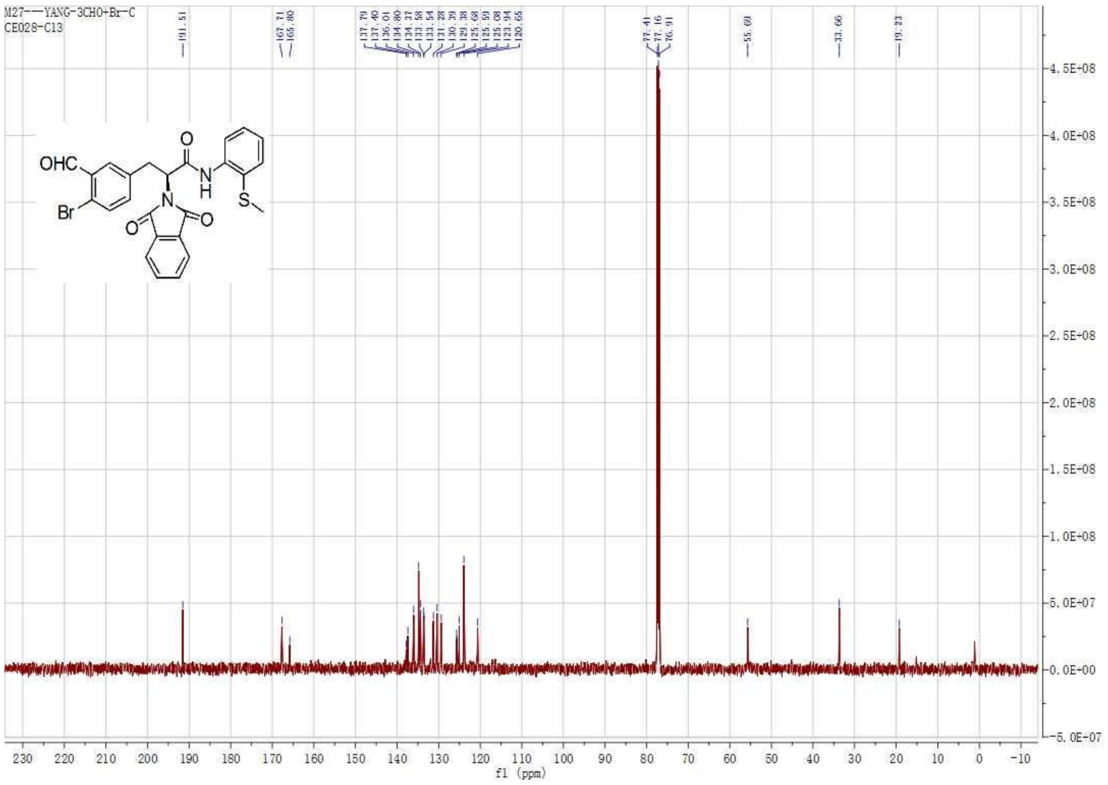
M27---YANG-3CHO+Br-C
CE028-C13
OHC
Br
O
N
H
S
f1 (ppm)

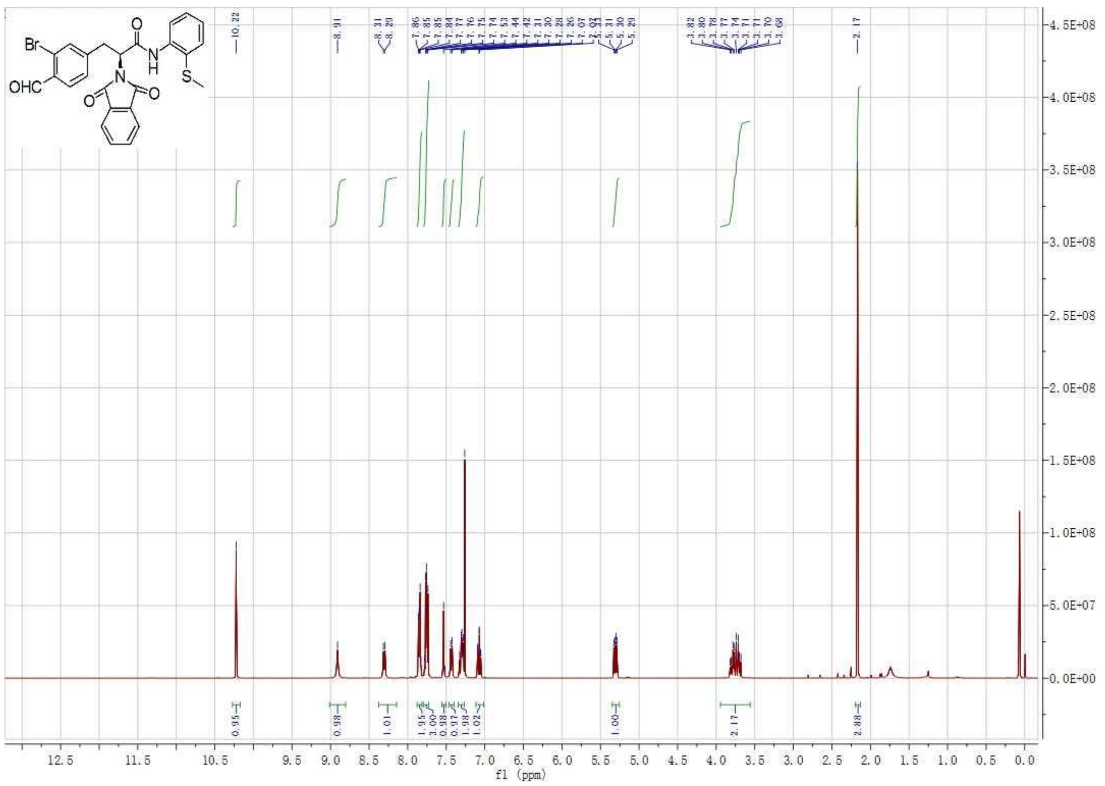

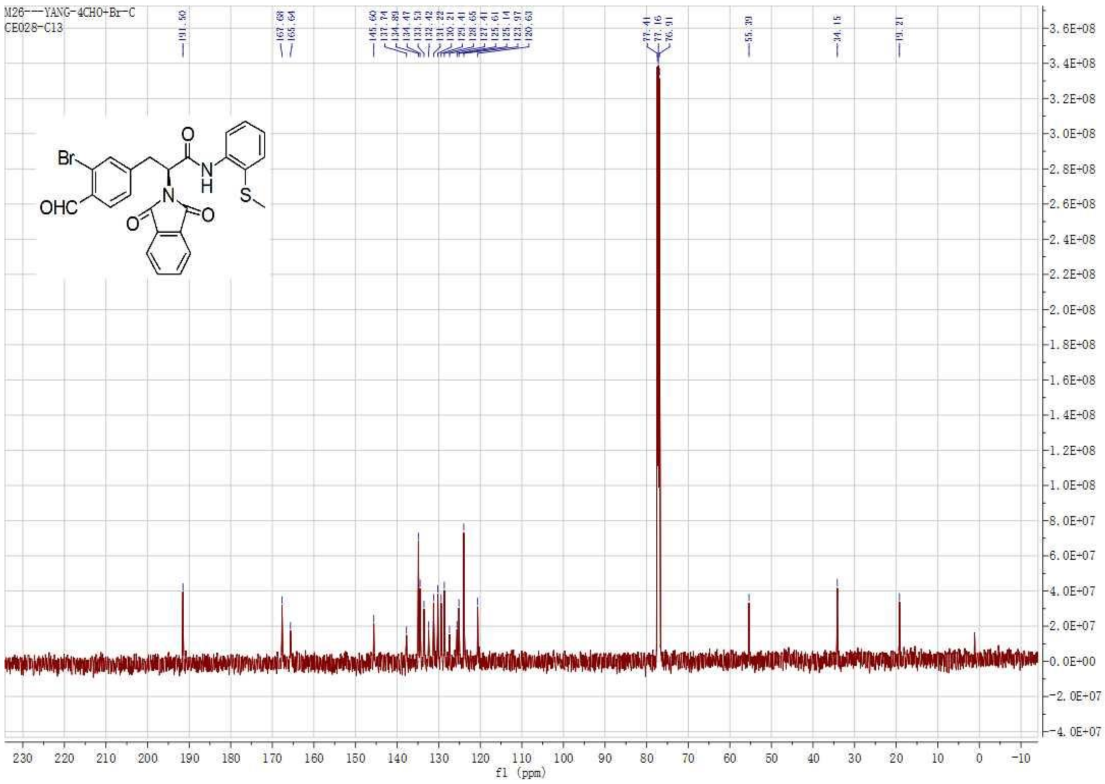

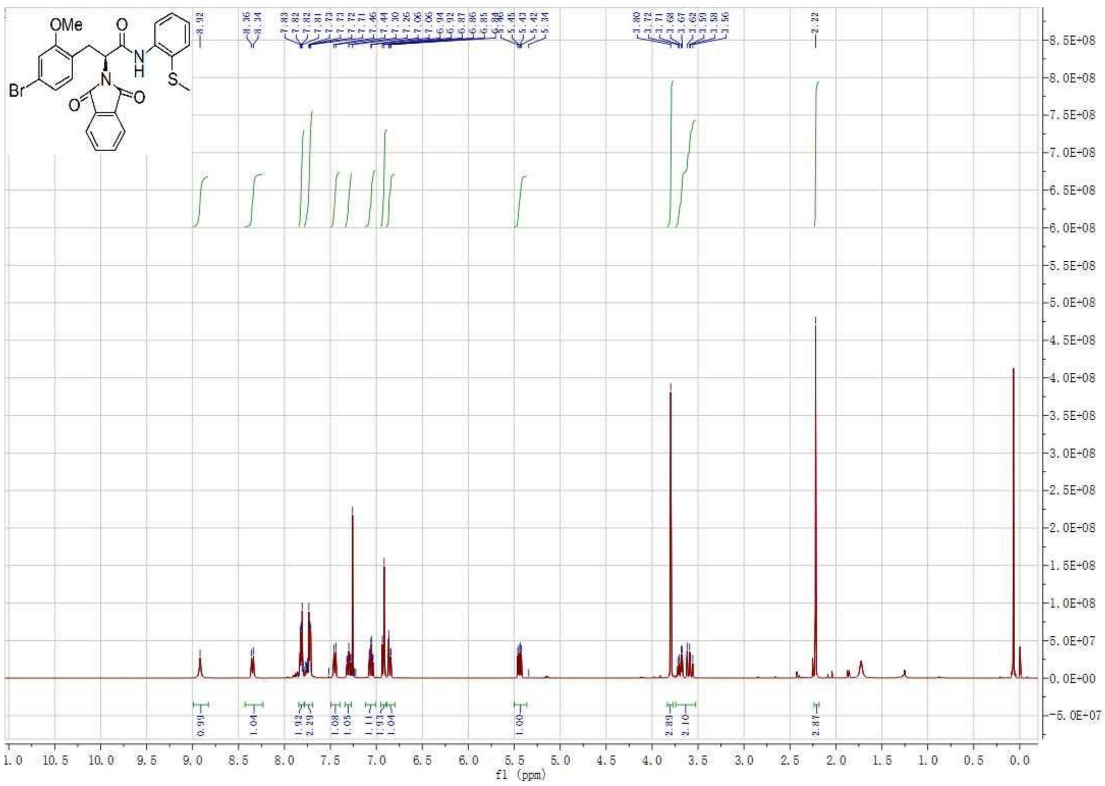

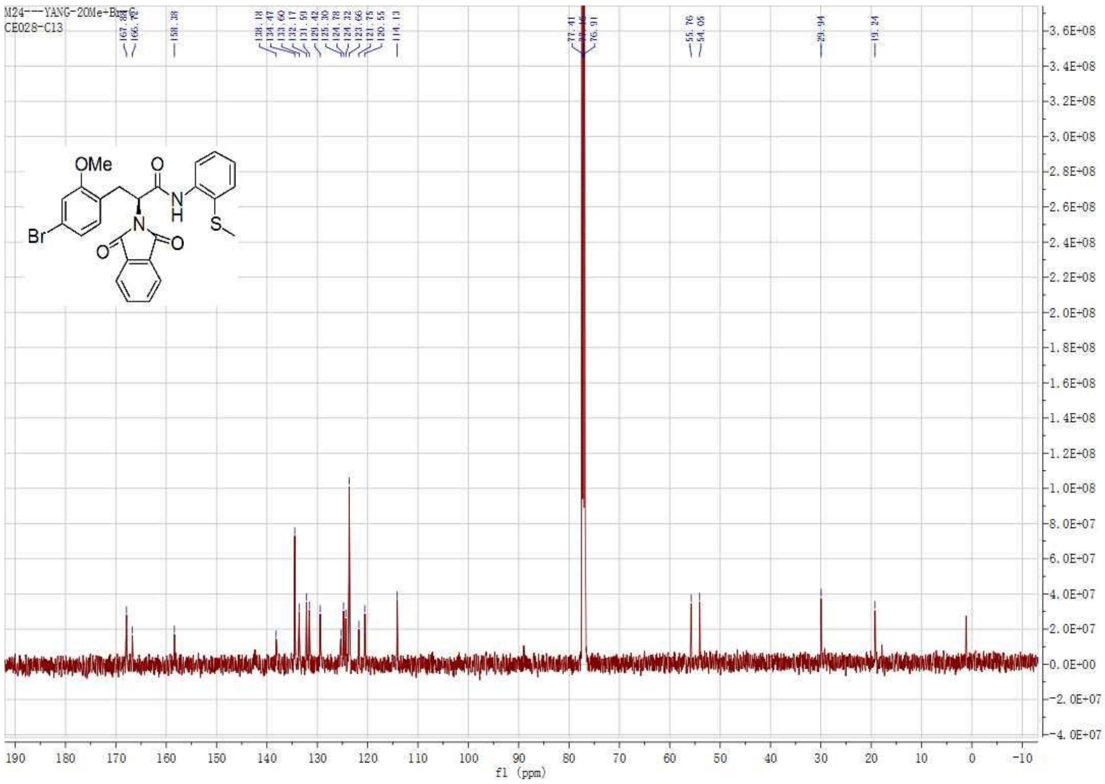

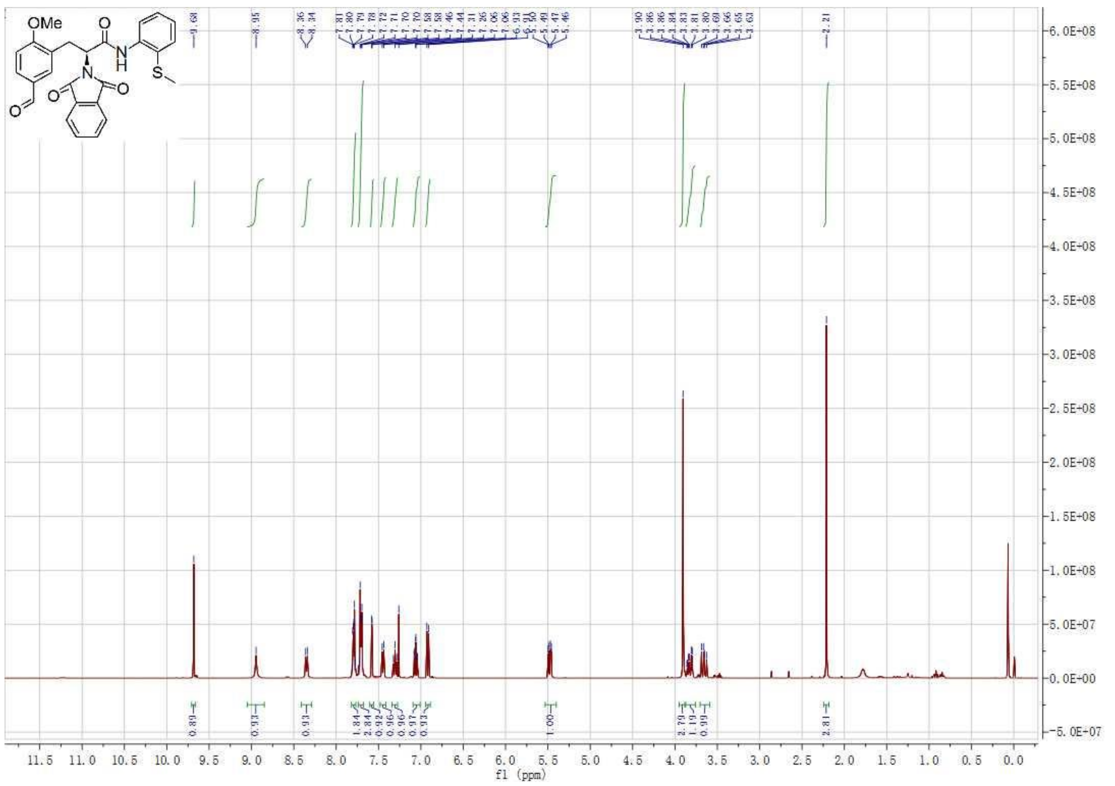

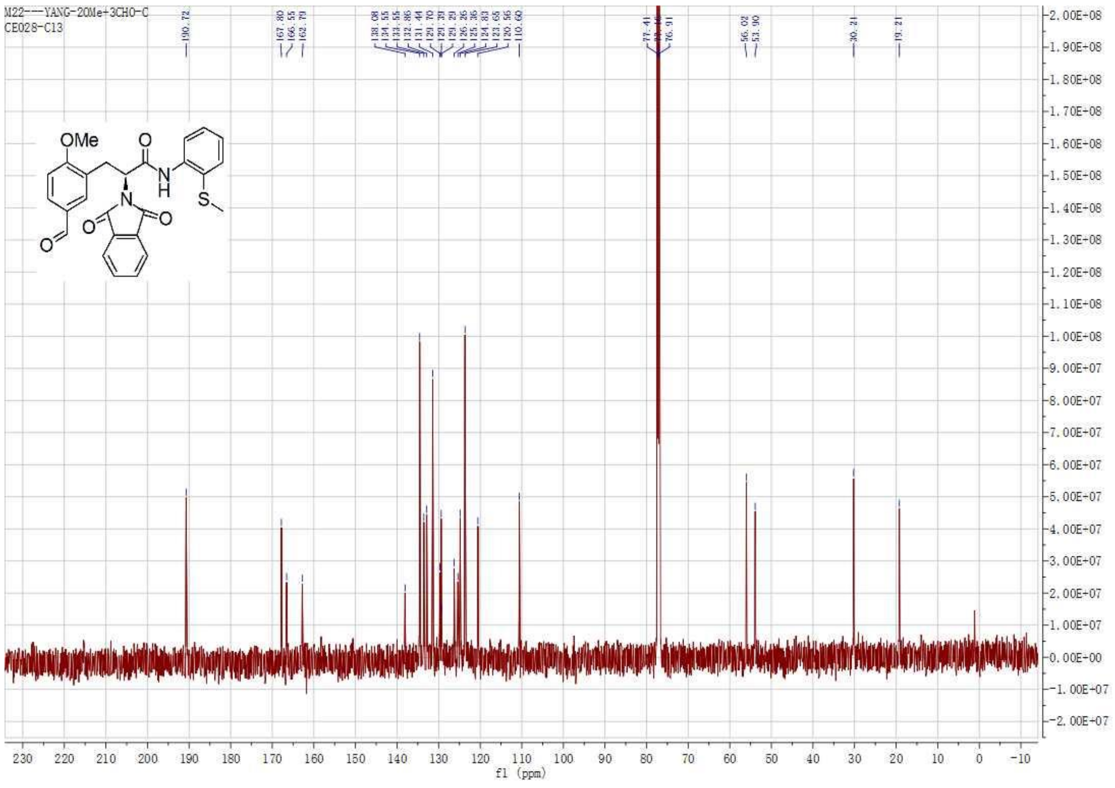

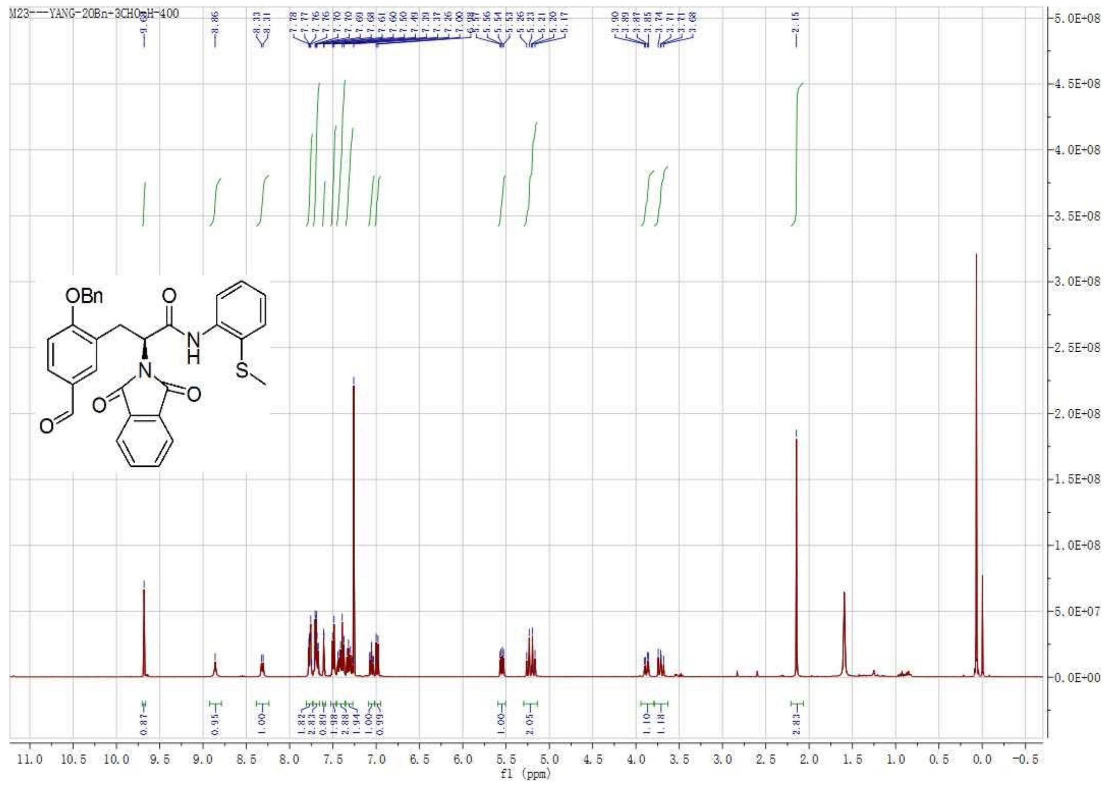

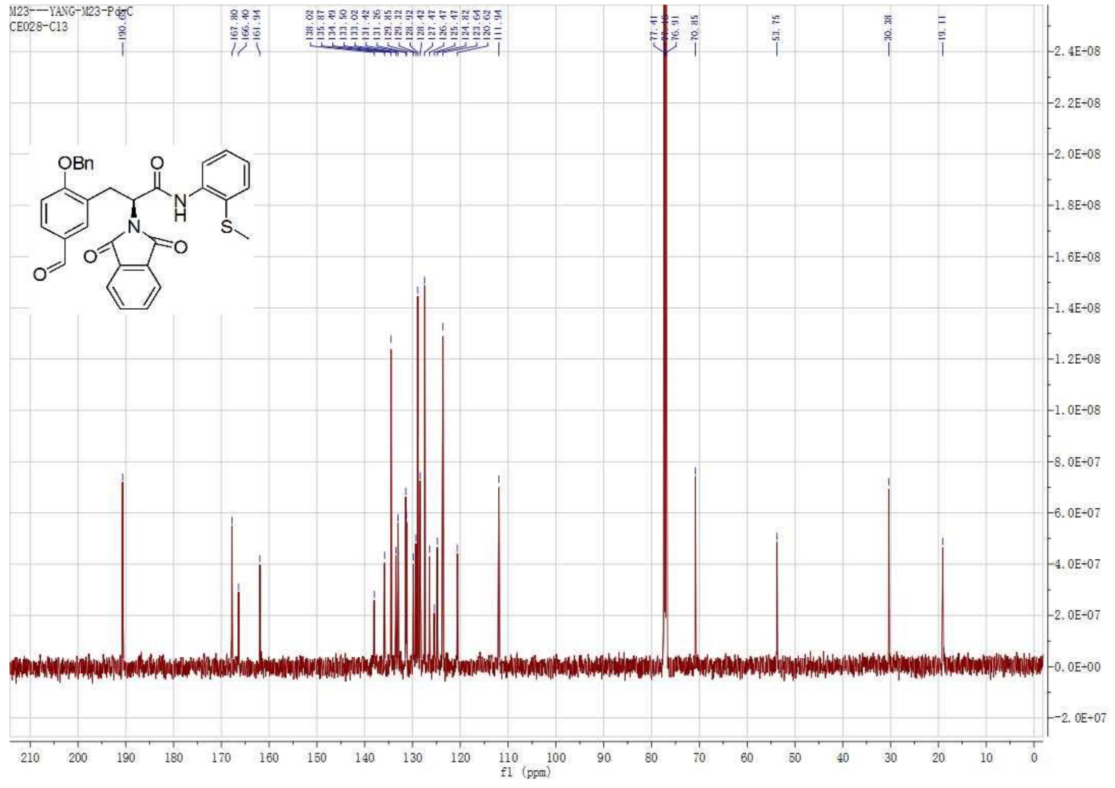

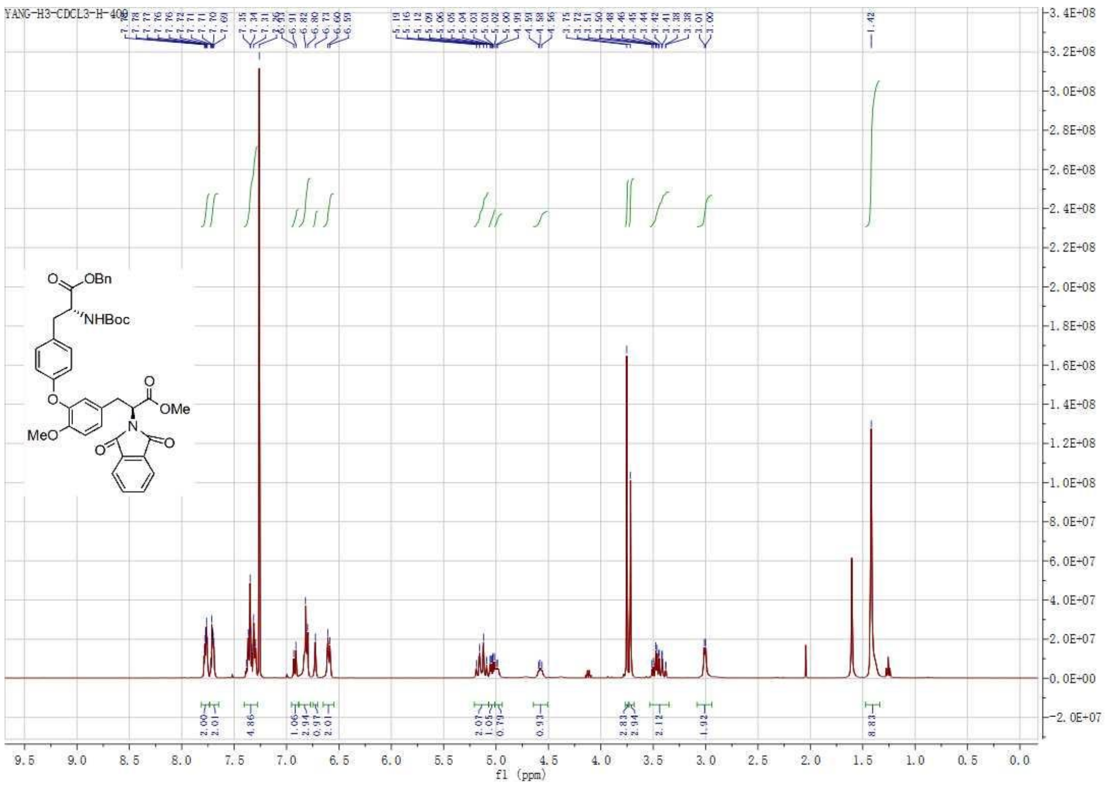
YANG-H3-CDCL3-H-400
OBn
NHBoc
OMe
MeO
f1 (ppm)

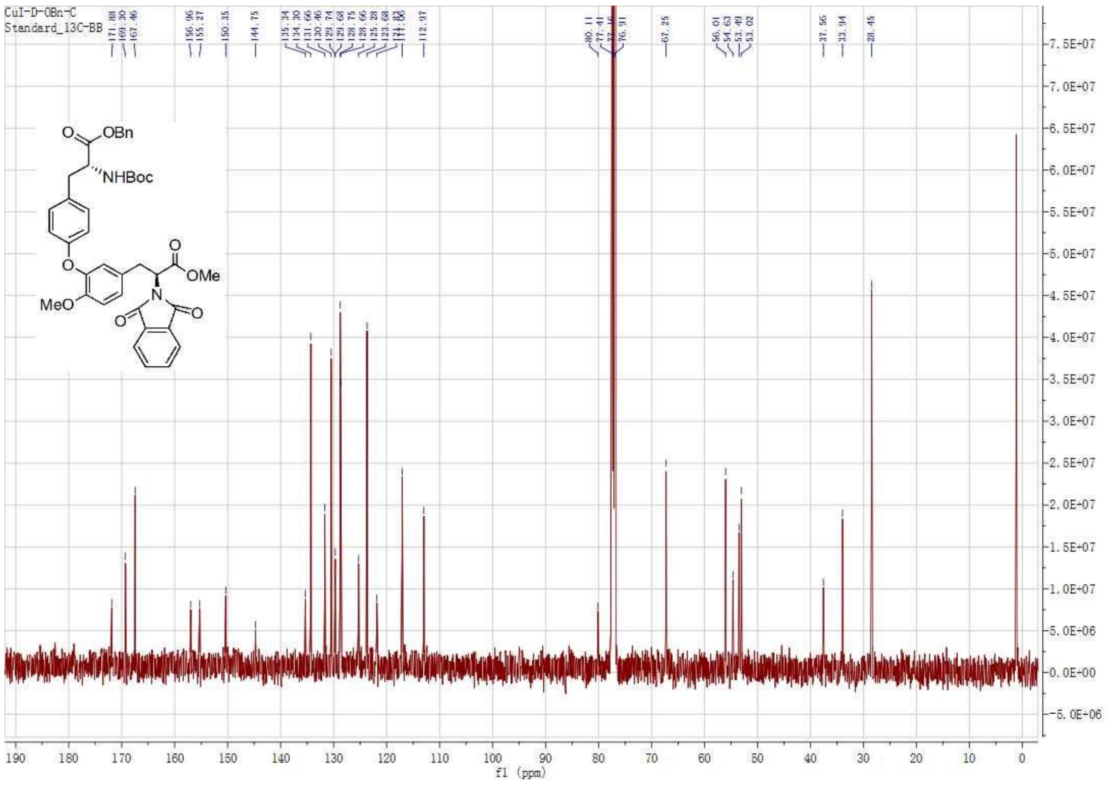
CuI-D-OBn-C
Standard_13C-BB
OBn
NHBoc
OMe
MeO
f1 (ppm)

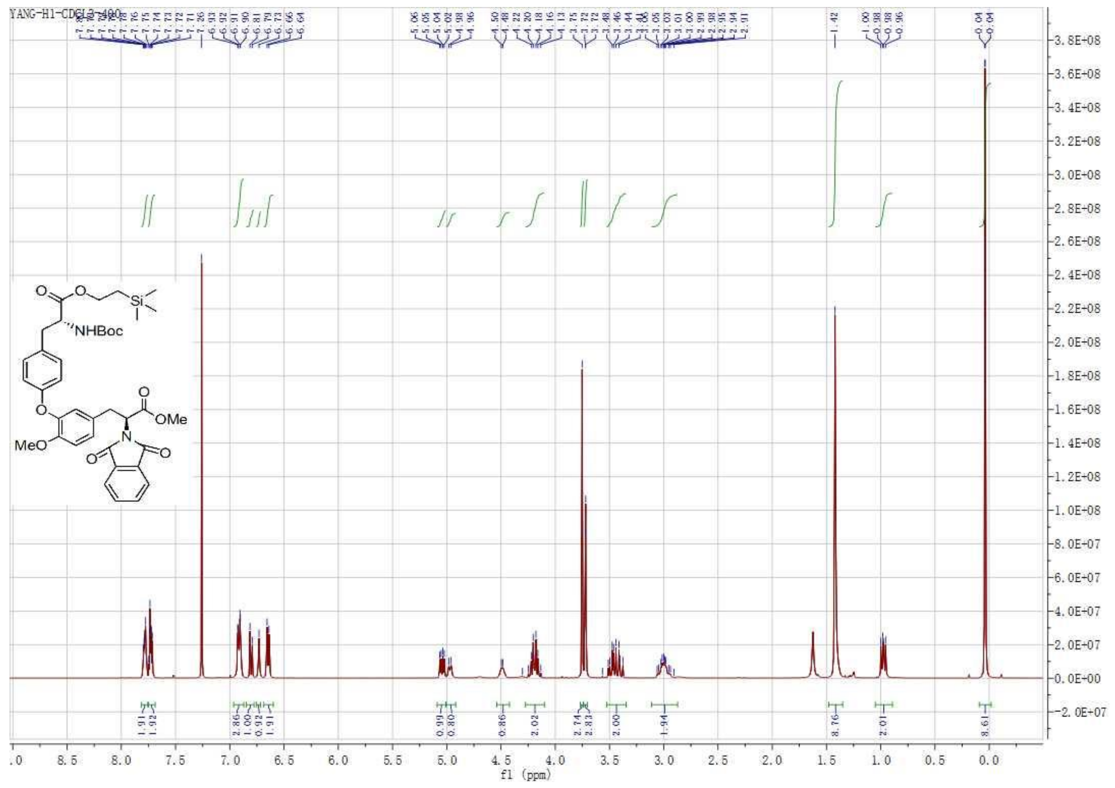

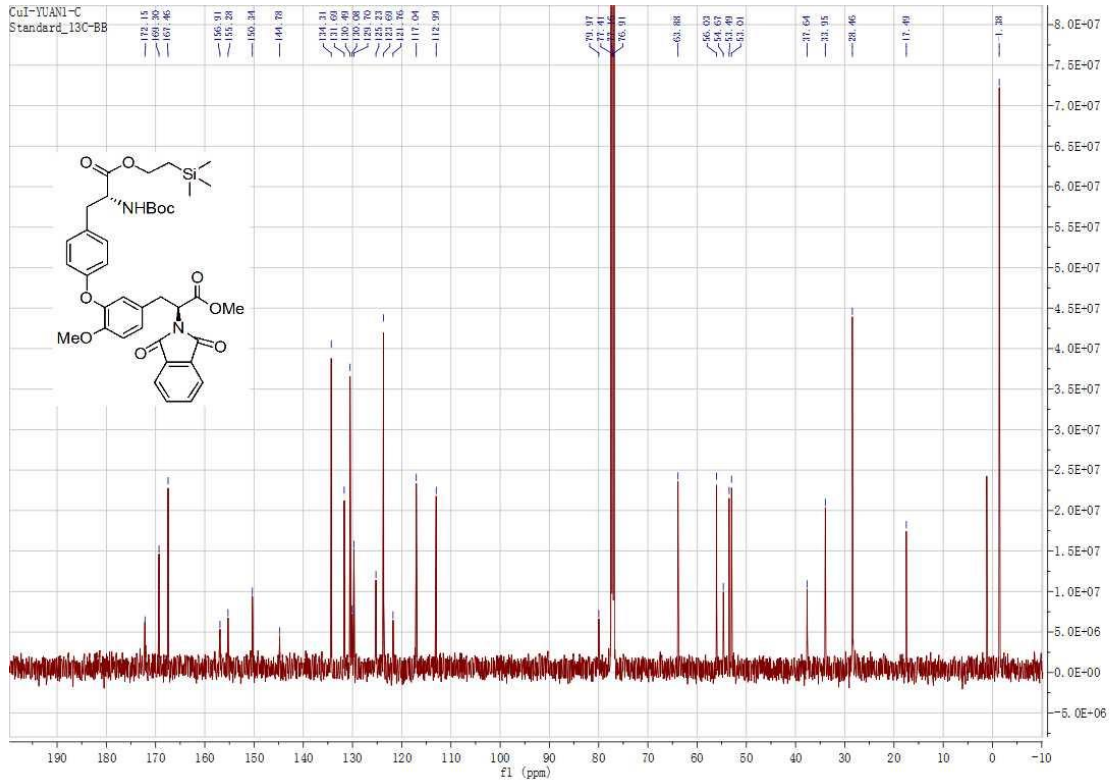

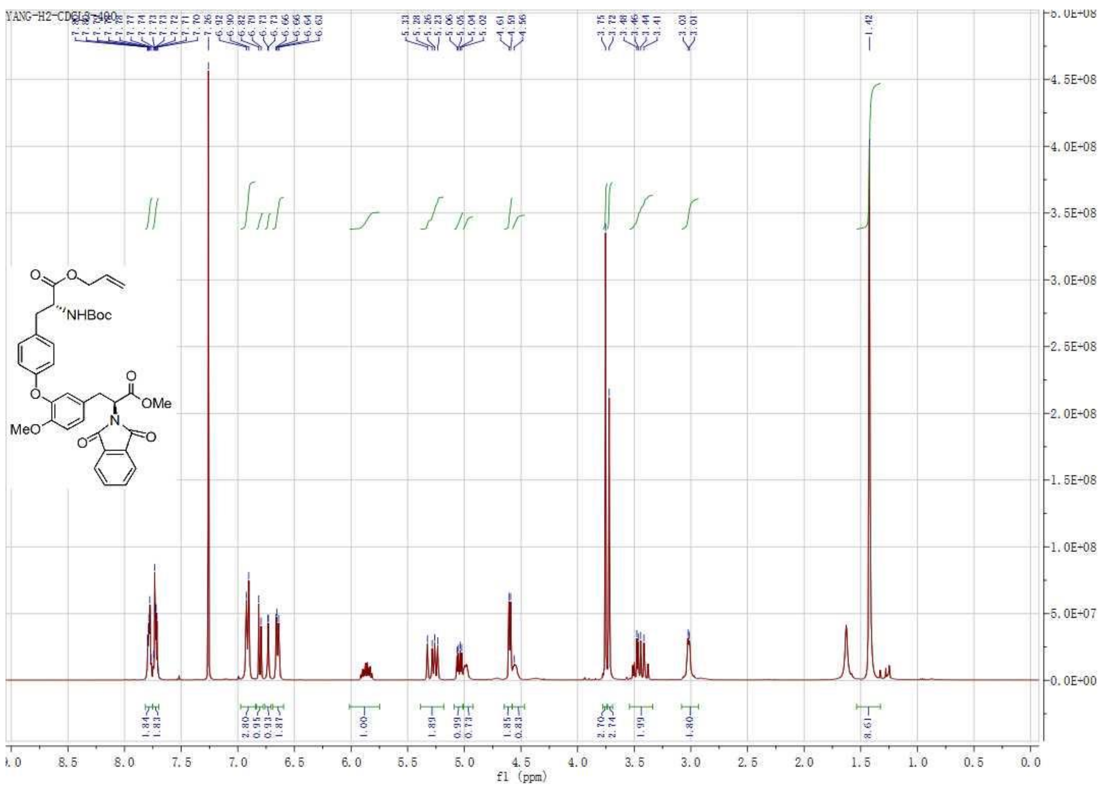
YANG-H2-CDCl3-400
NHBoc
OMe
MeO
f1 (ppm)

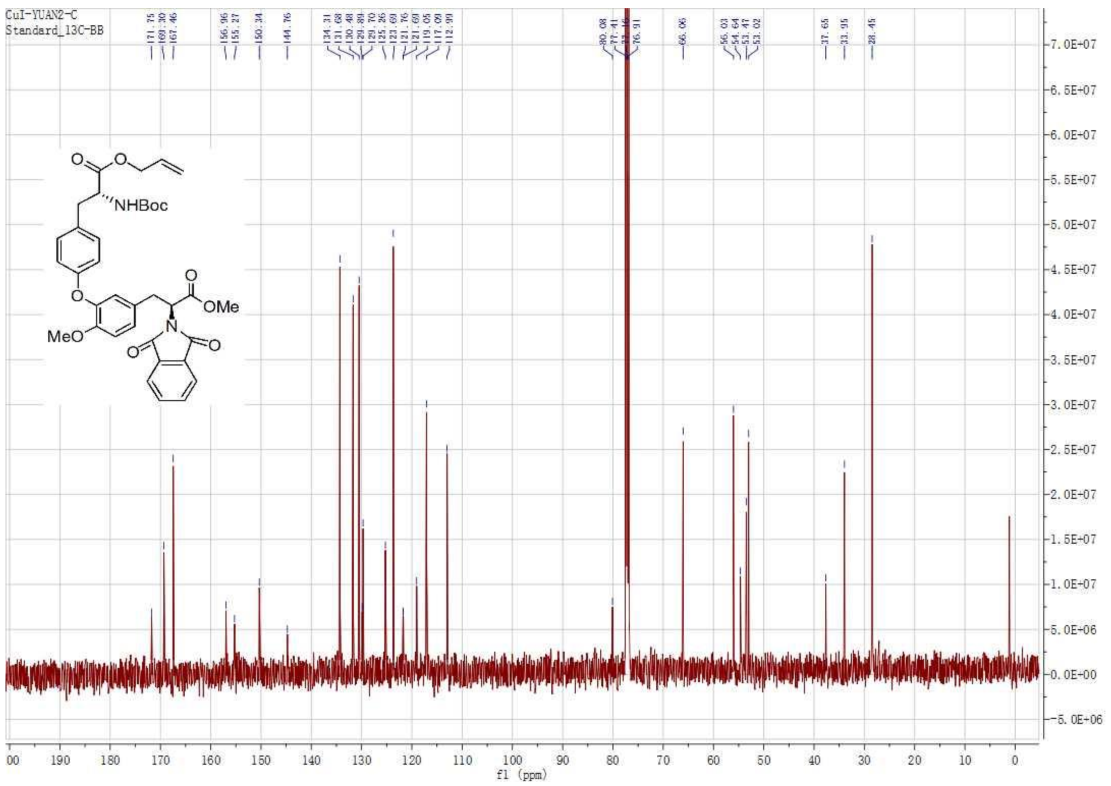
CuI-YUAN2-C
Standard_13C-BB
NHBoc
OMe
MeO
f1 (ppm)

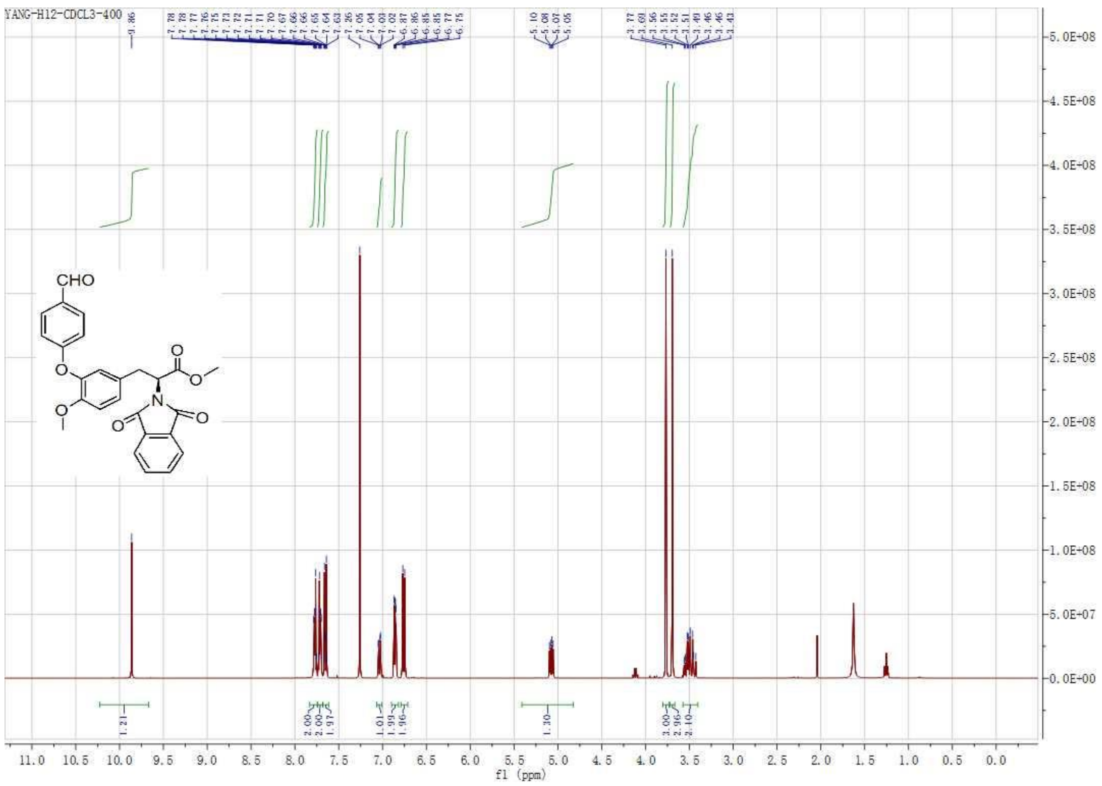
YANG-H12-CDCL3-400
CHO
O
O
O
N
O
O
f1 (ppm)

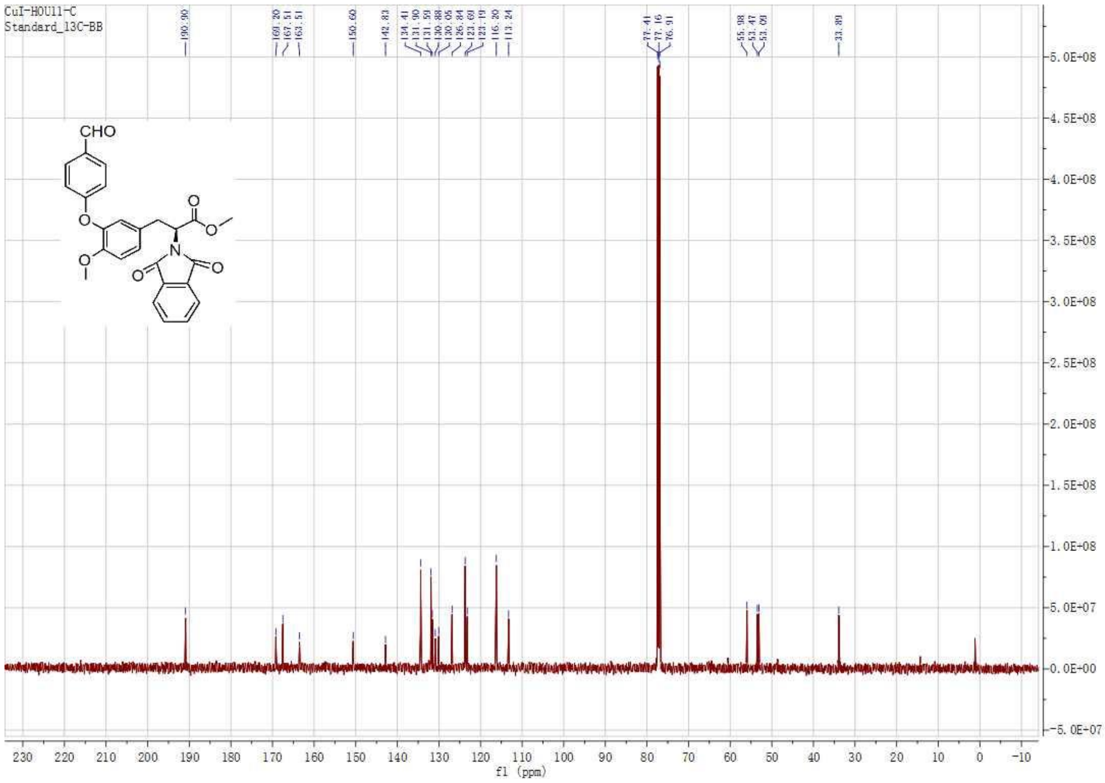
CuI-HOU11-C
Standard_13C-BB
CHO
O
O
O
N
O
O
f1 (ppm)

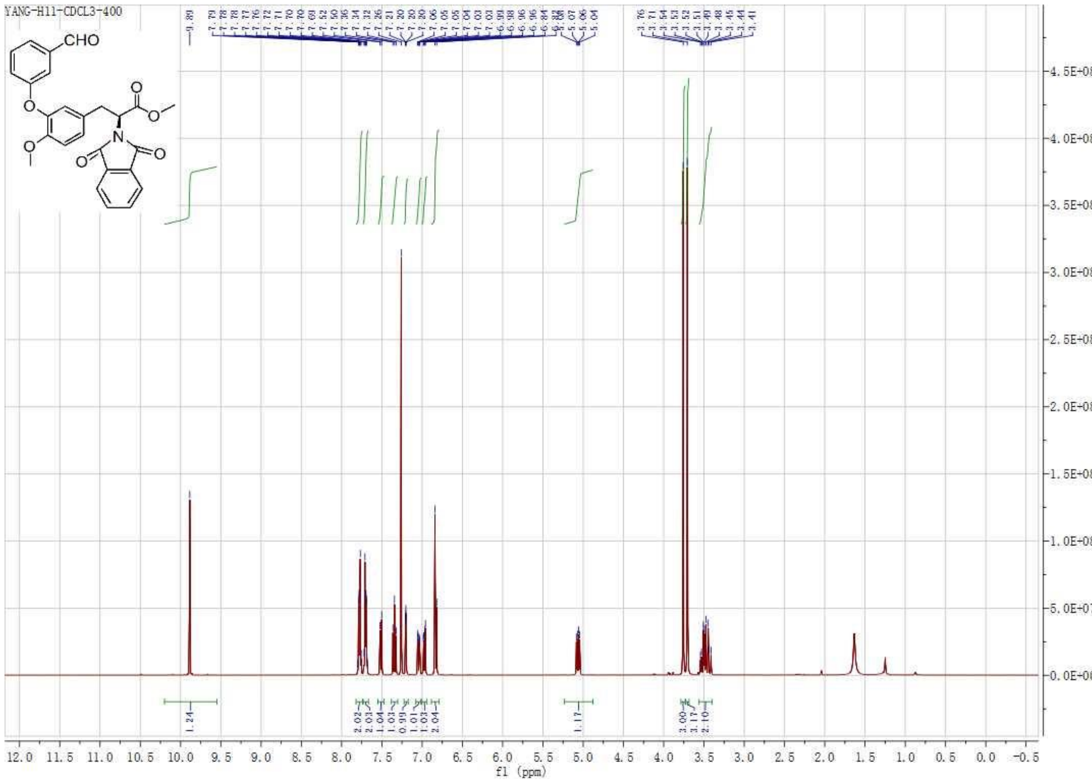
YANG-H11-CDCL3-400
CHO
f1 (ppm)

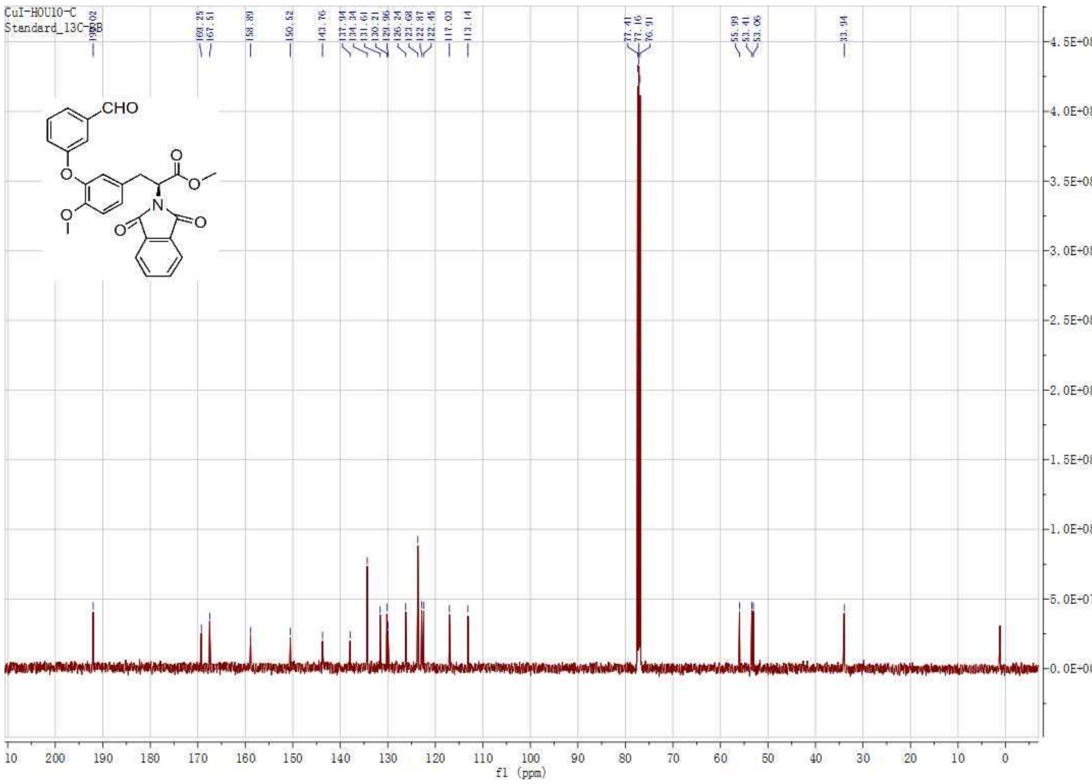
CuI-HOU10-C
Standard_13C-BB
CHO
f1 (ppm)

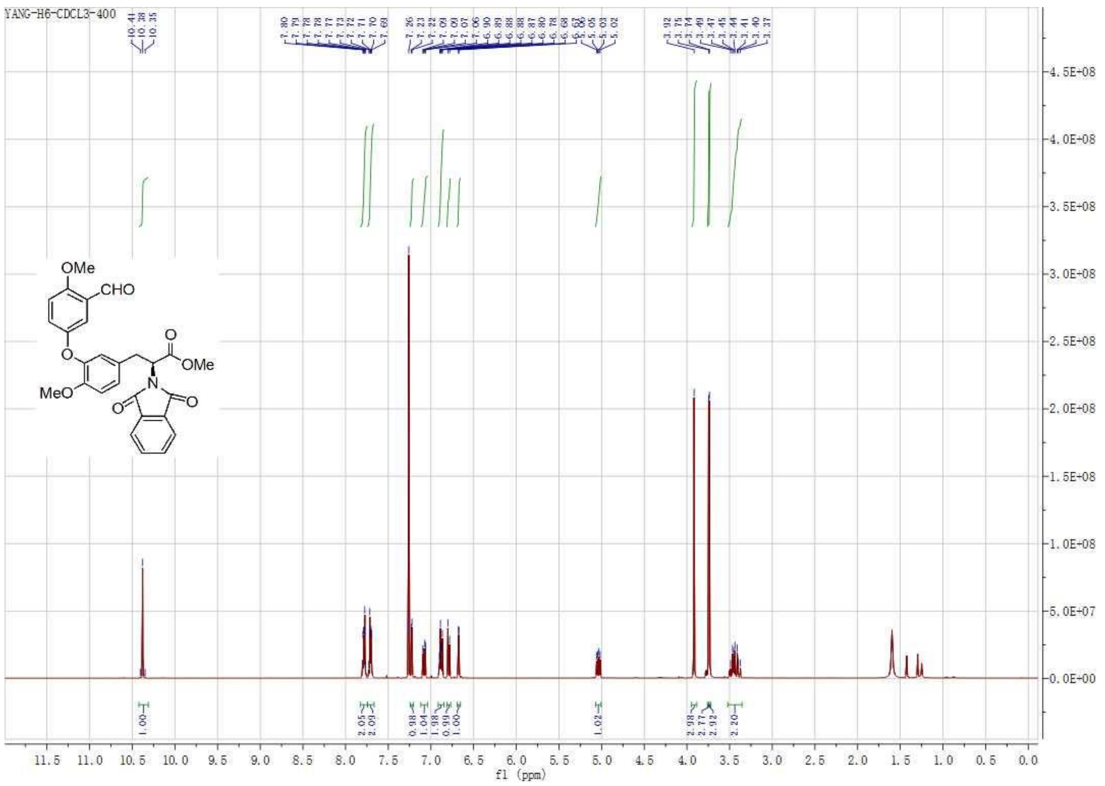

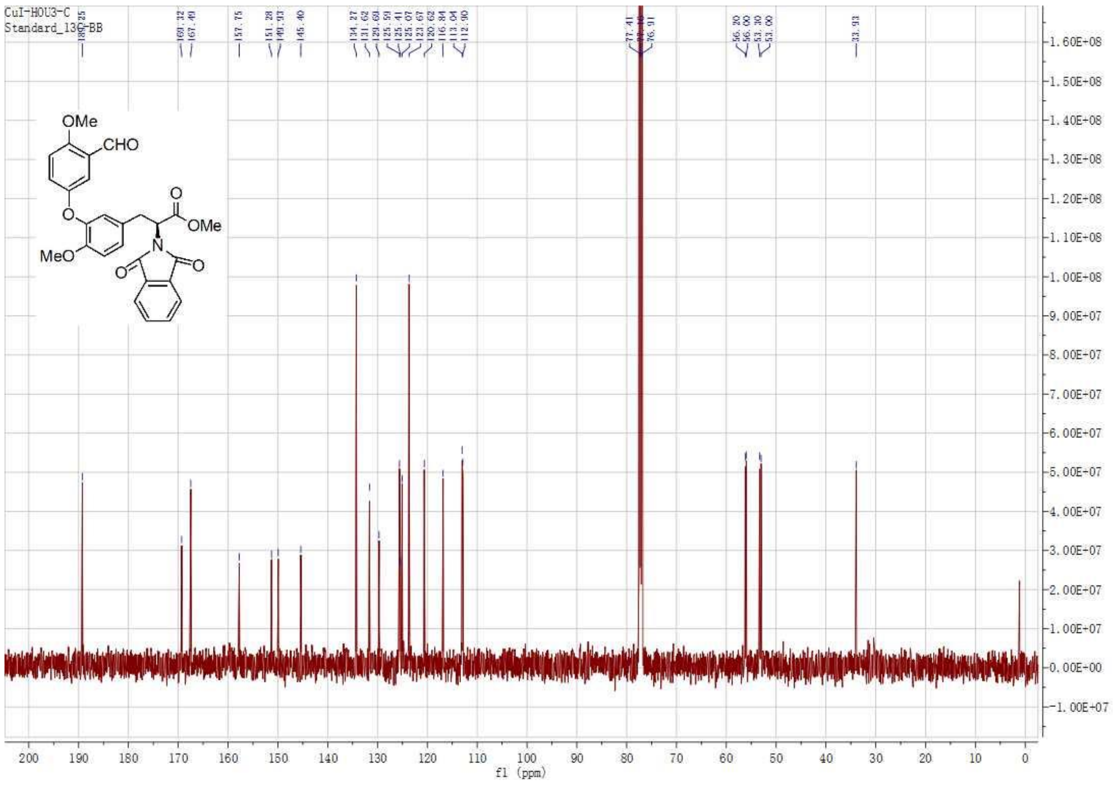

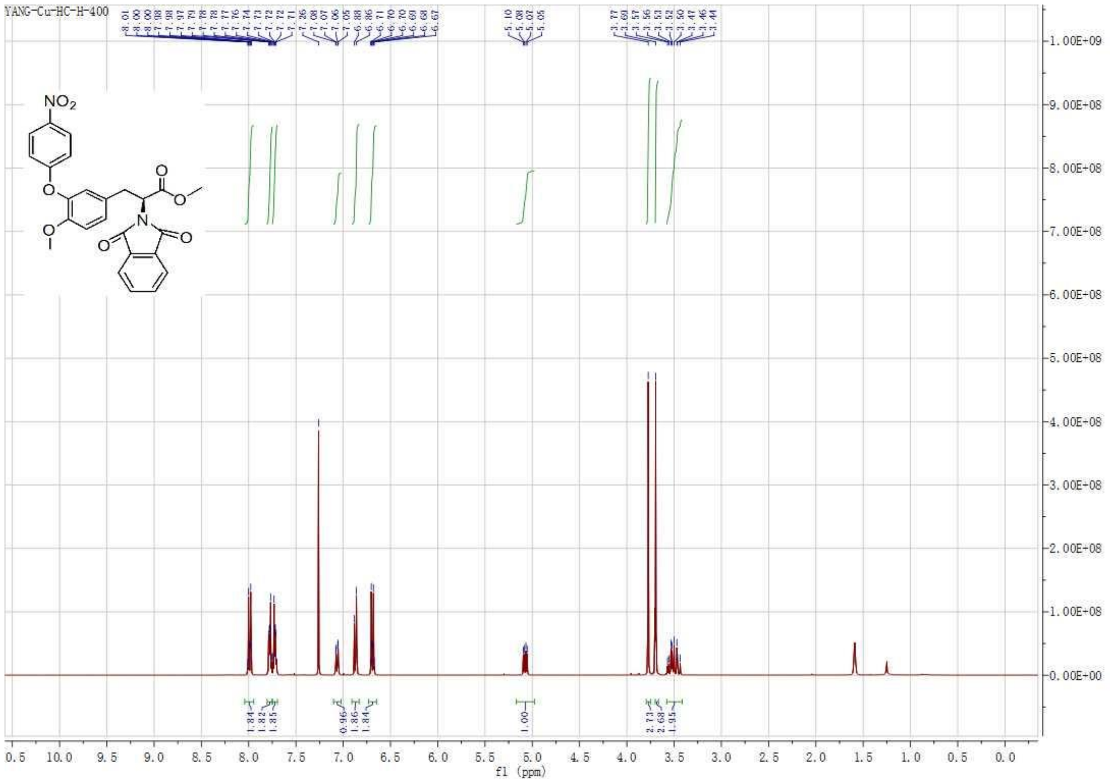
YANG-Cu-HC-H-400
fl (ppm)

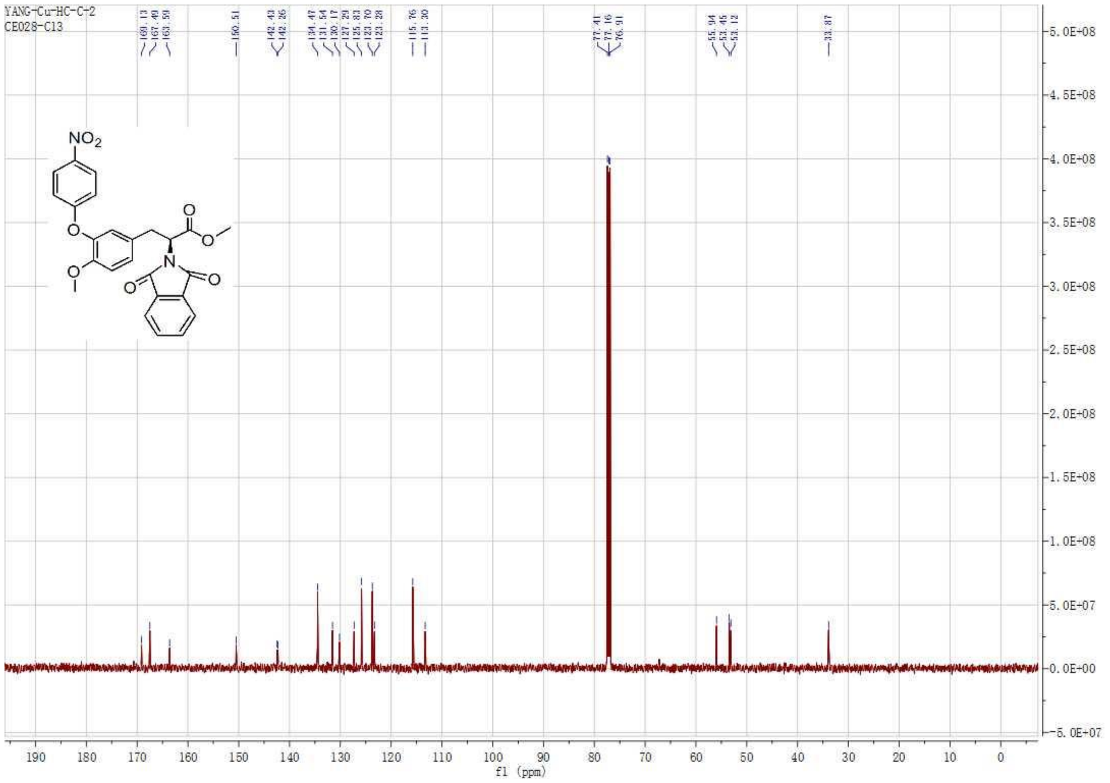
YANG-Cu-HC-C-2
CE028-C13
fl (ppm)

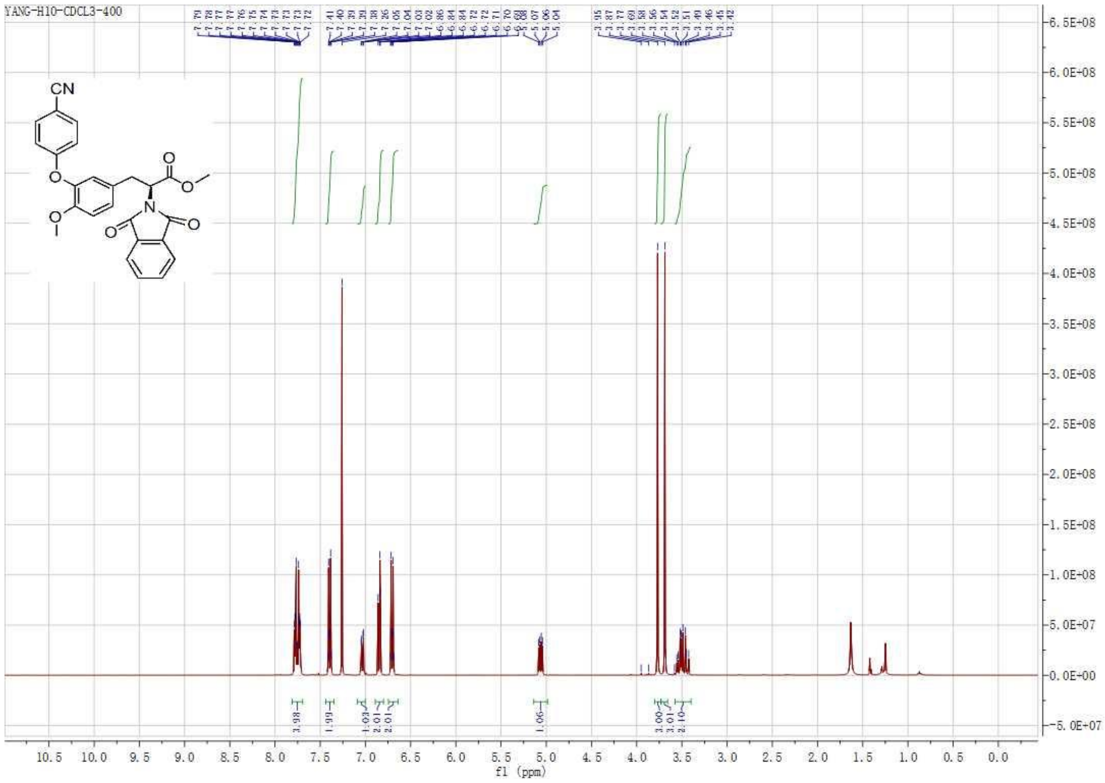

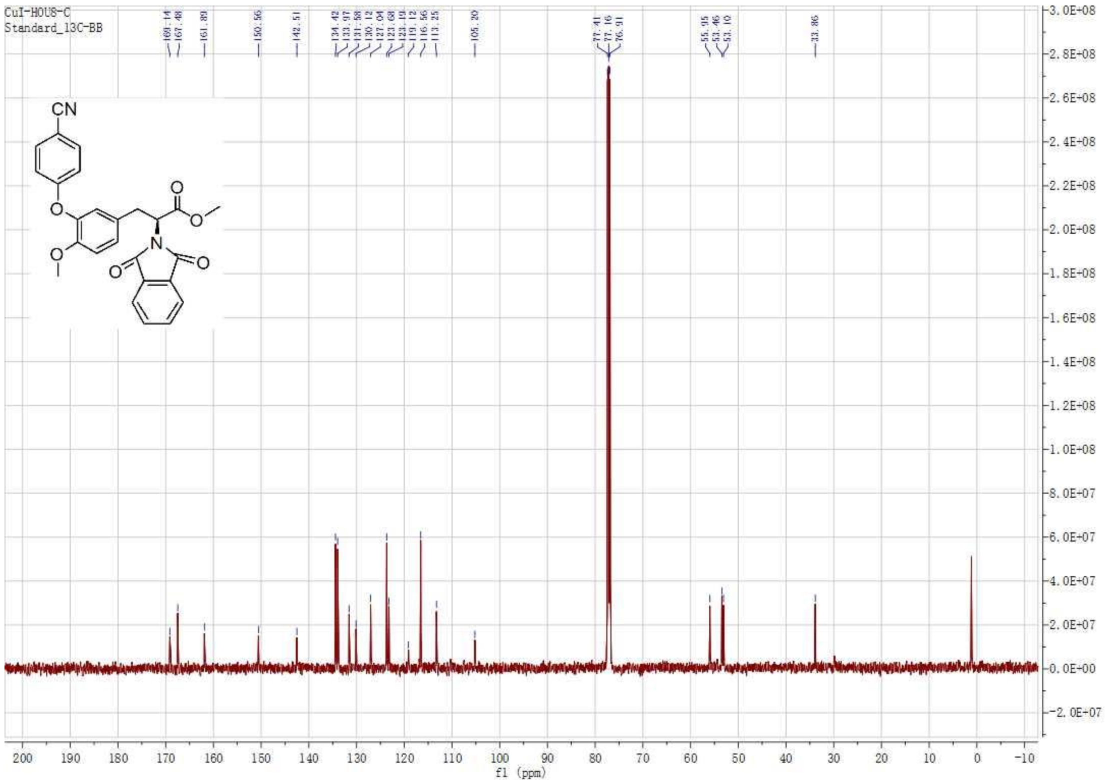

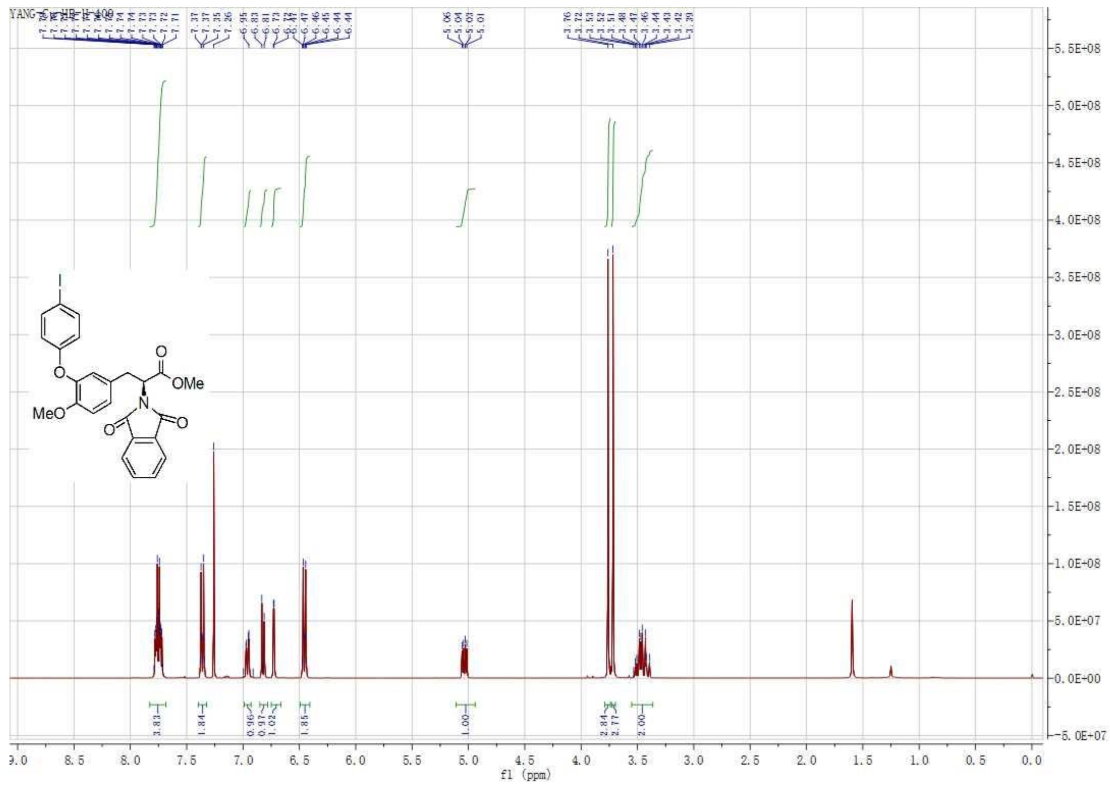

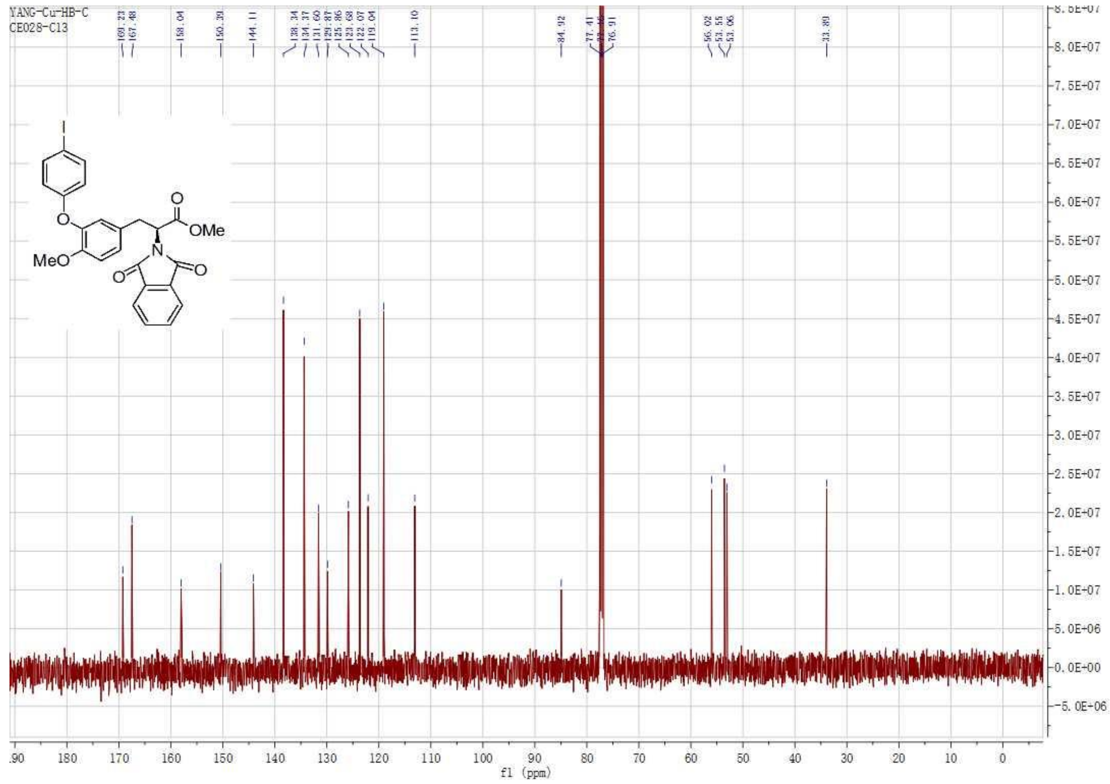

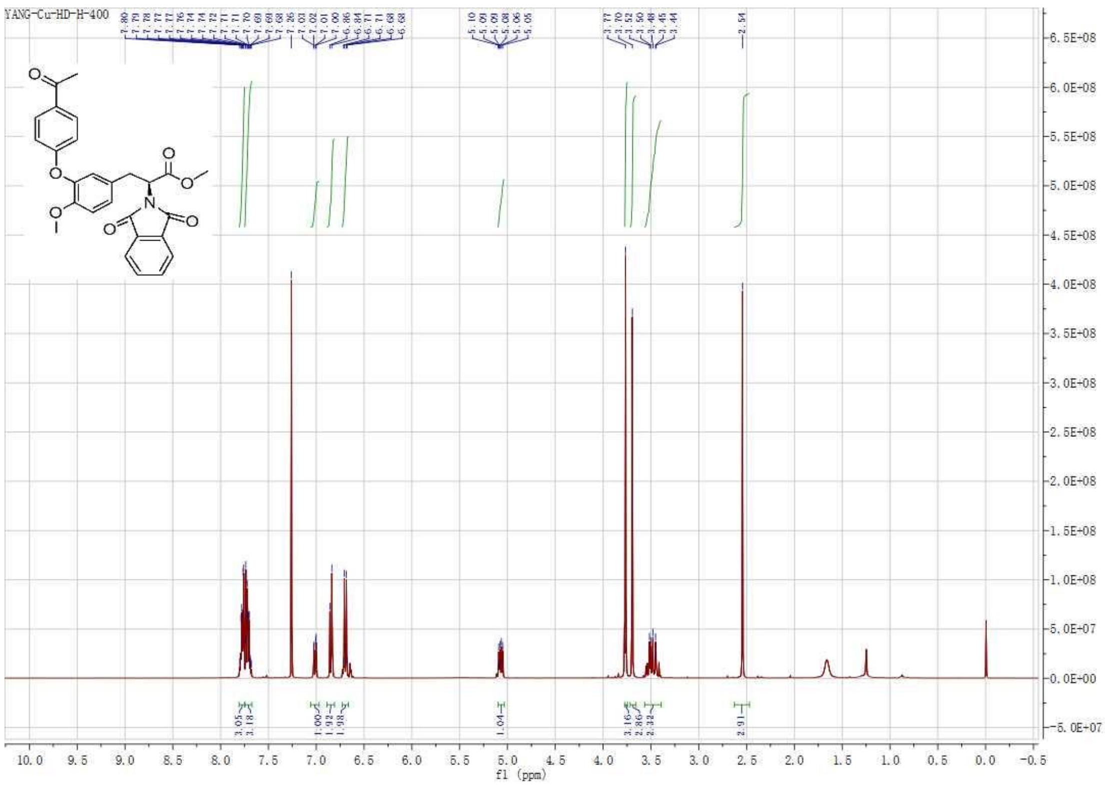
YANG-Cu-HD-H-400
f1 (ppm)

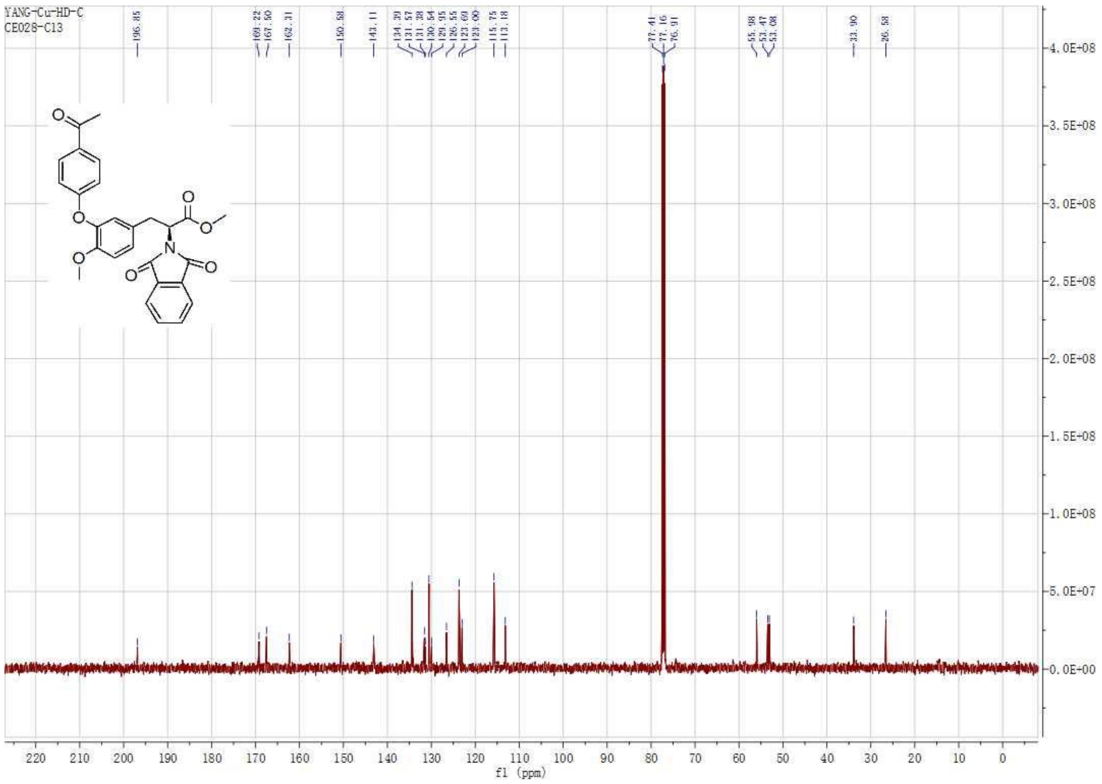
YANG-Cu-HD-C
CE028-C13
f1 (ppm)

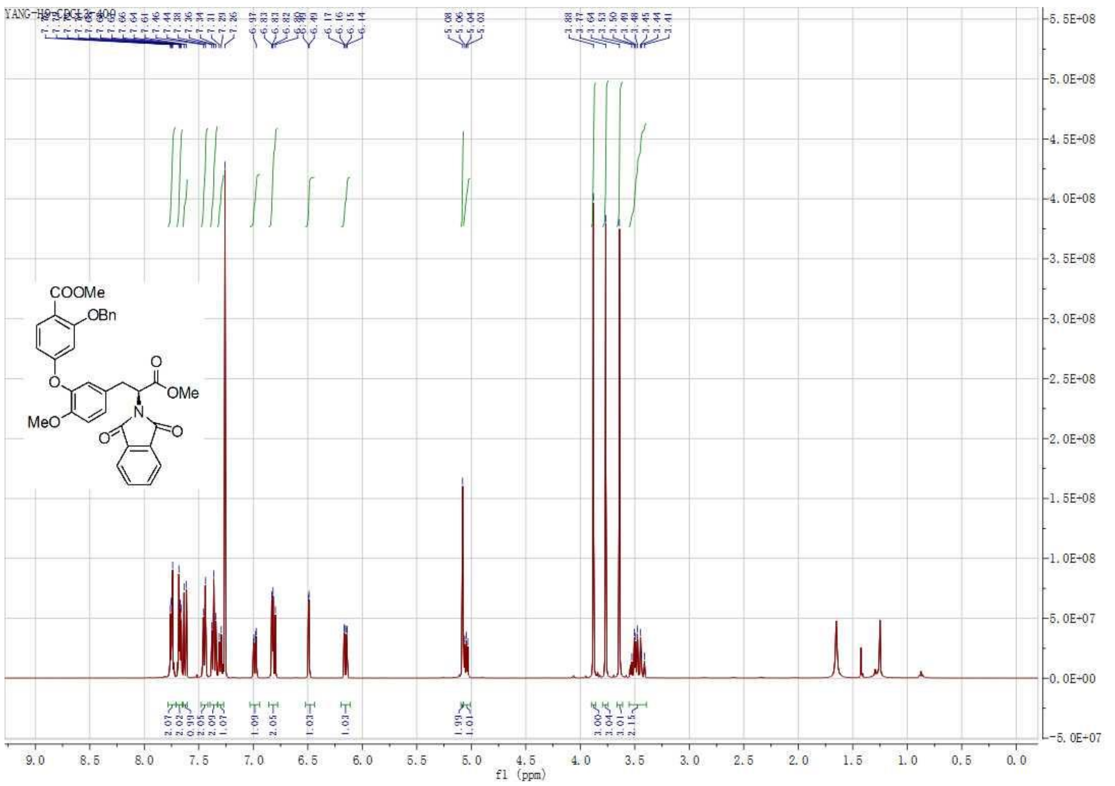

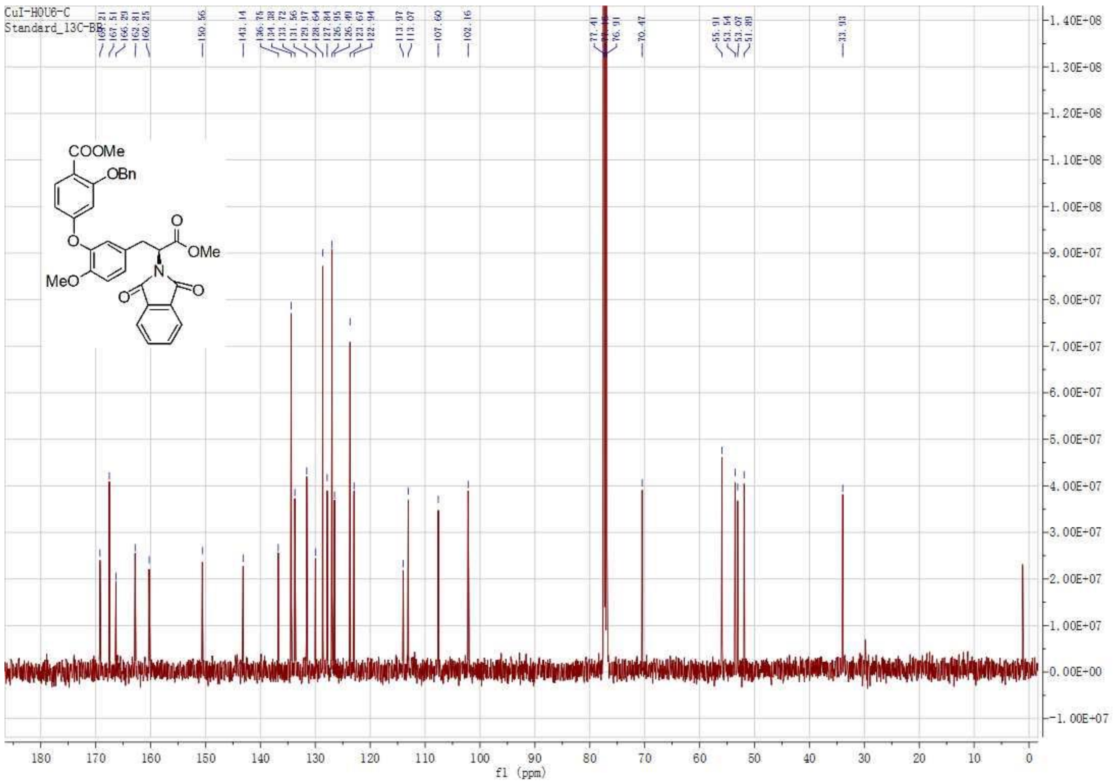

Supplement: Supplementary file 1 [file marinedrugs-21-00373-s001.zip › marinedrugs-2463132-supplementary.pdf]
